# Dual regulatory mechanisms of *YGP1* expression in response to glucose availability in *Saccharomyces cerevisiae*

**DOI:** 10.1371/journal.pone.0355681

**Published:** 2026-08-07

**Authors:** Megumi Sato, Kaoru Irie, Yasuyuki Suda, Tomoaki Mizuno, Kenji Irie

**Affiliations:** 1 Doctoral Program in Medical Sciences, Graduate School of Comprehensive Human Science, University of Tsukuba, Japan; 2 Laboratory of Molecular Cell Biology, Institute of Medicine, University of Tsukuba, Tsukuba, Japan; Tulane University Health Sciences Center: Tulane University School of Medicine, UNITED STATES OF AMERICA

## Abstract

Cells adapt to fluctuating nutrient conditions by dynamically regulating gene expression, ensuring survival under stress. Ygp1, a secretory yeast glycoprotein, is one such gene that is induced by nutrition deprivation, particularly glucose starvation. In this study, we investigated the regulatory mechanisms underlying nutrition-deprivation-responsive changes in gene expression, focusing on *YGP1* expression. Under glucose-rich conditions, *YGP1* expression was positively regulated by the RNA-binding protein Puf5, a member of the Puf family. This regulation ensured rhythmic *YGP1* expression during M phase of the cell cycle. The Puf5-mediated control targeted a specific 60-nucleotide region in the *YGP1* promoter (−600 to −540 from the start codon), and this regulation was partly mediated by the acid stress-responsive transcriptional activator Haa1. In addition, upon glucose exhaustion (diauxic shift), *YGP1* expression was strongly induced by the stress-responsive transcription factors Msn2 and Msn4 through the stress-response elements in the *YGP1* promoter. Further analysis of the physiological significance of *YGP1* expression revealed that the Puf5-mediated regulation contributes to the acid stress responses, and *YGP1* expression supports cell survival in the *puf5*Δ background. In summary, *YGP1* expression is regulated by two distinct factors in a glucose availability-dependent manner: Puf5 under glucose-rich conditions and the Msn2/Msn4 during glucose starvation. Especially, Puf5-mediated regulation contributes to the acid stress responses and subsequently supports long-term cell survival.

## Introduction

Cells survive changing environments by finetuning gene expressions to maintain cellular homeostasis. Nutrient availability is one of the major challenges. *Saccharomyces cerevisiae* regulates cell growth and cell cycle progression responding to nutrition conditions [1]. Carbon source availability is a key determinant of yeast growth. Under glucose-rich conditions, yeast cells grow logarithmically via fermentation, accompanied by ethanol accumulation. When glucose in the medium is depleted, cells switch from glucose to ethanol as a carbon source and enter a respiratory growth phase. This transition, known as the diauxic shift, is characterized by dynamic transcriptome changes [2,3]. Entry into the stationary phase is regulated by the PKA, TOR, and AMPK pathways [4–6]. As major downstream effectors, Msn2 and Msn4 function as general stress-responsive transcription factors [7,8]. They bind to the stress-responsive element (STRE; 5′-CCCCT-3′) in gene promoters and regulate the STRE-controlled genes [9]. Under non-stress conditions, Msn2 and Msn4 localize to cytoplasm, but upon exposure to kinds of stresses including nutrient depletion, organic acid, osmotic, or heat stress, they translocate to the nucleus to regulate the expression of environmental stress responsive genes [10–12]. Glucose starvation is a major signal inducing this transcriptional response, as Msn2 and Msn4 rapidly accumulate in the nucleus in response to glucose depletion [10,12]. Furthermore, this translocation is mainly driven by reduced PKA activity [10,12], with the yeast AMPK Snf1 and the phosphatase PP1 contributing in a supportive manner [13].

*YGP1* is one of the genes whose expression is strongly induced under nutrient deprivation [14,15]. *YGP1* was first identified as a highly glycosylated secretory protein (Yeast Glyco-Protein); its coding region contains 14 N-glycosylation sites and 11 carbohydrate modification sites [14]. *YGP1* expression is induced by depletion of glucose, nitrogen, and phosphate [14] and is strongly upregulated upon entry into the stationary phase [15]. Furthermore, *YGP1* expression was reported to be rhythmically induced during M-phase of the cell cycle [16] and Ygp1 protein is highly secreted during cell wall regeneration [17]. In the flor yeast, Ygp1 protein is required for glucose-deprivation-responsive biofilm formation, which is essential for wine elaboration process [18]. Moreover, in the engineered yeast that artificially expresses β-glucosidase on the cell surface, deletion of *YGP1* enhanced β-glucosidase activity and increased ethanol production by influencing the expression of cell wall-related genes [19]. Several studies have also demonstrated the involvement of Ygp1 in the acid stress responses: *YGP1* expression is activated by the acid stress-responsive transcriptional activator Haa1, thereby contributing to acid stress tolerance [20–22]. In addition, microarray analysis during acid stress responses revealed the induction of *YGP1* expression in response to lactic acid, acetic acid, and hydrochloric acid [23]. While these studies have highlighted the potential role of extracellular Ygp1 in cell wall maintenance and extracellular stress responses including glucose deprivation and acid stress, the detailed regulatory mechanisms and physiological significance of *YGP1* induction remain unclear.

In this study, we investigated the regulatory mechanisms of *YGP1* expression and found two distinct pathways controlling its expression depending on glucose availability. Under glucose-rich conditions, *YGP1* expression is positively regulated by the RNA-binding protein Puf5 through its promoter, partly mediated by the transcriptional activator Haa1. This Puf5-mediated regulation not only induces M-phase-specific *YGP1* expression during cell cycle but also contributes to the acid stress responses, subsequently supporting long-term cell survival. Under glucose-deficient conditions, the expression is strongly induced at the diauxic shift by Msn2 and Msn4. This is the first report identifying Puf5 as an upstream regulator of *YGP1* expression, and this Puf5-mediated regulation contributes to acid stress responses and long-term survival. Our findings underscore the importance of the regulatory machinery governing secreted proteins in adapting to changing environments.

## Materials and Methods

### Yeast strains

The *Saccharomyces cerevisiae* strain used was W303 in this study. All acquired strains from original W303 strain were listed in S1 Table. For the DNA manipulation, *Escherichia coli,* DH5α strain, was used.

### Cell culture

Yeast strains were pre-cultured in YPD medium (2% Glucose, 2% bactopeptone, and 1% yeast extract) and then shifted to main culture in YPD medium. Strains harboring plasmids were cultured in selective synthetic complete (SC) media according to the corresponding auxotrophic marker: SC-Ura medium for YCplac33 and YEplac195 plasmids, and SC-Leu medium for the YEp13 plasmid. The strains carrying a YCplac33-*YGP1* or YCplac33-*YGP1*–3HA plasmid were pre-cultured in SC-Ura medium and then transferred to the main culture in YPD medium. Since the diauxic shift response was not clearly observed in SC medium, YPD medium was used for the main culture. For investigating non-fermentative growth, cells were incubated on a YPGlylac plate (3% Glycerol, 2% Sodium Lactate, 2% bactopeptone, and 1% yeast extract). All cell cultures were performed at 28 degrees. Samples were collected at the time set for each experiment.

### Measurement of Glucose concentration

Glucose concentrations in the culture medium were measured using a Glucose (GO) Assay Kit (Sigma-Aldrich). Absorbance at 540 nm was measured with a Varioskan LUX microplate reader.

### Gene deletion

All gene deletions were performed as previously described [24–26]. All primers used were listed in S2 Table. The amplified fragments by PCR were transformed into diploid cells, and transformants were selected on SC medium lacking each selective amino acid.

### Plasmids construction

The plasmids used in this study were listed in S3 Table. All plasmids were constructed by inserting the indicated fragments between the *EcoR*I and *Sal*I sites of the YCplac33 plasmid. YCplac33-*YGP1* plasmid was constructed by inserting the *YGP1*promoter-ORF-3’UTR fragment. The YCplac33-*MCM2*promoter-*GFP*-*ADH1* 3’UTR and YCplac33-*MCM2*promoter-*GFP*-*YGP1* 3’UTR plasmids were constructed by inserting fragments containing the *MCM2* promoter, *GFP*, and the corresponding 3′ UTR. For the construction of a YCplac33-*YGP1*–3HA plasmid, the *YGP1*promoter-ORF, 3HA, and *YGP1* 3’UTR fragments were inserted. A series of *YGP1* promoter deletion plasmid was constructed by inserting two fragments comprising an upstream promoter region and a downstream promoter-*YGP1* ORF-3’UTR fragment. YCplac33-*YGP1*-STRE1del and YCplac33-*YGP1*-STRE2del plasmids were constructed by inserting two fragments (upstream or downstream fragments from each STRE). The primers used for the amplification of fragments were listed in S4 Table.

### RNA extraction and quantification

**Fig 1:**
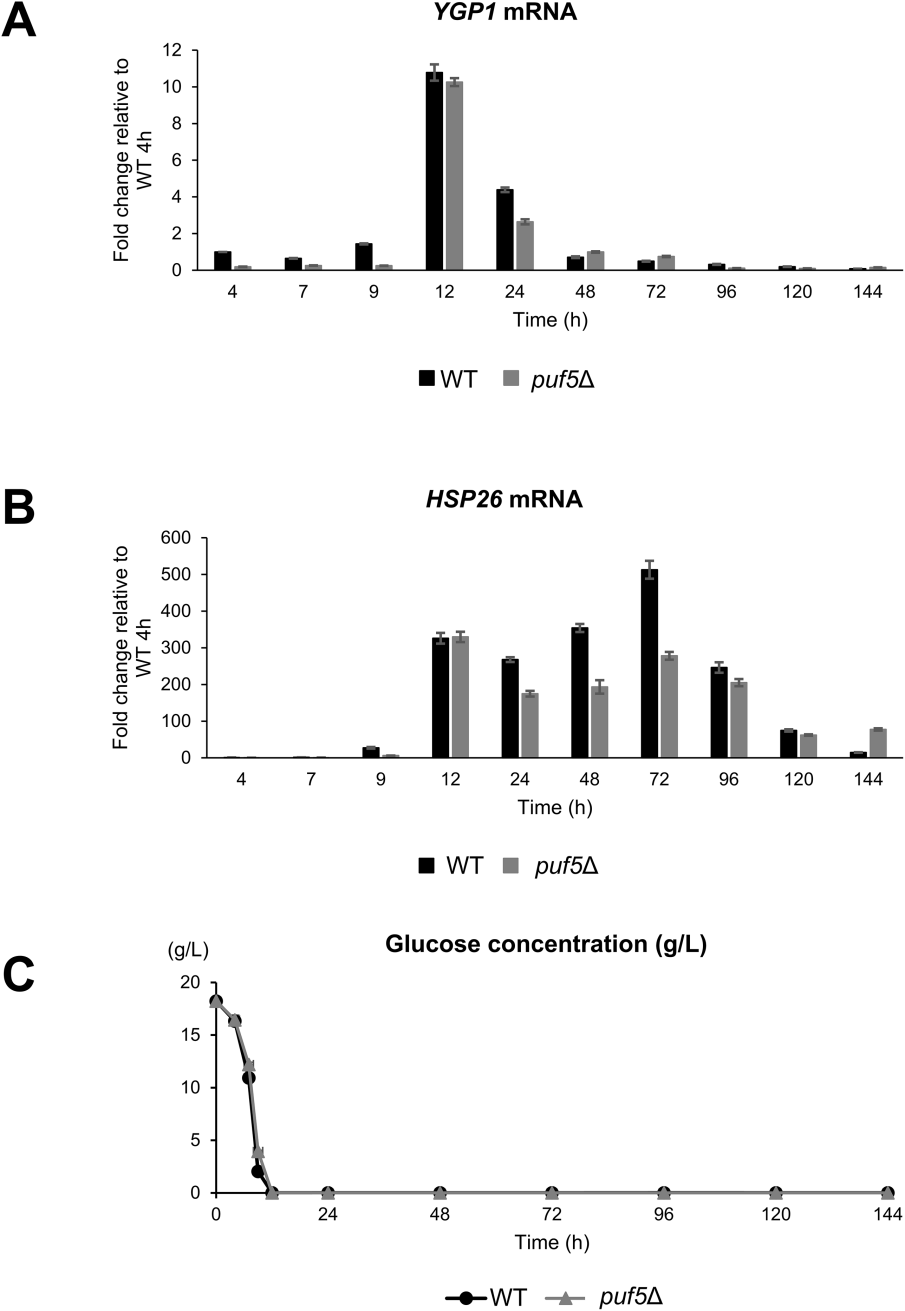
*YGP1* expression is highly induced at diauxic shift. (A, B) The fold change of *YGP1* (A) and *HSP26* mRNA levels (B) in the wild-type and *puf5*Δ mutant along a time course relative to the wild-type 4 hours value. The expression pattern was confirmed using two biological replicates, each having three technical replicates. The data show the average Rq values ± SEM (n = 3 technical replicates from one representative biological replicate). (C) Yeast cells were pre-cultured and then transferred to YPD medium for the main culture. Glucose concentrations were measured at the indicated time points. For each strain, glucose levels were measured six times at each time point, and the mean value was taken as the glucose concentration. The graph shows the mean glucose concentrations ± SEM (n = 3 biological replicates each having n = 6 technical replicates).

Total RNA samples were extracted utilizing ISOGEN reagent (Nippon Gene), followed by removal of genome DNA and reverse transcription using PrimeScript™ RT reagent Kit with gDNA Eraser (Perfect Real Time) (Takara). The expression of cDNA was quantified RT-qPCR using QuantStudio 5 for Fig 1 and QuantStudio 7 (Thermo Fisher Scientific) for other figures with SYBR Premix Ex Taq (Takara). Relative expression levels were calculated using the ΔΔCt method, with *SCR1* used as the endogenous control. Rq values were expressed as fold changes relative to the indicated reference sample. The primers used for the amplification were listed in S5 Table. The *YGP1*–3HA mRNA was differentiated from endogenous *YGP1* mRNA by using a set of primers from 3HA-tag sequences. All graphs were created by Microsoft Excel.

### Protein extraction and quantification

Protein extraction was performed as previously described [27]. Protein samples were loaded onto a 12% SDS-PAGE gel for Ygp1–3HA and a 10% SDS-PAGE gel for Pgk1. The gel was transferred to a PDVF membrane (Millipore), and then membrane was reacted with the primary antibody, the anti-HA monoclonal antibody HA11 or anti-Pgk1 antibody, at 4°C overnight. Reacted with the secondary antibody, the anti-mouse IRDye® 800CW secondary antibodies and IRDye® 680RD secondary antibodies (LI-COR), the visualization and quantification were performed using ODYSSEY CLx (LI-COR). The acquired relative signal intensities of Ygp1–3HA protein were normalized by those of Pgk1, an endogenous control. All graphs were created by Microsoft Excel.

### Cell cycle synchronization

Cell cycle synchronization was performed by α-factor induced G1-phase arrest and releasing as previously described [28]. The synthesized α-factor was purchased from GenScript and dissolved into sterilized water to 20 mM. Each *bar1*Δ background strain was exposed to 30 nM α-factor and reacted for 2 hours in YPD. After washing with YPD, samples were collected every 10 minutes.

### Measurement of cell viability

Cells were cultured in SC medium until an appropriate time and diluted to final OD_600_ = 2. Cell viability was counted using NucleoCounter® YC-100 (M&S TechnoSystems).

### Statistical analysis

**Fig 2:**
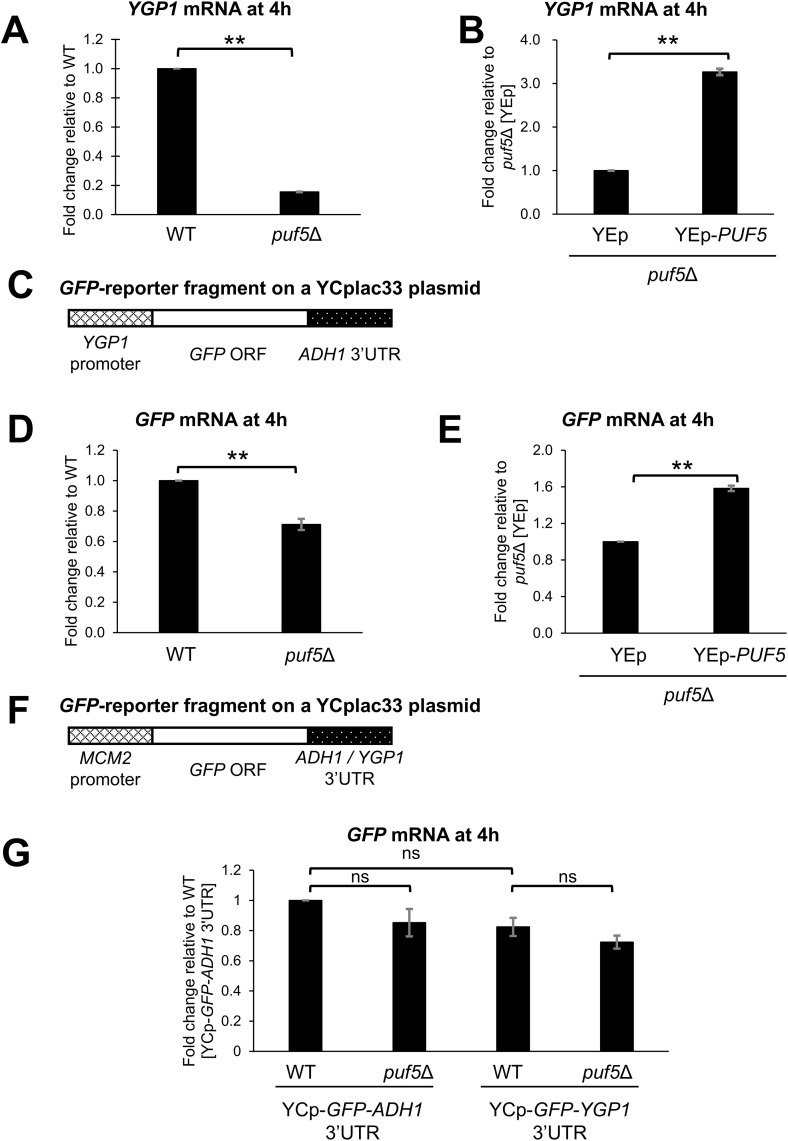
*YGP1* expression is regulated by Puf5 through its promoter. (A) The fold change of *YGP1* mRNA level in the *puf5*Δ mutant relative to that in wild-type strain. The expression pattern was confirmed using three biological replicates, each having three technical replicates. The data show the average Rq values ± SEM (n = 3 technical replicates from one representative biological replicate). **p < 0.01 is indicative of statistical significance. (B) The fold change of *YGP1* mRNA level in the *puf5*Δ [YEplac195-*PUF5*] strain relative to that in *puf5*Δ [YEplac195 vector]. The expression pattern was confirmed by three biological replicates, each having three technical replicates. The data show the average Rq values ± SEM (n = 3 technical replicates from one representative biological replicate). **p < 0.01 is indicative of statistical significance. (C) The scheme of *GFP* reporter fragment on a YCplac33 plasmid. The mesh bar represents *YGP1* promoter, the white bar does *GFP* ORF, and the black-dot bar does *ADH1* 3’UTR. (D) The fold change of *GFP* mRNA level in the *puf5*Δ mutant relative to that in wild-type strain harboring YCplac33-*GFP*-reporter plasmid. The expression pattern was confirmed by two biological replicates, each having three technical replicates. The data show the average Rq values ± SEM (n = 3 technical replicates from one representative biological replicate). **p < 0.01 is indicative of statistical significance. (E) The fold change of *GFP* mRNA level in the *puf5*Δ [YCplac33-*GFP*-reporter] [YEplac13-*PUF5*] strain relative to that in *puf5*Δ [YCplac33-*GFP*-reporter] [YEplac13 vector]. The expression pattern was confirmed by two biological replicates, each having three technical replicates. The data show the average Rq values ± SEM (n = 3 technical replicates from one representative biological replicate). **p < 0.01 is indicative of statistical significance. (F) The scheme of *GFP* reporter fragment on a YCplac33 plasmid. The mesh bar represents *MCM2* promoter, the white bar does *GFP* ORF, and the black-dot bar does *ADH1* 3’UTR or *YGP1* 3’UTR. (G) The fold change of *GFP* mRNA level in the WT and *puf5*Δ mutant harboring a [YCplac33 *MCM2*promoter-*GFP-ADH1* 3’UTR] or [YCplac33 *MCM2*promoter-*GFP-YGP1* 3’UTR] plasmid relative to that in WT [YCplac33 *MCM2*promoter-*GFP-ADH1* 3’UTR]. The data show the average Rq values ± SEM of three biological replicates each having three technical replicates. ns is indicative of no statistical significance (*p* < 0.05).

Biological replicates were defined as independent cultures initiated separately. Technical replicates were defined as repeated measurements performed on the same biological sample, including independent qPCR reactions and Western blot quantifications. For each experiment, analyses were performed using two independent biological replicates, each consisting of three technical replicates. Data in the graphs are presented as the mean ± SEM of the three technical replicates from a single representative biological replicate, unless stated otherwise. Exceptions to this replication scheme are as follows: for Figs 1C and 2G, three biological replicates were analyzed; for Figs 5B-C, 9B, and S5 Figs, a single biological replicate was used as this experiment was conducted for screening purposes. Data shown represent he mean ± SEM of the three technical replicates. Statistical significance was determined using a Welch t-test for comparisons between two samples, and a Kruskal–Wallis test followed by a Dwass–Steel–Critchlow–Fligner test for comparisons between more than three samples, considering heteroscedasticity. *p* < 0.05 was considered as statistically significant. For the comparison of three or more groups with equal variances, homogeneity of variances was first assessed using Bartlett’s test, followed by one-way ANOVA. Statistical analysis of Fig 9E was performed using aligned rank transform (ART)–based multifactor ANOVA, with multiple comparisons corrected using Holm’s method.

## Results

### *YGP1* expression is controlled by dual regulatory pathways in response to glucose availability

Glucose availability is a major determinant of yeast growth, and yeast cells dynamically regulate gene expression in response to environmental glucose levels to modulate cell-cycle progression, metabolism, and stress responses [1–6]. *YGP1* is one of the genes whose expression is dynamically regulated under such conditions. Ygp1 is a secreted glycoprotein that is implicated in cell wall remodeling and potentially in stress responses triggered by environmental conditions such as glucose availability and acidic stress. Previous studies have reported that *YGP1* expression is strongly induced under nutrient starvation conditions, including glucose deprivation [14,15]. Based on these observations, we hypothesized that the induction of *YGP1* expression may contribute to the cellular responses to glucose starvation. In this study, we investigated the regulatory mechanisms governing *YGP1* expression. We first examined the *YGP1* expression pattern along a time course in YPD medium. *YGP1* mRNA level peaked sharply at 12 hours in the wild-type and declined thereafter (Fig 1A). The expression of *HSP26*, a stationary phase marker [29], was induced at 12 hours and remained high for 3–4 days in the wild-type (Fig 1B). Investigating the glucose concentrations in the YPD medium, the wild-type strain exhausted glucose at 12h (Fig 1C), which is consistent with our previous report [30]. These results revealed that the expression of *YGP1*, which remained low during the glucose-replete logarithmic phase (4–9 h), was strongly induced at diauxic shift, and subsequently declined in the stationary phase. Given the pronounced glucose-dependent changes in expression, we analyzed the regulatory machinery of *YGP1* expression. Our previous microarray analysis under logarithmic growth conditions revealed that *YGP1* expression was 66% decreased in the *puf5*Δ mutant relative to the wild type [31] (Table 1). Puf5 encodes an RNA-binding protein regulating mRNA stability or translational efficiency through binding to 3’UTR region of its target mRNAs [32,33]. Accumulating evidence indicates that Puf5 targets *LRG1* mRNA, which encodes a Rho1 GAP involved in the yeast cell wall integrity pathway [34–36], thereby repressing its expression [30,37–39]. This effect is more evident in the stationary phase than in log phase [30], and *puf5*Δ mutants exhibit a shortened chronological life span associated with aberrant CWI signaling [37]. In addition, previous studies have demonstrated that Puf5 contributes to glucose starvation-induced recruitment to P-bodies and their degradation of cell wall-related mRNAs [40]. These observations imply that Puf5 functions in a glucose-responsive manner. Confirming the microarray result by qPCR, we first examined the time-course *YGP1* mRNA level in the *puf5*Δ mutant. *YGP1* expression was decreased in the *puf5*Δ mutant compared to the wild-type during the log phase (4–9 h) (Fig 1A), while it reached similar levels at 12 hours in the two strains (Fig 1A). Expression of *HSP26*, a marker of the stationary phase, was comparable between the *puf5*Δ mutant and wild-type cells at 12h (Fig 1B). Consistently, measurement of glucose levels revealed that the *puf5*Δ mutant consumed glucose at a similar rate to the wild-type (Fig 1C). These observations indicate that Puf5 does not contribute to the induction of *YGP1* expression at the diauxic shift but rather plays a specific role in the regulation of *YGP1* during logarithmic growth. This suggests the existence of dual regulatory pathways controlling *YGP1* expression under glucose-rich conditions and upon glucose depletion.

**Table 1:** Microarray results of *YGP1* in the wild-type and *puf5*Δ mutant.

gene	Count (Wild-type)	Count (*puf5*Δ)	Fold change
*YGP1*	1283	435.7	0.34

### *YGP1* expression is regulated by an RNA-binding protein Puf5 during the log phase

Aiming to clarify the mechanisms of the two distinct regulatory pathways of *YGP1* expression, we first focused on the glucose-rich mid-log phase (4h) and investigated how Puf5 positively regulates *YGP1* expression. Consistent with Fig 1A, *YGP1* expression was significantly decreased in the *puf5*Δ mutant (Fig 2A). We also found that the multicopy-*PUF5* increased the *YGP1* mRNA level (Fig 2B). To analyze the responsible region for the Puf5-mediated regulation, we constructed *YGP1* promoter-*GFP* reporter on a YCplac33 plasmid (Fig 2C) and investigated the expression of the *GFP* reporter. *GFP* expression showed similar expression pattern to *YGP1* expression: its expression was decreased in the *puf5*Δ mutant and increased by multicopy-*PUF5* (Figs 2D and E). While Puf5 influenced less efficiently on the *YGP1* promoter-*GFP*-reporter than on endogenous *YGP1* (Figs 2A, B, D, and E), the differences were significant (Figs 2D and E). To assess the contribution of the *YGP1* 3′ UTR to regulation mediated by Puf5, we introduced the YCplac33-*MCM2*promoter-*GFP*-*ADH1* 3’UTR and YCplac33-*MCM2*promoter-*GFP*-*YGP1* 3’UTR plasmids into wild-type and *puf5*Δ mutant and examined the *GFP* reporter expression levels. The expression levels of both the *GFP*-*ADH1* 3’UTR reporter and the *GFP*-*YGP1* 3’UTR reporter showed no significant difference between wild-type and *puf5*Δ mutant (Fig 2G). These results indicate that Puf5 positively regulates the expression of *YGP1* via its promoter, rather than its 3’UTR.

### *YGP1* expression is cyclically regulated during M-phase in a Puf5-mediated manner

**Fig 3:**
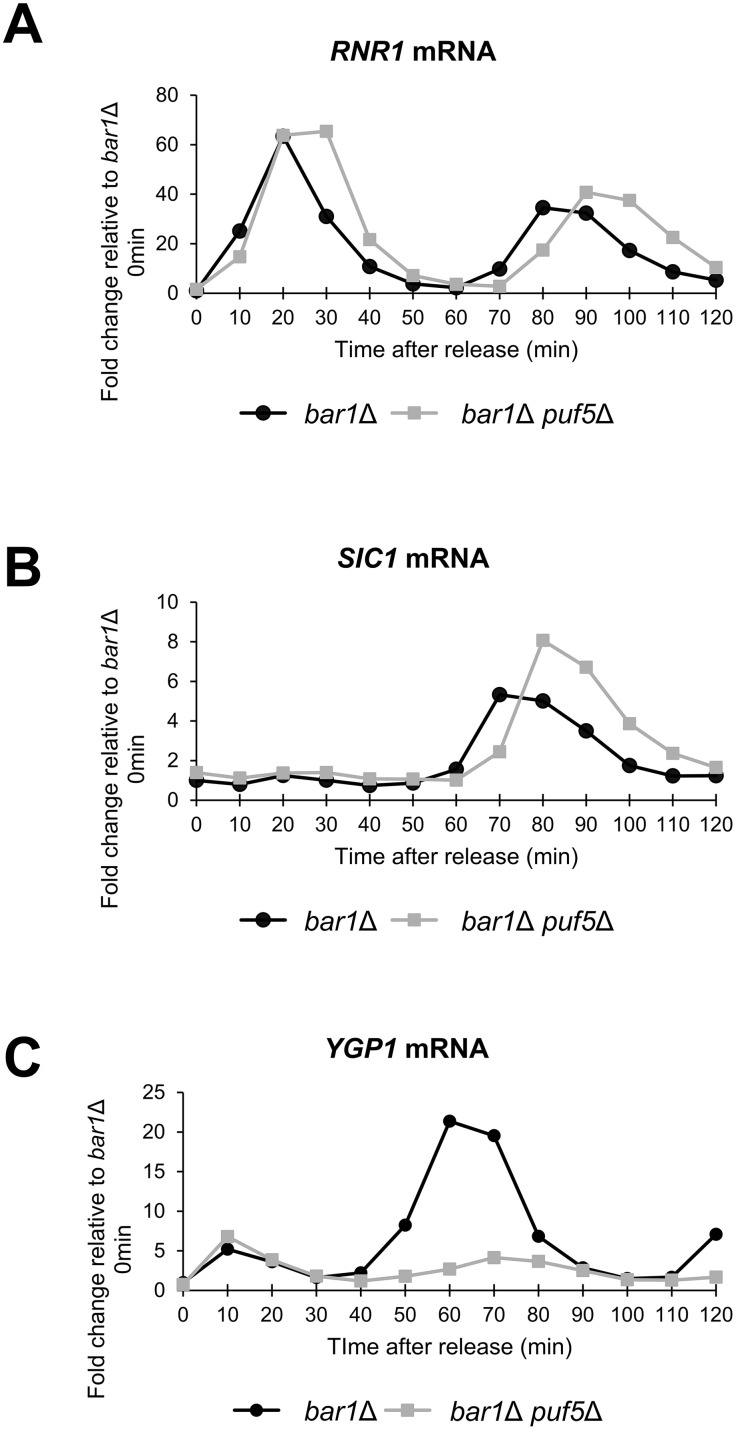
The rhythmic expression of *YGP1* in M-phase is induced by Puf5. The fold change of *RNR1* (A), *SIC1* (B), and *YGP1* (C) mRNA levels in the *bar1*Δ and *bar1*Δ *puf5*Δ mutants relative to the *bar1*Δ 0-min value at the indicated time points after release from G1-phase arrest. Analysis was performed using two biological replicates, each having three technical replicates. Data shown here are the representative Rq values from three technical replicates for one of the biological replicates. The results for the other biological replicate are shown in S1 Figs.

Previously, *YGP1* expression was reported to be regulated in a cell cycle-specific manner [16]: *YGP1* was allocated to the Mcm1-cluster gene which was induced from M to G1-phase during cell cycle. Given that Puf5 tunes cell cycle-specific expression of cyclin B genes *CLB1* and *CLB2* [31,41], we next examined whether the Puf5-mediated positive regulation is involved in the induction of cell cycle-specific *YGP1* expression. Cells were synchronized in the G1-phase using α-factor in a *bar1*Δ mutant background where the protease inactivating α-factor is absent. In the *bar1*Δ *puf5*Δ double mutant, the expression of the S-phase marker *RNR1* was induced upon similar timing to that in the *bar1*Δ mutant (Fig 3A and S1 Fig A). However, the peak expression of the late M-phase marker *SIC1* was slightly delayed in the double mutant, suggesting a G2/M-phase delay in the *bar1*Δ *puf5*Δ double mutant (Fig 3B and S1 Fig B). The expression of *YGP1* peaked after *RNR1* but before *SIC1* in the *bar1*Δ mutant, suggesting that *YGP1* is specifically expressed during the M-phase (Fig 3C and S1 Fig C). Notably, this *YGP1* induction was not observed in the *bar1*Δ *puf5*Δ double mutant (Fig 3C and S1 Fig C), indicating that Puf5 is required for the M-phase-specific induction of *YGP1* expression.

### A discrete promoter segment mediates Puf5‑dependent regulation of *YGP1*

**Fig 4:**
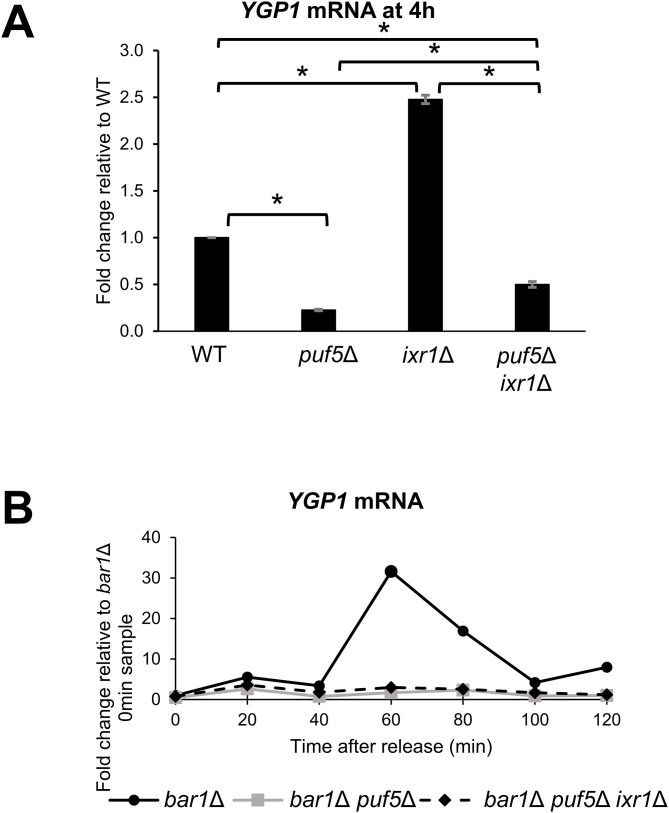
*YGP1* expression is regulated by Puf5 independently of Ixr1. (A) The fold change of *YGP1* mRNA levels in the *puf5*Δ, *ixr1*Δ, and *puf5*Δ *ixr1*Δ mutants relative to that in wild-type strain. The expression pattern was confirmed by two biological replicates, each having three technical replicates. The data show the average Rq values ± SEM (n = 3 technical replicates from one representative biological replicate). *p < 0.05 and **p < 0.01 are indicative of statistical significance. (B) The fold change of *YGP1* mRNA levels in the *bar1*Δ, *bar1*Δ *puf5*Δ, and *bar1*Δ *puf5*Δ *ixr1*Δ mutants relative to the *bar1*Δ 0-min value at the indicated time points after release from G1-phase arrest. Analysis was performed using two biological replicates, each having three technical replicates. Data shown here are the representative Rq values from three technical replicates for one of the biological replicates. The results for the other biological replicate are shown in S2 Fig.

Given that *YGP1* expression is regulated by Puf5 via its promoter (Figs 2D and E), we next aimed to clarify the detailed mechanism of this regulation. Since Puf5 is an RNA-binding protein which regulates its target mRNAs through binding to 3’UTR [32,33], we hypothesized that Puf5 indirectly controls *YGP1* promoter via regulating its transcriptional factor-coding mRNAs. We have previously clarified that Puf5 positively regulates the expression of *CLB1* and *CLB2* through the negative regulation of its target *IXR1* mRNA, which encodes a transcriptional repressor [31,41]. To assess whether the regulation of *YGP1* expression is also mediated by Ixr1, we measured the *YGP1* mRNA level in the wild-type, *puf5*Δ, *ixr1*Δ, and *puf5*Δ *ixr1*Δ mutants in an asynchronous culture. Interestingly, while the *ixr1*Δ mutant showed significantly higher *YGP1* mRNA levels than the wild-type (Fig 4A, WT vs *ixr1*∆), the *ixr1*Δ mutation only trivially restored the decreased *YGP1* expression caused by the *puf5*Δ mutation (Fig 4A, *puf5∆* vs *puf5∆ ixr1*∆). In addition, the *ixr1*Δ mutation also failed to restore the cell cycle-specific induction of *YGP1* in the *bar1*Δ *puf5*Δ background (Fig 4B and S2 Fig). These results suggest that Puf5 regulates *YGP1* expression independently of Ixr1, while Ixr1 negatively regulates *YGP1* expression.

**Fig 5:**
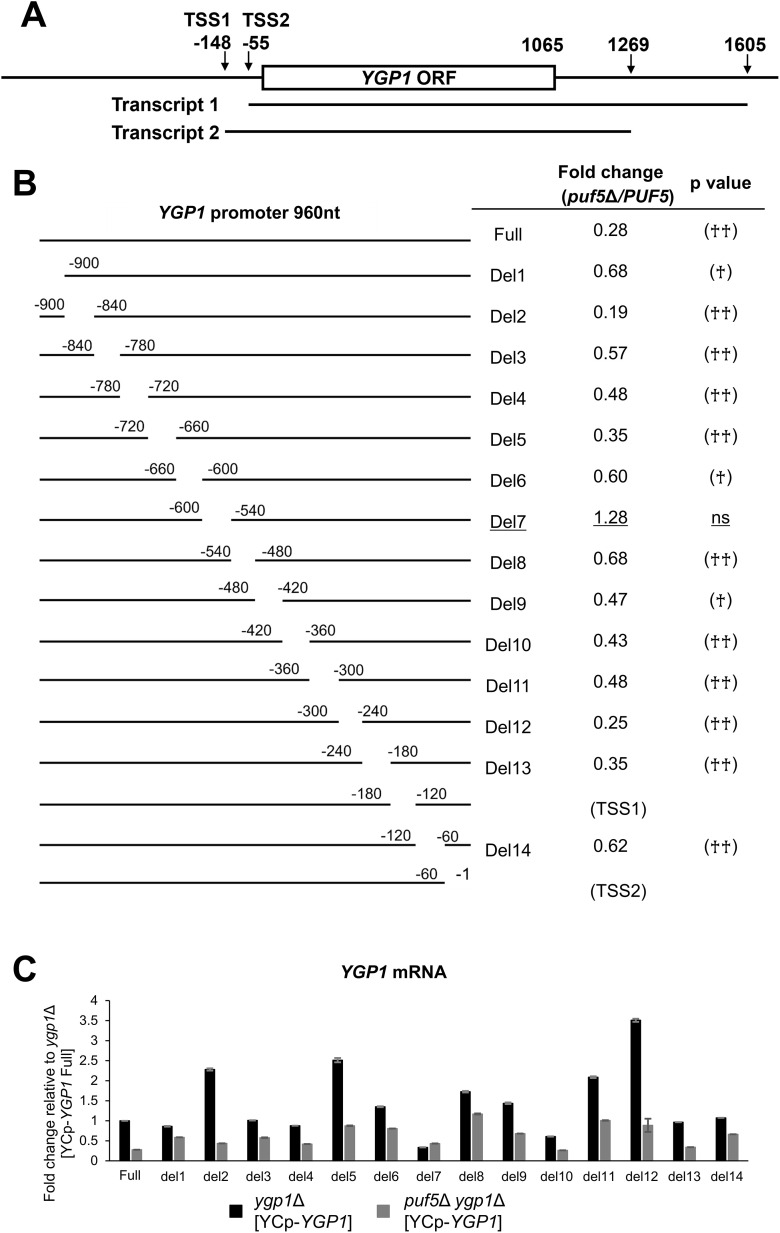
A discrete promoter segment mediates Puf5‑dependent regulation of *YGP1.* (A) A scheme of two *YGP1* transcripts. TSS1 corresponds to the transcription start site (TSS) of transcript 1, and TSS2 to the TSS of transcript 2. (B) The scheme of a series of *YGP1* promoter deletions. Every 60-nucleotide region was deleted from a YCplac33-*YGP1* plasmid except for the region containing transcriptional start sites (TSSs). After introducing a full-length YCplac33-*YGP1* plasmid or one of the deletion plasmids in the *ygp1*Δ and *ygp1*Δ *puf5*Δ strains, the strains were pre-cultured in SC-Ura medium followed by the 2-hour exposure to small amount of YPD and thereafter main culture in YPD medium until mid-log phase. The *YGP1* expression was quantified by RT-qPCR. The right score shows the fold change of the *YGP1* mRNA level in the *ygp1*Δ *puf5*Δ double mutant compared to that in the *ygp1*Δmutant (n = 3 technical replicates from one representative biological replicate), along with the corresponding *p*-values. (♰) *p* < 0.05 and (♰♰) *p* < 0.01 are indicative of statistical significance. ns, no statistical significance (*p* < 0.05). (C) The fold changes of *YGP1* in the *ygp1*Δ and *ygp1*Δ *puf5*Δ strains harboring a full-length YCplac33-*YGP1* plasmid or one of the deletion plasmids. The data show average Rq values ± SEM (n = 3 technical replicates from one representative biological replicate) relative to that in the *ygp1*Δ [YCplac33-*YGP1* Full] sample.

To clarify the regulatory mechanism by Puf5, we next aimed to map *YGP1* promoter elements that mediate the action of Puf5. We constructed a YCplac33-*YGP1* plasmid harboring a 960-nucleotide region upstream of the start codon as promoter, and generated a series of deletion constructs, each lacking a 60-nucleotide segment in the *YGP1* promoter. In this process, the regions including each transcriptional start site (TSS) of two *YGP1* transcripts were excluded (Fig 5A). We then introduced these plasmids into the *ygp1*Δ mutant and the *puf5*Δ *ygp1*Δ double mutant to assess the impact of each promoter region on exogenous *YGP1* mRNA levels (Fig 5B). The scores in Figure 5B show the fold changes of *YGP1* expression in the *puf5*Δ *ygp1*Δ double mutant relative to the *ygp1*Δ mutant, along with the corresponding *p*-values. The relative expression levels of *YGP1* in each strain were also manifested by a bar graph (Fig 5C). The expression of full-length *YGP1* was significantly decreased by the *puf5*Δ mutation (Figs 5B and C, Full). Interestingly, this decrease was canceled only in the del7 (−600 to −540) construct among 14 deletion constructs tested. These results indicate that the del7 region (−600 to −540) contains a critical segment for the Puf5-mediated control.

**Fig 6:**
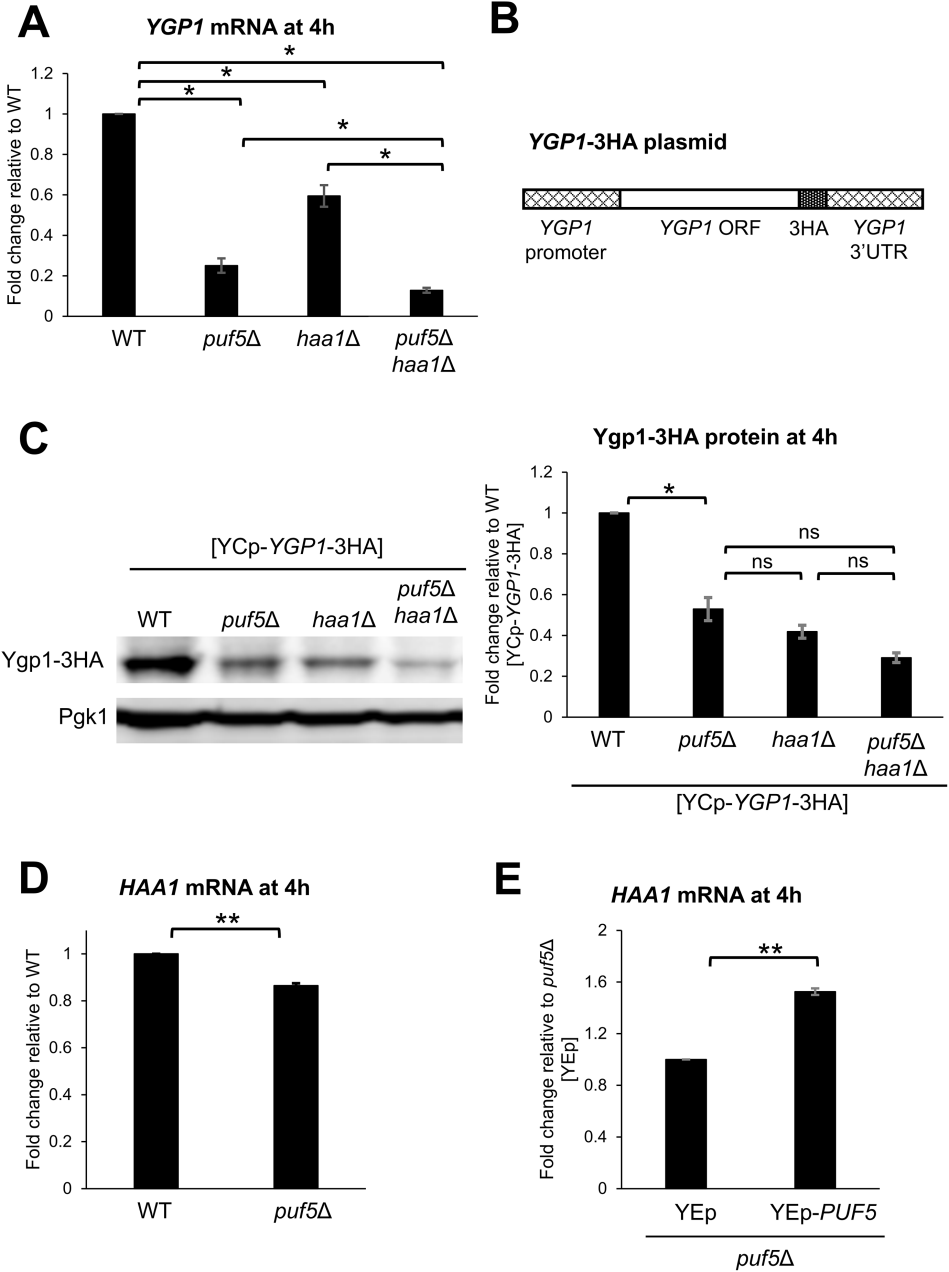
Transcriptional activator Haa1 positively regulates *YGP1* expression. (A) The fold changes of *YGP1* mRNA levels in the *puf5*Δ, *haa1*Δ, and *puf5*Δ *haa1*Δ mutants relative to that in wild-type strain. The expression pattern was confirmed by two biological replicates, each having three technical replicates. The data show the average Rq values ± SEM (n = 5 technical replicates from one representative biological replicate). *p < 0.05 is indicative of statistical significance. (B) A scheme of *YGP1*–3HA fragment on a YCplac33. (C) The Ygp1–3HA protein levels expressed from a YCplac33 plasmid in the wild-type, *puf5*Δ, *haa1*Δ, and *puf5*Δ *haa1*Δ mutants. The image shows Ygp1–3HA and Pgk1 (loading control). The graph shows the fold changes of Ygp1–3HA protein levels in the *puf5*Δ, *haa1*Δ, and *puf5*Δ *haa1*Δ mutants relative to that in wild-type strain. The expression pattern was confirmed by two biological replicates, each having three technical replicates. The data show the average fold change ± SEM (n = 6 technical replicates from one representative biological replicate). *p < 0.05 are indicative of statistical significance. ns, no statistical significance (*p* < 0.05). (D) The fold change of *HAA1* mRNA level in the *puf5*Δ mutant relative to that in wild-type strain. The expression pattern was confirmed by three biological replicates, each having three technical replicates. The data show the average Rq values ± SEM (n = 4 technical replicates). **p < 0.01 is indicative of statistical significance. (E) The fold change of *HAA1* mRNA level in the *puf5*Δ [YEplac195-*PUF5*] strain relative to that in *puf5*Δ [YEplac195 vector]. The expression pattern was confirmed by two biological replicates, each having three technical replicates. The data show the average Rq values ± SEM (n = 3 technical replicates from one representative biological replicate). **p < 0.01 is indicative of statistical significance.

To identify the responsible factor which directly acts on the del7 region (−600 to −540), we searched YEASTRACT+ database, which catalogs transcriptional regulatory interactions in yeast (https://yeastract.com/). Analysis of the 60-nucleotide region (−600 to −540) revealed several transcription factors with binding motifs within this segment (Table 2). Comparing these factors with those having binding evidence to the *YGP1* promoter (Table 3), we focused on the transcription activator Haa1. Haa1 is known to mediate the weak acid stress responses [20,21] through regulating the expression of 80% of acid stress-responsive genes [42]. Haa1 is also reported to localize the nucleus and positively regulate *YGP1* expression [20,22]. To confirm the role of Haa1 in *YGP1* expression, we investigated the *YGP1* mRNA level in the wild-type, *puf5*Δ, *haa1*Δ, and *puf5*Δ *haa1*Δmutants. *YGP1* expression was approximately 40% lower in the *haa1*Δ mutant than in the wild-type and notably showed further decrease in the *puf5*Δ *haa1*Δ double mutant (Fig 6A), indicating that Puf5 regulates *YGP1* expression independently of Haa1. Then we next examined the Ygp1 protein levels using the exogenously expressed *YGP1*–3HA from a YCplac33 plasmid (Fig 6B). In this experiment, to minimize differences in culture conditions, the strains were pre-cultured in SC-Ura medium, followed by main culture in YPD medium. Even when main culture was performed in YPD medium, the plasmid carriage rate for each strain exceeded 95%, and no significant differences were observed between strains (Table 4). The Ygp1–3HA protein level was also significantly decreased in the *puf5*Δ and *haa1*Δ mutants compared to the wild-type (Fig 6C). In the *puf5*Δ *haa1*Δ double mutant, although the difference was not significant, the protein level was lower than the *puf5*Δ or *haa1*Δ single mutant (Fig 6C). While these data suggest that Puf5 functions independently of Haa1, the binding evidence of Puf5 to *HAA1* mRNA in the previous genome-wide mapping of Puf5-mRNA interaction [43] implied the possibility that Haa1 acts downstream of Puf5. Indeed, *HAA1* mRNA levels was ~ 20% decreased in the *puf5*Δ mutant (Fig 6D) and ~50% increased by the multicopy-*PUF5* (Fig 6E). Taken together, it is suggested that Puf5 and Haa1 positively regulate the *YGP1* expression through the 60-nucleotide promoter region (−600 to −540) and that the Puf5-mediated regulation is partly mediated by Haa1.

**Table 2:** Transcriptional factors harboring binding motif within del7 region.

Transcriptional Factor	Binding motif	Position
Gcr1	CWTCC	−588
Hap1	CGGN{6}CGG	−582
Mal63, Znf1	CGGN{9}CGG	−582
Oaf1	GTCATCCG/ CCGCGGAA	−590/ −585
Pdr1, Pdr3, Ert1	TCCGCGGA	−586
Pdr8	TCCGHGGA	−586
Rgt1	CGGANNA	−582
Rtg3	GTCATC	−590
Stb5	CGGNS	−573/ −570
Yrr1	WCCGYKKWW	−586
Gsm1	CGGNNNNNNNNNCGG	−582
Haa1	SMGGSG	−574

**Table 3:** Transcriptional factors with binding evidence to *YGP1* promoter.

Transcriptional Factors		
Cin5p	Msn2p	Tec1p
Ecm22p	Msn4p	Yap1p
Fkh1p	Pho4p	Hap1p
Fkh2p	Rsf2p	Ndt80p
Haa1p	Sok2p	Met32p
Hot1p	Spt23p	Sut1p
Ino2p	Ste12p	Tye7p
Mcm1p		

**Table 4:** Plasmid carriage rates in yeast strains harboring the YCplac33-*YGP1*-3HA plasmid after 24 hours of incubation in non-selective medium.

Strains harboring YCplac33-*YGP1*–3HA plasmid	Carriage rate (%)	
rep1	rep2
Wild-type	97.2	99.4
*puf5*Δ	98.6	99.2
*haa1*Δ	98.1	99.4
*puf5*Δ *haa1*Δ	99.0	98.2
**Statistical significance**	n.s. (*p* = 0.929)	

Yeast strains harboring a YCplac33-*YGP1*–3HA plasmid were pre-cultured in SC-Ura medium and then transferred to YPD medium for the main culture. After 24 h of incubation, cells were diluted 10,000-fold with water and spread onto YPD plates. After incubation at 25 °C for 2 days, the resulting colonies were replica-plated onto SC-Ura plates to assess plasmid carriage rates. The table shows the calculated results from two biological replicates. Bartlett’s test confirmed homogeneity of variances (*p* = 0.76). A one-way ANOVA showed no significant differences among groups (*F*(3, 4) = 0.143, *p* = 0.929).

### Haa1 partially contributes to the periodic expression of *YGP1* during the cell cycle

**Fig 7:**
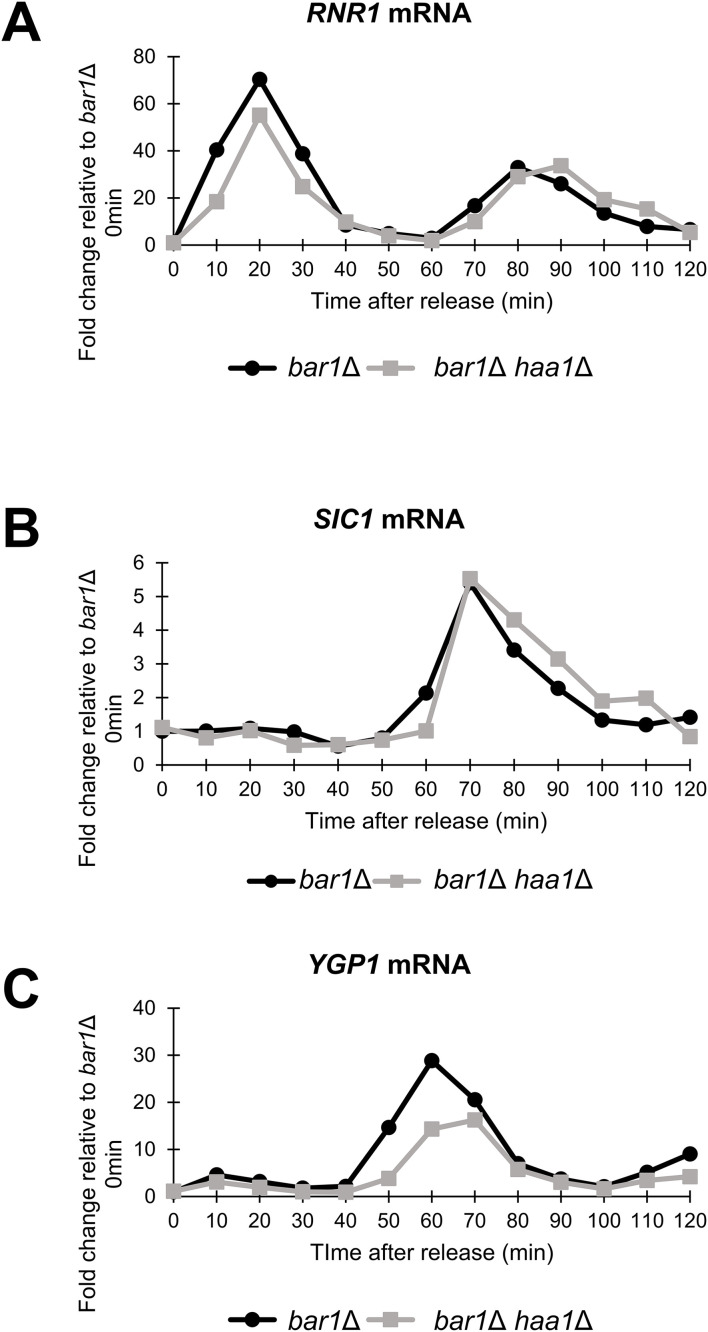
Haa1 partly regulates the expression of *YGP1* during the cell cycle. The fold change of *RNR1* (A), *SIC1* (B), and *YGP1* (C) mRNA levels in the *bar1*Δ and *bar1*Δ *haa1*Δ mutants relative to the *bar1*Δ 0-min value at the indicated time points after release from G1-phase arrest. Analysis was performed using two biological replicates each having three technical replicates. Data shown here are the representative Rq values from three technical replicates for one of the biological replicates. The results for the other biological replicate are shown in S3 Figs.

To test the role of Haa1 in the M-phase-specific induction of *YGP1* expression, we synchronized the *bar1*Δ *haa1*Δ double mutant and examined *YGP1* mRNA level. In the *bar1*Δ *haa1*Δ double mutant, the expression of the S-phase marker *RNR1* and the late M-phase marker *SIC1* peaked at similar time points to those observed in the *bar1*Δ mutant (Figs 7A and B, and S3 Figs A and B), suggesting that Haa1 has no major effect on cell cycle progression. While *YGP1* induction was observed in the *bar1*Δ *haa1*Δ double mutant, the peak appeared to be modestly reduced and slightly delayed compared with that in the *bar1*Δ mutant (Fig 7C and S3 Fig C). Together with the results from asynchronous culture (Figs 6A and C), it is suggested that Haa1 partially contributes the induction of the M‑phase-specific induction of *YGP1* expression.

### *YGP1* expression is highly induced at the diauxic shift

**Fig 8:**
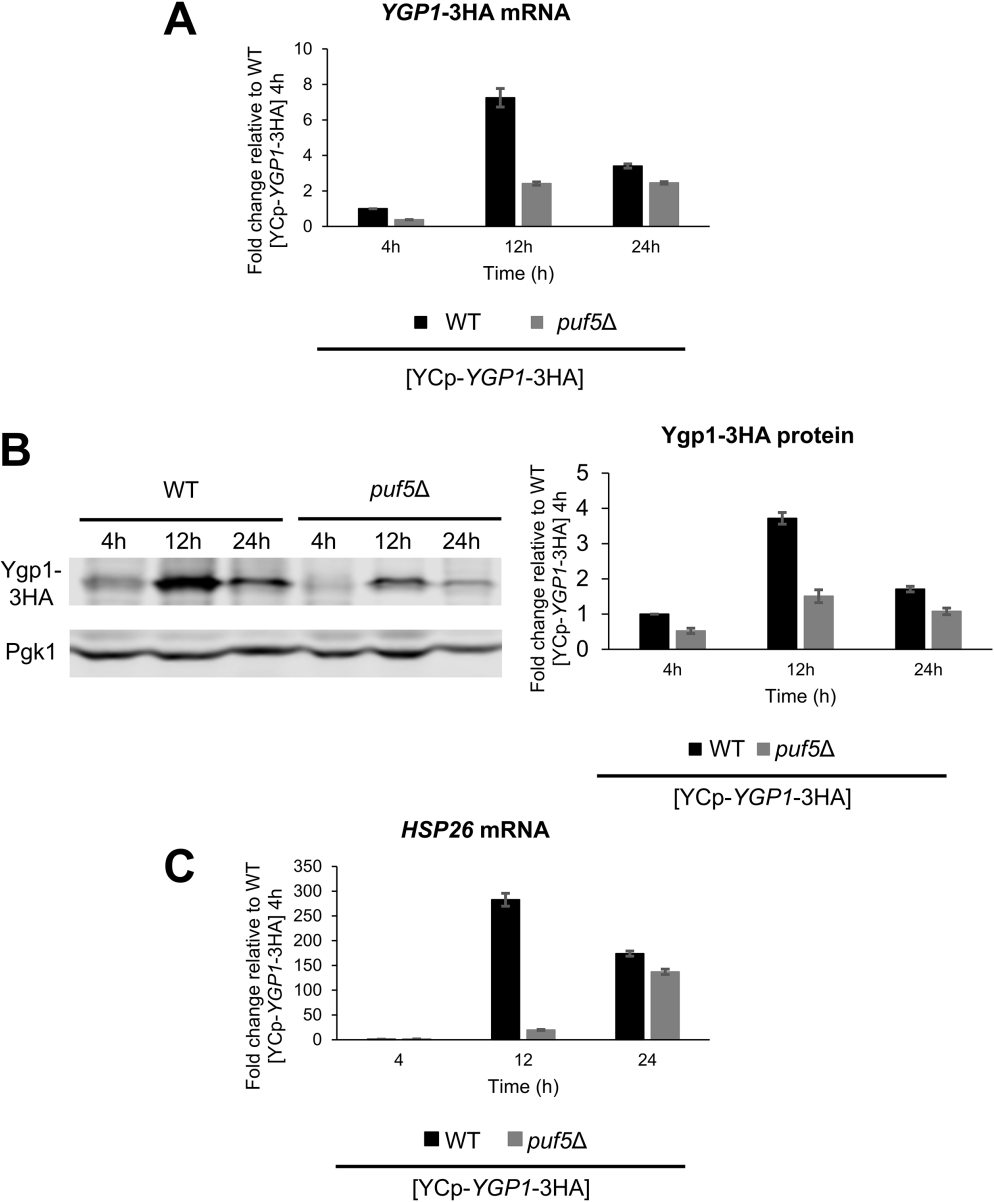
*YGP1* expression is induced at the diauxic shift. (A) The fold changes of *YGP1*–3HA mRNA expressed from a YCplac33 plasmid in the wild-type and *puf5*Δ mutant along a time course relative to the wild-type 4 hours value. The expression pattern was confirmed by two biological replicates, each having three technical replicates. The data show the average Rq values ± SEM (n = 3 technical replicates from one representative biological replicate). (B) The Ygp1–3HA protein levels expressed from a YCplac33 plasmid in the wild-type and *puf5*Δ mutant. The image shows Ygp1–3HA and Pgk1 (loading control). The expression pattern was confirmed by two biological replicates, each having three technical replicates. The graph shows the mean ± SEM of the fold changes of Ygp1–3HA protein levels relative to that in wild-type 4 hours (n = 4 technical replicates from one representative biological replicate). (C) The fold change of *HSP26* mRNA levels in the wild-type and *puf5*Δ mutant harboring a Ycplac33 *YGP1*–3HA plasmid along a time course relative to the wild-type 4 hours value. The expression pattern was confirmed by two biological replicates, each having three technical replicates. The data show the average Rq values ± SEM (n = 3 technical replicates from one representative biological replicate).

Given that *YGP1* expression is regulated by two distinct mechanisms depending on glucose availability, we next examined the regulatory mechanism governing *YGP1* expression at the diauxic shift. *YGP1* expression is strongly induced at the time of the diauxic shift (Figs 1A–C). To assess whether these expression patterns are reflected in those of Ygp1 protein levels, we exogenously expressed *YGP1*–3HA from a YCplac33 plasmid (Fig 6B). Since the plasmid retention rates remained above 95% after 24 h of cultivation in non-selective YPD medium (Table 4), main cultures were performed in YPD medium. *YGP1*–3HA mRNA levels, like endogenous *YGP1* mRNA levels, rose at 12 hours and fell by 24 hours in wild-type (Fig 8A). Ygp1–3HA protein levels also showed a similar pattern (Fig 8B), indicating the diauxic shift-responsive induction of *YGP1* is reflected to the protein level. Surprisingly, in this experiment, both *YGP1* mRNA and protein levels at 12 hours were attenuated in the *puf5*Δ mutant compared to those in the wild-type, while the relative induction at 12 hours (12h/4h) was maintained (Fig 8A and B). The reason for the difference in expression patterns between endogenous *YGP1* and *YGP1*–3HA was unclear. However, considering that the plasmid-carrying strain was pre-cultured in SC-Ura medium before the main culture in YPD medium, the impaired response to the diauxic shift in the *puf5*Δ mutant may be caused by the prior exposure to SC medium. Indeed, the stationary phase marker *HSP26* induction level at 12 hours was also reduced in the *puf5*Δ mutant harboring a *YGP1*–3HA plasmid (Fig 8C).

We next examined the potential involvement of Haa1 in the *YGP1* induction at the diauxic shift. Time-course analysis revealed that endogenous *YGP1* expression in the *haa1*Δ mutant was comparable to that in the wild-type (S4 Fig A), indicating Haa1 is not involved in this induction of *YGP1* at the diauxic shift. Interestingly, when examined *YGP1*–3HA mRNA and Ygp1–3HA protein levels expressed from a plasmid, both mRNA and protein levels were slightly decreased in the *haa1*Δ mutant (S4 Figs B and C). In this experiment as well, since plasmid retention exceeded 95%, with no significant differences among strains (Table 4), the main cultures were performed in YPD medium. In addition, the expression of *HSP26*, a stationary phase marker, was mildly decreased in the *haa1*Δ mutant harboring a *YGP1*–3HA plasmid at 12h, while it was decreased more markedly in the *puf5*Δ mutant (S4 Fig D). These results suggest that Puf5 can function independently of Haa1, but the machinery involving Puf5 and Haa1 appears to positively regulate the response to the diauxic shift including *YGP1* induction at least in the plasmid-carrying strains used in these experiments.

### Transcriptional factors Msn2/Msn4 are responsible for the induction of *YGP1* expression at the diauxic shift

**Fig 9:**
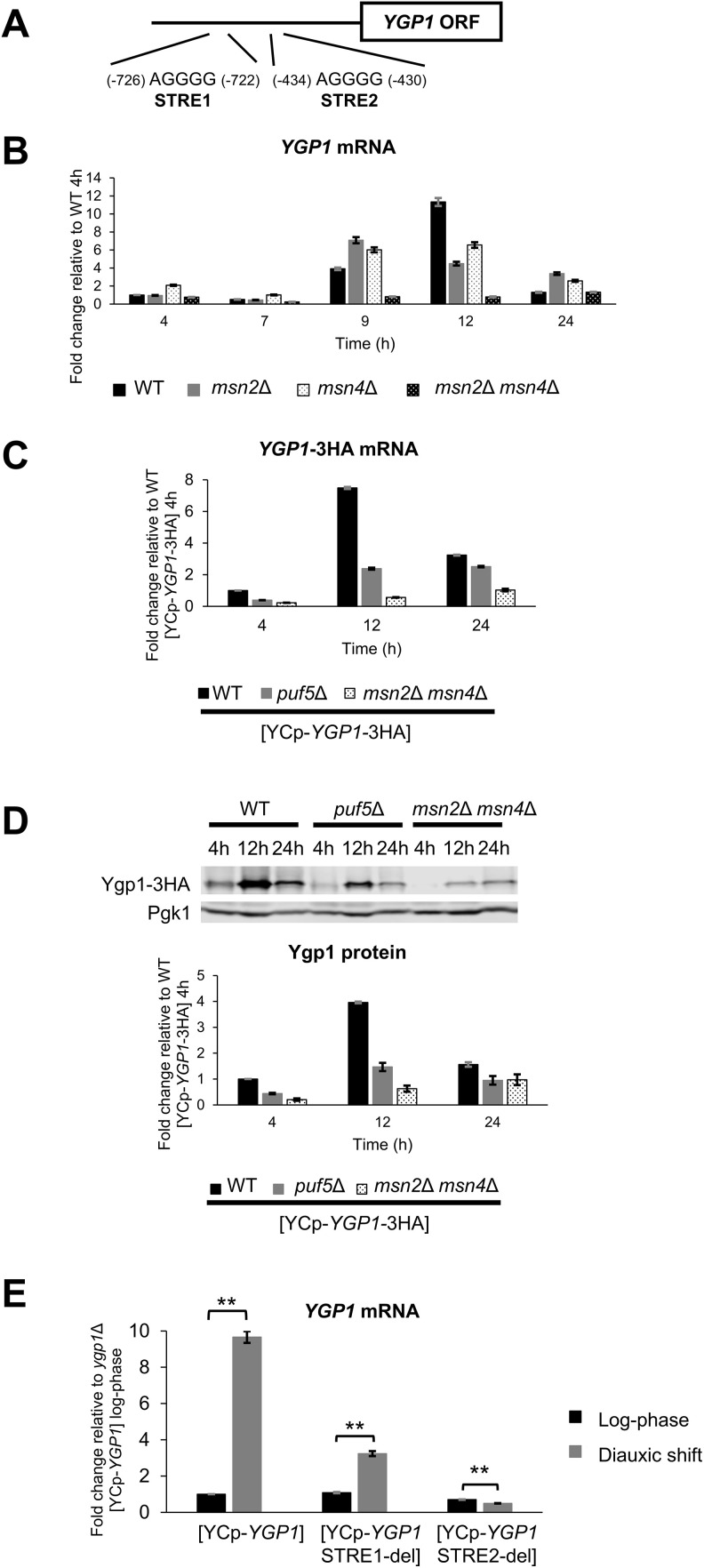
The induction of *YGP1* expression is triggered by the transcriptional activators Msn2 and Msn4. (A) A scheme of *YGP1* promoter. Two stress response elements (STREs) exist within *YGP1* promoter. The upstream one is labeled as STRE1, and the downstream one is as STRE2. (B) The fold change of *YGP1* mRNA levels in the wild-type, *msn2*Δ, *msn4*Δ, and *msn2*Δ *msn4*Δ mutants along a time course relative to the wild-type 4-hours value. The data show the average Rq values ± SEM (n = 3 technical replicates from one representative biological replicate). (C) The fold change of *YGP1*–3HA mRNA levels expressed from a YCplac33 plasmid in the wild-type, *puf5*Δ, and *msn2*Δ *msn4*Δ mutants along a time course relative to the wild-type 4-hours value. The expression pattern was confirmed by two biological replicates, each having three technical replicates. The data show the average Rq values ± SEM (n = 3 technical replicates from one representative biological replicate). (D) The Ygp1–3HA protein levels expressed from a YCplac33 plasmid in the wild-type, *puf5*Δ, and *msn2*Δ *msn4*Δ mutants. The image shows Ygp1–3HA and Pgk1 (loading control). The expression pattern was confirmed by two biological replicates, each having three technical replicates. The graph shows the average fold change ± SEM of the fold changes of Ygp1–3HA protein levels relative to that in wild-type 4 hours (n = 4 technical replicates from one representative biological replicate). (E) The fold change of *YGP1* mRNA levels in the *ygp1*Δ mutant harboring a YCplac33-*YGP1*, YCplac33-*YGP1* STRE1-del, or YCplac33-*YGP1* STRE2-del plasmid at the logarithmic growth phase or the diauxic shift. The expression pattern was confirmed by three biological replicates, each having three technical replicates. The data show the average Rq values ± SEM of the fold change relative to that in the logarithmic growth phase sample of *ygp1*Δ [YCplac33-*YGP1*] (n = 3 technical replicates from one representative biological replicate). Post-hoc pairwise comparisons were conducted using the aligned rank transform (ART) procedure followed by Holm-adjusted *p*-values to control the family-wise error rate.

To specify the responsible factor which induces *YGP1* expression at the diauxic shift, we employed *YGP1* promoter-deletion constructs again and measured mRNA levels at mid-log phase and the diauxic shift. Since no apparent plasmid loss was observed in both *ygp1*Δ and *puf5*Δ *ygp1*Δ strains after 24 h in non-selective YPD medium (Table 5), strains were pre-cultured in SC-Ura medium followed by the main culture in YPD medium. In S5 Fig A, relative fold change of *YGP1* mRNA level (diauxic shift/ log phase) and the respective *p*-values were listed. In the wild-type strain, *YGP1* expression was induced approximately 10 times at the diauxic shift (S5 Figs A and B, Full). This induction level was reduced in all deletion constructs but was particularly decreased to less than 1.5 times in the deletion constructs 7–11 (S5 Figs A and B). Table 6 lists transcription factors with predicted binding motifs within the regions (−600 to −300) corresponding to the deletion constructs 7–11. Among them, we focused on the stress-responsive transcription factors, Msn2 and Msn4, which harbor their binding element in the del 9 region (−480 to −420). The transcription factors Msn2 and Msn4 play central roles in regulating the general stress responses. Upon exposure to nutrient deprivation, organic acids, or heat shock, they translocate to the nucleus and activate transcription through binding to STREs in target gene promoters [7–10]. They are recognized as major regulators of the environmental stress response (ESR) [11] and are particularly responsive to glucose starvation, which induces their rapid nuclear accumulation [10,12,13]. The *YGP1* promoter contains two STREs, STRE1 (upstream, −729 to −722) and STRE2 (downstream, −434 to −430) (Fig 9A), suggesting that Msn2 and Msn4 may regulate *YGP1* under glucose starvation. To test this hypothesis, we measured endogenous *YGP1* mRNA levels in the wild-type, *msn2*Δ, *msn4*Δ, and *msn2*Δ *msn4*Δ mutants over time. The *YGP1* mRNA level at 12 hours was reduced in the *msn2*Δ and *msn4*Δ mutants and nearly abolished in the *msn2*Δ *msn4*Δ double mutant (Fig 9B). In this experiment, *YGP1* expression started to increase from 9h (Fig 9B), exhibiting a slightly distinct pattern from that shown in Fig 1A, where expression levels remained relatively low at this time point. Nevertheless, the peak expression of *YGP1* expression in wild-type cells was consistently detected at 12 h. As shown in Fig 1C, glucose levels at 9 h were close to depletion, suggesting that subtle differences in glucose concentrations during medium preparation may have caused small variations in the timing of glucose exhaustion. The *YGP1*–3HA mRNA and Ygp1–3HA protein levels expressed from a YCplac33 plasmids also showed similar expression patterns in the wild-type and *msn2*Δ *msn4*Δ double mutant (Figs 9C and D). Consistent with Fig 8A and 8B, the *puf5*Δ mutant showed an overall decrease in the *YGP1*–3HA mRNA and Ygp1–3HA protein levels (Figs 9C and D). In this experiment, we also confirmed that the *msn2*Δ *msn4*Δ double mutant exhibited the comparable plasmid carriage rate to that observed in wild-type and *puf5*Δ strains (Table 7). Deletion analysis of the STREs on a YCplac33-*YGP1* plasmid showed partial loss of induction with STRE1 deletion and complete loss with STRE2 deletion, while the basal expression was retained in these deletions (Fig 9E). We conclude that Msn2 and Msn4 are the principal drivers for *YGP1* expression by glucose-starvation, acting primarily through STRE2 (−434 to −430).

**Table 5:** Plasmid carriage rates in yeast strains harboring the YCplac33-*YGP1* plasmid after 24 hours of incubation in non-selective medium.

Strains harboring YCplac33-*YGP1* plasmid	Carriage rate (%)	
	rep1	rep2
*ygp1*Δ	98.8	98.5
*puf5*Δ *ygp1*Δ	98.6	98.0
**Statistical significance**	n.s. (*p* = 0.459)	

Yeast strains harboring a YCplac33-*YGP1* plasmid were pre-cultured in SC-Ura medium, transferred to YPD medium, and incubated for 24 h. Cells were then diluted 10,000-fold, plated on YPD, and incubated at 25 °C for 2 days. Colonies were replica-plated onto SC-Ura plates to determine plasmid carriage rates. The table shows the calculated results from two biological replicates. An F-test indicated no significant difference in variances between the two groups (*p* = 0.586). Accordingly, Welch’s *t*-test showed no significant difference (*t*(1.46) = 0.986, *p* = 0.459).

**Table 6:** Transcriptional factors harboring binding motif within each deletion region.

Deletion	Transcriptional factors
del7	Gcr1, Hap1, Mal63, Znf1, Oaf1, Pdr1, Pdr3, Pdr8, Ert1, Rgt1, Rgt3, Stb5, Yrr1, Gsm1, Haa1, Ert1
del8	Crz1, Mcm1, Stb5, Tye7
del9	Cat8, Hsf1, Skn7, Xbp1, Gis1, Msn2, Msn4, Rph1, Com2, Usv1, Sut1
del10	Crz1, Mcm1
del11	Cat8, Pip2, Stb5, Mot2, Ert1

**Table 7:** Plasmid carriage rates in yeast strains harboring the YCplac33-*YGP1*-3HA plasmid after 24 hours of incubation in non-selective medium.

Strains harboring YCplac33-*YGP1*–3HA plasmid	Carriage rate (%)	
	rep1	rep2
Wild-type	97.2	99.4
*puf5*Δ	98.6	99.2
*msn2*Δ *msn4*Δ	98.9	98.2
**Statistical significance**	n.s. (*p* = 0.8345)	

Yeast strains harboring a YCplac33-*YGP1*–3HA plasmid were pre-cultured in SC-Ura medium and then transferred to YPD medium for the main culture. After 24 h of incubation, cells were diluted 10,000-fold with water and spread onto YPD plates. After incubation at 25 °C for 2 days, the resulting colonies were replica-plated onto SC-Ura plates to assess plasmid carriage rates. The table shows the calculated results from two independent experiments. As this experiment was conducted in parallel with Table 4, the wild-type and *puf5*Δ data shown in Table 4 were used. Bartlett’s test confirmed homogeneity of variances (*p* = 0.51). A one-way ANOVA showed no significant differences among groups (*F*(2,3) = 0.1923, *p* = 0.8345).

### Ygp1 maintains long-term cell survival under the short-lived *puf5*Δ background

**Fig 10:**
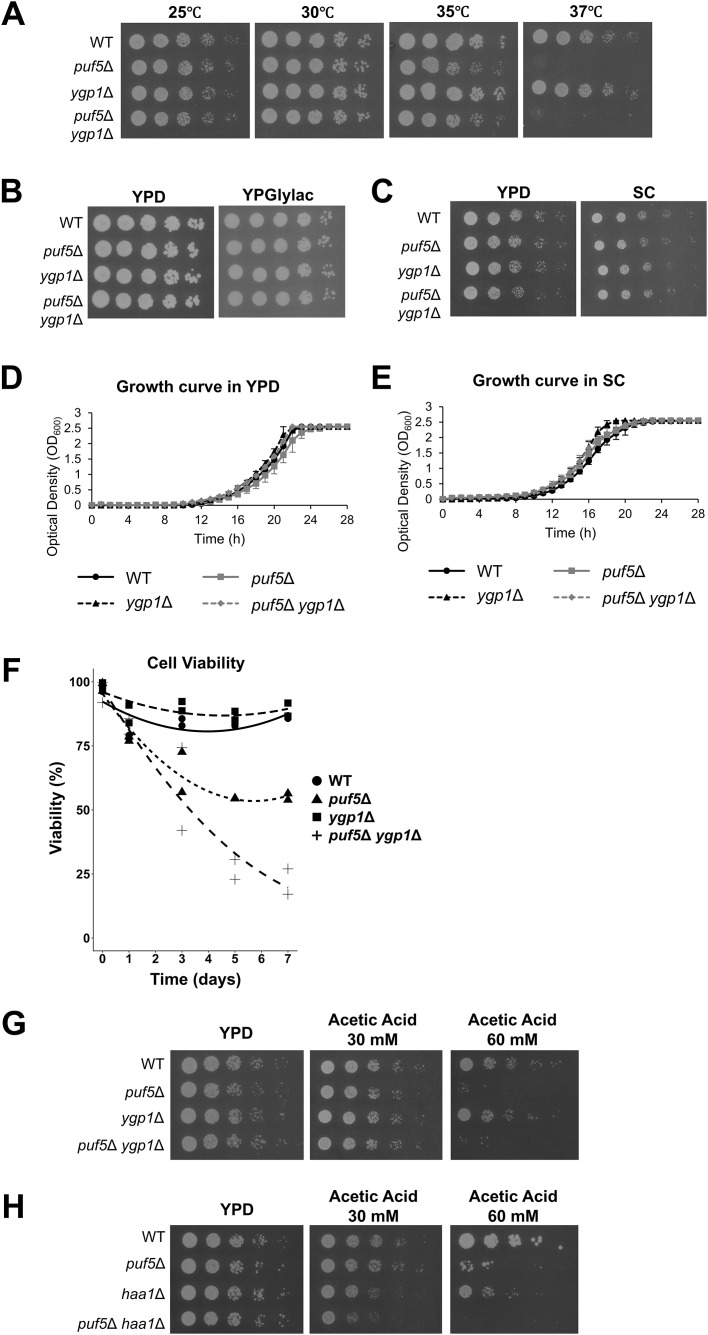
*YGP1* regulation mediated by Puf5 maintains long-term cell survival. (A) Spot assay data of the wild-type, *puf5*Δ, *ygp1*Δ, and *puf5*Δ *ygp1*Δ mutants. The strains were cultured until mid-log phase, serially diluted, and spotted on YPD plates. The plates were incubated at an appropriate temperature. The representative images are shown. (B, C) Spot assay data of the wild-type, *puf5*Δ, *ygp1*Δ, and *puf5*Δ *ygp1*Δ mutants. The strains were cultured until mid-log phase, serially diluted, and spotted on YPD plates, YPGlylac plates (B), or SC plates (C). The plates were incubated at 25 degrees. The representative images are shown. (D, E) Growth curve of the wild-type, *puf5*Δ, *ygp1*Δ, and *puf5*Δ *ygp1*Δ mutants in YPD (D) or SC medium (E). The strains were cultured overnight and transferred in fresh medium to final OD_600_ < 0.001. Optical density (OD_600_) was measured every 1 hour. The data show the mean ± SE (n = 2 biological replicates) of the measured OD_600_. (F) Cell viability during long-term culture in the wild-type, *puf5*Δ, *ygp1*Δ, and *puf5*Δ *ygp1*Δ mutants. The strains were cultured until mid-log phase (day 0) and continuously harvested until day 7 in SC medium. Cell viability was measured at each time point. A two-dimensional regression analysis was performed on the measurements (n = 2 biological replicates), and both the observed data and the fitted regression curve are presented. The graph was generated using the ggplot2 package in R [45,46]. (G) Spot assay data of the wild-type, *puf5*Δ, *ygp1*Δ, and *puf5*Δ *ygp1*Δ mutants. The strains were cultured until mid-log phase, serially diluted, and spotted on YPD plates, YPD-Acetic Acid 30 mM plates, and YPD-Acetic Acid 60 mM plates. The plates were incubated at 25 degrees. The representative images are shown. (H) Spot assay data of the wild-type, *puf5*Δ, *haa1*Δ, and *puf5*Δ *haa1*Δ mutants. The strains were cultured until mid-log phase, serially diluted, and spotted on YPD plates, YPD-Acetic Acid 30 mM plates, and YPD-Acetic Acid 60 mM plates. The plates were incubated at 25 degrees. The representative images are shown.

We next examined the physiological importance of the regulation of *YGP1* expression by Puf5. The *ygp1*Δ mutant did not exhibit growth retardation or genetic interaction with the *puf5*Δ mutant under optimal conditions (YPD medium at 25–30 °C) (Fig 10A, 25 °C and 30 °C), at elevated temperature (Fig 10A, 35 °C and 37 °C) or non-fermentative condition (YPGlylac medium) (Fig 10B).

Since we demonstrated that the Puf5-mediated regulation involving Haa1 contributes to the diauxic shift in the strain harboring a plasmid pre-cultured in SC medium (Figs 8A–C and S4 Figs A–C), we next examined cell growth or survival rate in SC medium. The *puf5*Δ, *ygp1*Δ, and *puf5*Δ *ygp1*Δ mutants grew similarly to the wild type in SC medium (Figs 10C–E). However, the long-term survival rate was decreased in the *puf5*Δ mutant and further decreased in the *puf5*Δ *ygp1*Δ double mutant compared to that in the *puf5*Δ single mutant (Fig 10F). Therefore, Ygp1 is suggested to support long-term cell survival in the *puf5*Δ background. Previously, it was reported that cell survival rate decreased during long-term culture in SC medium due to glucose depletion and rapid pH decline by the accumulation of the metabolic byproduct acetic acid [44]. Because of its lower buffering capacity, SC medium is known to exhibit a more dynamic pH decrease than YPD medium. Upon revision of the stationary-phase marker *HSP26* during long-term culture in YPD medium, we found that *HSP26* expression during the stationary phase (days 1–6) were lower in the *puf5*Δ mutant than in wild-type cells, while its induction at the diauxic shift was similar between the two strains (Fig 1B). Therefore, we hypothesized that Puf5 contributes to the acid stress responses and maintains long-term cell survival, with Ygp1 playing a positive role in this process in the *puf5*Δ mutant strain. Indeed, it was reported that Haa1-mediated *YGP1* regulation is involved in the acid stress responses [21], and that *YGP1* expression is induced by lactic acid, acetic acid, and hydrochloric acid stresses [23]. To confirm our hypothesis, we next investigated the sensitivity to acetic acid. Contrary to a previous report [21], the *ygp1*Δ mutant did not show sensitivity to acetic acid (Fig 10G). As expected, the *puf5*Δ mutant exhibited the sensitivity to acetic acid; however, the *ygp1*Δ deletion did not further enhance the acetic acid sensitivity of the *puf5*Δ mutant (Fig 10G). These observations suggest that Ygp1 is not a major determinant of acetic acid sensitivity on YPD plates containing acetic acid. In contrast, the *haa1*Δ deletion resulted in clear sensitivity to acetic acid. Moreover, the *puf5*Δ *haa1*Δ double mutant displayed more severe sensitivity than either the *puf5*Δ or *haa1*Δ single mutant (Fig 10H). Together, these results indicate that the Puf5-mediated regulation involving Haa1 contributes to the cellular response to acetic acid stress, while the specific contribution of Ygp1 as a downstream target remains to be determined.

## Discussion

### *YGP1* expression is regulated by Puf5 through its promoter mediated by Haa1

In this study, we investigated the regulation machinery of nutrition deprivation-responsive gene *YGP1* (Fig 1A) and clarified that *YGP1* expression is positively regulated by an RNA-binding protein Puf5 in the logarithmic growth phase (Figs 2A and B). This positive regulation also contributed to the cell cycle-dependent expression of *YGP1* at the M-phase (Fig 3C and S1 Fig C). While the *YGP1* promoter-*GFP* reporter assay revealed that Puf5 affects the *YGP1* promoter (Figs 2D and E), the effect of the *puf5*Δ mutation on the reporter expression was smaller than that on the endogenous expression (Figs 2A–E). However, *GFP-YGP1* 3’UTR reporter expression was not significantly changed in the *puf5*Δ mutant (Fig 2G), suggesting that Puf5 specifically influences the *YGP1* promoter. In the reporter construction, we fused *ADH1* 3’UTR to the *YGP1*promoter-*GFP* gene (Fig 2C), which is reported to be 1.3-times more stable than *YGP1* 3’UTR [47]. Therefore, it is possible that the promoter function was masked by the stabilization of reporter mRNA. Indeed, although the difference was not significant, replacing the *YGP1* 3’UTR with *ADH1* 3’UTR increased *GFP* reporter expression (Fig 2G). Nevertheless, the promoter deletion assay revealed the specific 60-nucleotide promoter region where Puf5 exerts its effect (Figs 5B and C). Therefore, we assume that the *YGP1* promoter is the main responsible region for the regulation.

The positive regulation of *YGP1* expression by Puf5 was independent of Ixr1 (Figs 4A and B, and S2 Fig). This result contrasts with previously reported Puf5-mediated *CLB1*/*CLB2* regulation, which is dependent on Ixr1 [31,41]. From the promoter assay results, we specified a potential downstream factor Haa1, a transcriptional activator involved in weak acid responses [20–22] which regulates the expression of 80% of acid stress-responsive genes [42]. Haa1 positively regulates *YGP1* expression during the logarithmic growth phase (Figs 6A and C) and also weakly affects M-phase specific induction (Fig 7C and S3 Fig C). Puf5 binds to *HAA1* mRNA [43] and positively regulates its expression (Figs 6D and E), indicating that Haa1 functions downstream of Puf5. However, it remains unclear how Puf5 positively regulates *HAA1* expression. Since it has been reported that Puf5 mainly functions as a negative regulator of its target mRNAs [32,33], our findings raise the possibility that the regulation of *HAA1* expression by Puf5 may involve not only direct binding to *HAA1* mRNA but also indirect mechanisms, such as regulation of transcription factors that regulate *HAA1* expression.

In addition, Puf5 and Haa1 were suggested to regulate *YGP1* expression both dependently and independently. Then, we questioned what another factor is functioning downstream of Puf5. Among transcriptional factors which have their own binding motif within 60-nucleotide promoter region (−600 to −540 from start codon) (Table 2), Rgt1, Stb5, and Yrr1 are factors whose mRNAs are bound by Puf5 [43,48]. However, none of them were reported to bind to *YGP1* promoter or regulate *YGP1* expression. Therefore, we speculate that another factor than ones listed in Table 2 or 3 possibly mediates the regulation. Mutant screening that identifies genes upregulating *YGP1* expression in the *puf5*Δ background would be an effective approach to specify the downstream factor of Puf5.

### *YGP1* expression is regulated by dual mechanisms depending on glucose availability

**Fig 11:**
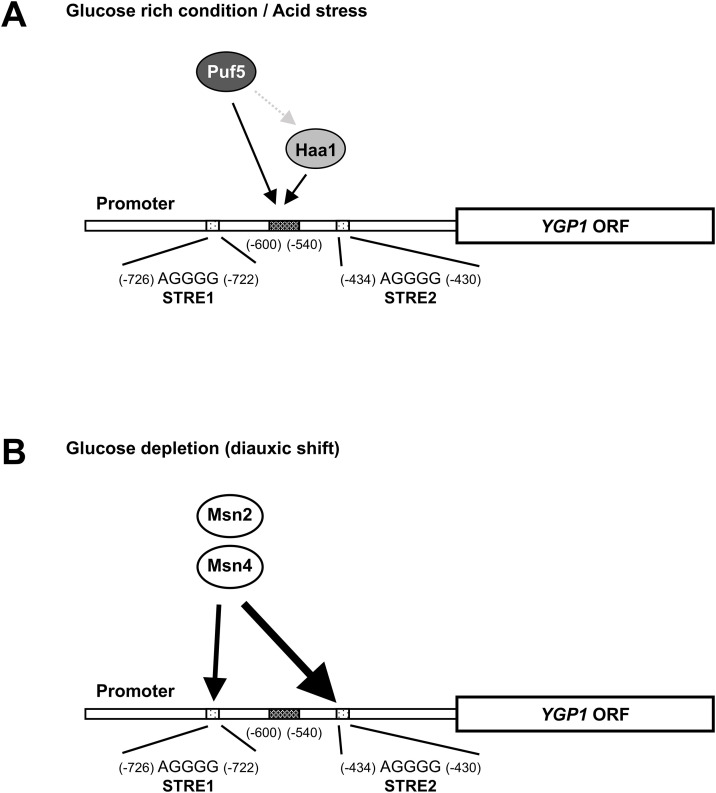
Working Model: *YGP1* is regulated by two mechanisms. (A) Puf5-dependent mechanism during glucose-rich condition, which is partly mediated by Haa1. This system regulates the *YGP1* expression during the logarithmic growth phase, thereby inducting M-phase specific expression of *YGP1*. Moreover, this regulation contributes to the acid stress responses and ensures long-term cell survival in the acid environment. (B) Msn2/Msn4-dependent mechanism at the diauxic shift. This pathway induces *YGP1* expression at the diauxic shift (glucose deprivation) by STRE2 within *YGP1* promoter. The Puf5-mediated regulation possibly contributes to this glucose-deprivation-responsive induction of *YGP1*, especially during the recovery from prior acid stress response.

We clarified that *YGP1* expression was highly induced at the diauxic shift by Msn2 and Msn4. Contrary to the logarithmic growth phase, Puf5 was not involved in this burst of the *YGP1* expression (Fig 1A). Therefore, the expression of the nutrition deprivation-responsive gene *YGP1* is regulated by two regulatory mechanisms: a Puf5-dependent mechanism and a Msn2/Msn4-dependent mechanism. The former maintains *YGP1* expression during logarithmic growth phase and also induces M-phase specific induction of *YGP1* expression during cell cycle (Fig 11A), which is partly mediated by a transcriptional activator Haa1. The latter ensures the glucose-deprivation responsive induction of *YGP1* expression via STRE2 within the *YGP1* promoter (Fig 11B). We propose that, since glucose is still present in the medium at low levels up to 9 h (Fig 1C), the regulation mediated by Puf5 predominantly governs *YGP1* expression during this glucose-available log phase condition. Upon entry into the diauxic shift at 12 h, Msn2 and Msn4 undergo rapid nuclear translocation, leading to robust transcriptional activation of *YGP1*, which likely overcomes regulation by Puf5. Moreover, when cells are exposed to the environment where acid metabolites accumulate, the Puf5-dependent mechanism is proposed to function in response to acid stress and ensure long-term survival.

### *YGP1* expression is required for long-term cell survival in the *puf5*Δ background

The Puf5-dependent regulatory pathway appears not to be involved in the diauxic shift-responsive induction of endogenous *YGP1* expression. However, when using the strain harboring a *YGP1*–3HA plasmid, the expression of *YGP1*–3HA mRNA and Ygp1–3HA protein were decreased in the *puf5*Δ mutant, accompanied by the decreased and delayed expression of stationary phase-responsive *HSP26* gene (Figs 8A–C). Similar expression patterns of *YGP1–3HA* and *HSP26* were also observed in the *haa1*Δ mutant (S4B Fig–S4D Fig). When culturing the strain harboring a YCplac33 plasmid, we pre-cultured the cells in SC-Ura medium and then shifted them to main culture in YPD medium. Previously, cells were reported to undergo the diauxic shift at 12 hours both in SC and YPD medium [44]. However, it has been revealed that the duration stationary phase yeast cells can survive (chronological life span) is much shorter in SC medium than in YPD medium [49]. This shortened lifespan is attributed to the rapid pH decrease caused by the accumulation of the metabolic byproduct acetic acid in SC medium [44]. Indeed, both the *puf5*Δ and *haa1*Δ single mutants were sensitive to acetic acid (Figs 10G and H). Moreover, the *puf5*Δ mutant exhibited low viability during long-term culture in SC medium, and the *puf5*Δ *ygp1*Δ double mutant showed an even more pronounced decrease (Fig 10F). Therefore, Puf5 and Haa1 are suggested to be involved in the acid stress responses and maintained *YGP1* expression contributes to maintaining long-term cell survival in the short-lived *puf5*Δ mutant background (Fig 11A). However, the *ygp1*Δ single mutant was not sensitive to acetic acid (Fig 10G). Previously, Haa1-mediated regulation of *YGP1* expression was reported to contribute to various acid stress responses [21]. In that study, the *ygp1*Δ mutant showed slightly slower growth in 60 mM acetic acid, but the effect of *ygp1*Δ mutation was more significant in the response to benzoic acid and octanoic acid. Notably, *YGP1* expression has been reported to be induced by propionic acid, lactic acid, acetic acid, and hydrochloric acid [21,23]. Therefore, we propose that Ygp1 acts downstream of the acetic acid stress response, although its physiological impact becomes evident only under relatively strong acid stress compared to acetic acid.

While the Puf5 and Haa1 are involved in the response to acetic acid, the sensitivity of the *puf5*Δ *haa1*Δ double mutant to acetic acid was stronger than the *puf5*Δ or *haa1*Δ single mutant (Fig 10H). Considering Puf5 functions both dependently and independently of Haa1, there appears to be the Haa1-independent regulatory pathway by Puf5 in acid stress responses. Previous genome wide screening of the mutation library for acid stress responses has revealed that various biological processes are involved in this process [23]. Interestingly, among the genes whose deletion enhances resistance or sensitivity to acid stress [23], approximately 50% genes showed the evidence of Puf5 binding to their transcribed mRNA [43,48]. In particular, *LRG1,* a previously identified target of Puf5 [30,37–39], was found to be a gene whose deletion increases resistance to acid stress [23]. *LRG1* encodes a GTPase-activating protein for Rho1 and regulates the MAPK pathway controlling cell wall integrity (CWI) [34–36]. Indeed, cell wall-related genes were reported to be important for organic acid responses [23]. Therefore, these findings suggest that Puf5 contributes to the acid stress responses by regulating multiple targets, with Haa1 being one important downstream factor of Puf5 in this process.

In addition, the *ygp1*Δ mutation accelerated the mortality of the *puf5*Δ mutant (Fig 10F). However, the survival rate of the *ygp1*Δ single mutant was comparable to that of the wild-type (Fig 10F). In contrary, the *ygp1*Δ mutant has previously been reported as a short-lifespan mutant [50]. In that study, cells were grown to complete entry into quiescence and then transferred to water to measure chronological lifespan. This indicates that Ygp1 is critical for survival in the quiescent state. Therefore, it is possible that extending the measurement period would reveal a decrease in long-term viability in the *ygp1*Δ mutant.

### The perspective of understanding Puf5-mediated regulatory network

The analysis of the *YGP1* regulatory machinery revealed a novel function of Puf5 in the acid stress responses. This finding indicates the importance of Puf5-mediated balancing of mRNA expression during stress adaptation. Although *YGP1* alone has minimal physiological significance, detailed analysis of such genes may lead to the discovery of new perspectives. Moreover, this study uncovered crosstalk between the acid stress response pathway mediated by an RNA-binding protein Puf5 and the general stress responsive transcriptional factors Msn2 and Msn4. Signals from accumulation of acidic metabolites or glucose deprivation appear to balance these two regulatory networks. Given that Ygp1 is a secretory glycoprotein, we propose that these pathways fine-tune *YGP1* induction to coordinate stress responses and cell survival, thereby contributing to environmental sensing. Because Puf5 is an RNA-binding protein targeting more than 1,000 mRNAs [43,48], it is possible that Puf5 acts as an upstream regulator of this tuning system. Recently, Pum1 and Pum2, functional orthologs of Puf5 in humans, were reported to target metabolism-related mRNAs and possibly contribute to the progression of cardiovascular diseases and cancers [51]. The tumor microenvironment is known to be acidic, which favors cancer cell survival [52]. In addition, glucose availability is closely linked to both pathologies. We believe that understanding the Puf5-mediated stress-responsive machinery will help uncover the broader Puf family regulatory network and its pathological significance.

## Supporting information

S1 Fig Biological replicates supporting Puf5-dependent rhythmic expression of *YGP1* in M-phase. The fold change of *RNR1* (A), *SIC1* (B), and *YGP1* (C) mRNA levels in the *bar1*Δ and *bar1*Δ *puf5*Δ mutants relative to the *bar1*Δ 0-min value at the indicated time points after release from G1-phase arrest. Data shown are Rq values from a second biological replicate to support Figs 3.
(TIF)

S2 Fig Biological replicates supporting the Ixr1-independent induction of *YGP1* in M-phase mediated by Puf5. The fold change of *YGP1* mRNA levels in the *bar1*Δ, *bar1*Δ *puf5*Δ, and *bar1*Δ *puf5*Δ *ixr1*Δ mutants relative to the *bar1*Δ 0-min value at the indicated time points after release from G1-phase arrest. Data shown are Rq values from a second biological replicate to support Fig 4B.
(TIF)

S3 Fig Biological replicates supporting the involvement of Haa1 in the rhythmic expression of *YGP1* in M-phase. The fold change of *RNR1* (A), *SIC1* (B), and *YGP1* (C) mRNA levels in the *bar1*Δ and *bar1*Δ *haa1*Δ mutants relative to the *bar1*Δ 0-min value at the indicated time points after release from G1-phase arrest. Data shown are Rq values from a second biological replicate to support Figs 7.
(TIF)

S4 Fig Haa1 is not involved in the induction of endogenous *YGP1* at the diauxic shift but contributes to the exogenous *YGP1* expression. (A) The fold change of *YGP1* mRNA levels in the wild-type, *puf5*Δ, *haa1*Δ, and *puf5*Δ *haa1*Δ mutants along a time course relative to the wild-type 4-hours value. The expression pattern was confirmed by two biological replicates, each having three technical replicates. The data shows the average Rq values ± SEM (n = 3 technical replicates from one representative biological replicate). (B) The fold change of *YGP1*–3HA mRNA levels expressed from a YCplac33 plasmid in the wild-type, *puf5*Δ, *haa1*Δ, and *puf5*Δ *haa1*Δ mutants along a time course relative to the wild-type 4-hours value. The expression pattern was confirmed by two biological replicates, each having three technical replicates. The data shows the the average Rq values ± SEM (n = 3 technical replicates from one representative biological replicate)). (C) The Ygp1–3HA protein levels expressed from a YCplac33 plasmid in the wild-type, *puf5*Δ, *haa1*Δ, and *puf5*Δ *haa1*Δ mutants. The image shows Ygp1–3HA and Pgk1 (loading control). The expression pattern was confirmed by two biological replicates, each having three technical replicates. The graph shows the mean ± SEM of the fold changes of Ygp1–3HA protein levels relative to that in wild-type 4 hours (n = 3 technical replicates from one representative biological replicate). (D) The fold change of *HSP26* mRNA levels in the wild-type, *puf5*Δ, *haa1*Δ, and *puf5*Δ *haa1*Δ mutants harboring a YCplac33-*YGP1*–3HA plasmid along a time course relative to the wild-type 4-hours value. The expression pattern was confirmed by two biological replicates, each having three technical replicates. The data shows the average Rq values ± SEM (n = 3 technical replicates from one representative biological replicate)).
(TIF)

S5 Fig The internal 240nt region in *YGP1* promoter is responsible for the induction of *YGP1* expression at the diauxic shift. (A) The left scheme represents a series of *YGP1* promoter deletions. Every 60-nucleotide region was deleted from a YCplac33-*YGP1* plasmid except for the region containing transcriptional start sites (TSSs). After introducing the deletion plasmids along with a full-length YCplac33-*YGP1* plasmid in the *ygp1*Δ strain, strains were pre-cultured in SC-Ura medium followed by the 2-hour exposure to small amount of YPD and thereafter main culture in YPD medium until mid-logarithmic phase (4 hours) or diauxic shift (9 hours). The *YGP1* expression was quantified by RT-qPCR. The right table shows the fold change of the average Rq values of *YGP1* mRNA from three technical replicates at the diauxic shift compared to that in the mid-logarithmic phase, along with the corresponding *p*-values. (♰) *p* < 0.05 and (♰♰) *p* < 0.01 are indicative of statistical significance. ns, no statistical significance (*p* < 0.05). (B) The fold changes of *YGP1* in the *ygp1*Δ mutant harboring a full-length YCplac33-*YGP1* plasmid or one of the deletion plasmids during the logarithmic phase (log) or diauxic shift. The data show the average Rq values ± SEM (n = 3 technical replicates) of fold change relative to that in the *ygp1*Δ [YCplac33-*YGP1*] logarithmic phase sample.
(TIF)

S1 Table Yeast strains used in this study. (DOCX)

S2 Table The primers used for gene deletions. (DOCX)

S3 Table The plasmids used in this study. (DOCX)

S4 Table The primers used for plasmid construction. (DOCX)

S5 Table The primers used for RT-qPCR. (DOCX)

S1 Raw Images The raw images of western-blotting gels. (PDF)

S1 Data set The numerical data set of shown data. (XLSX)
