# Supplementary material for: Dual regulatory mechanisms of YGP1 expression in response to glucose availability in Saccharomyces cerevisiae
Source: PLoS One. 2026 Aug 7;21(8):e0355681. doi: 10.1371/journal.pone.0355681 (PMC13450799; doi:10.1371/journal.pone.0355681)

Figure 6C_Ygp1-3HA

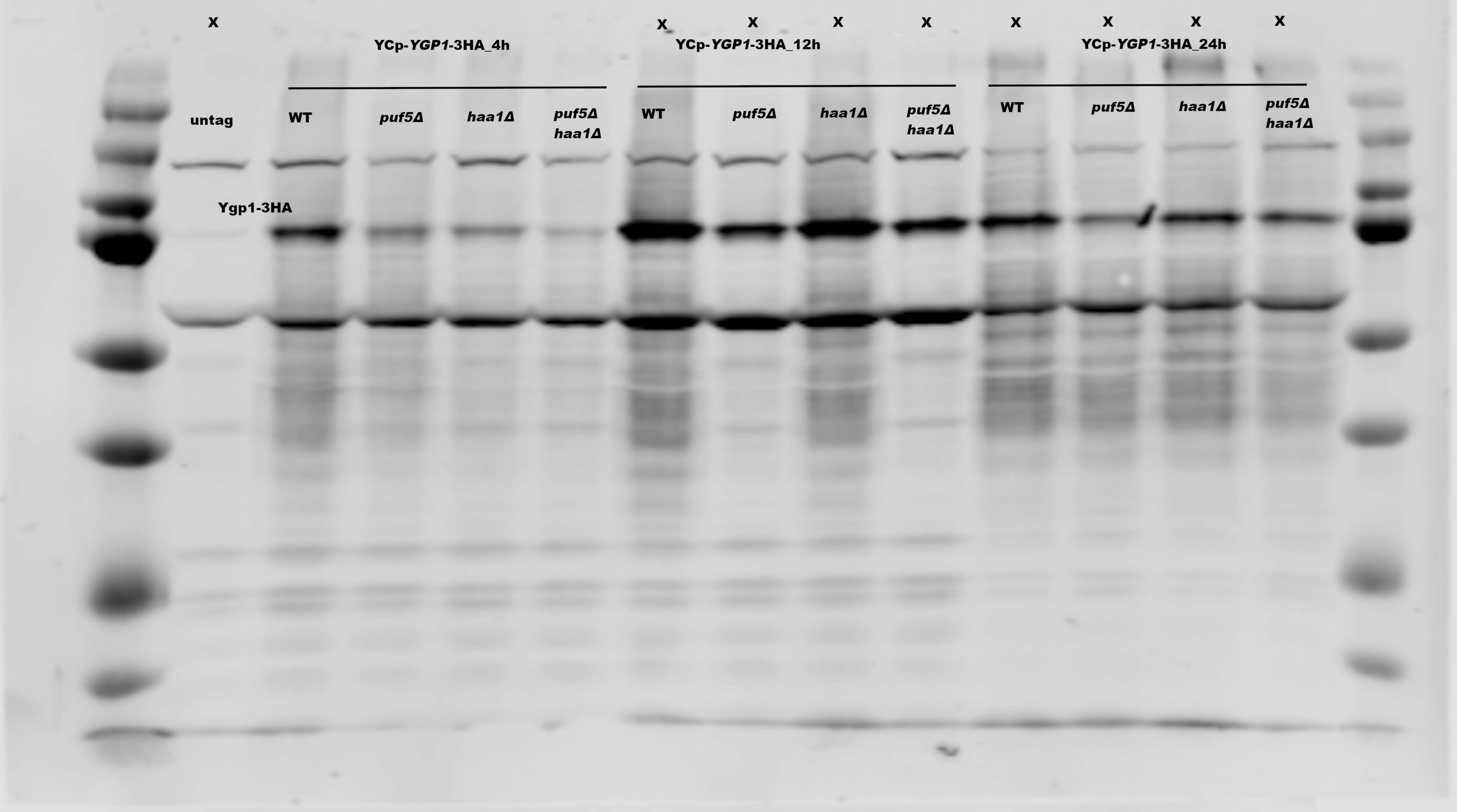

Figure 6C_Pgk1

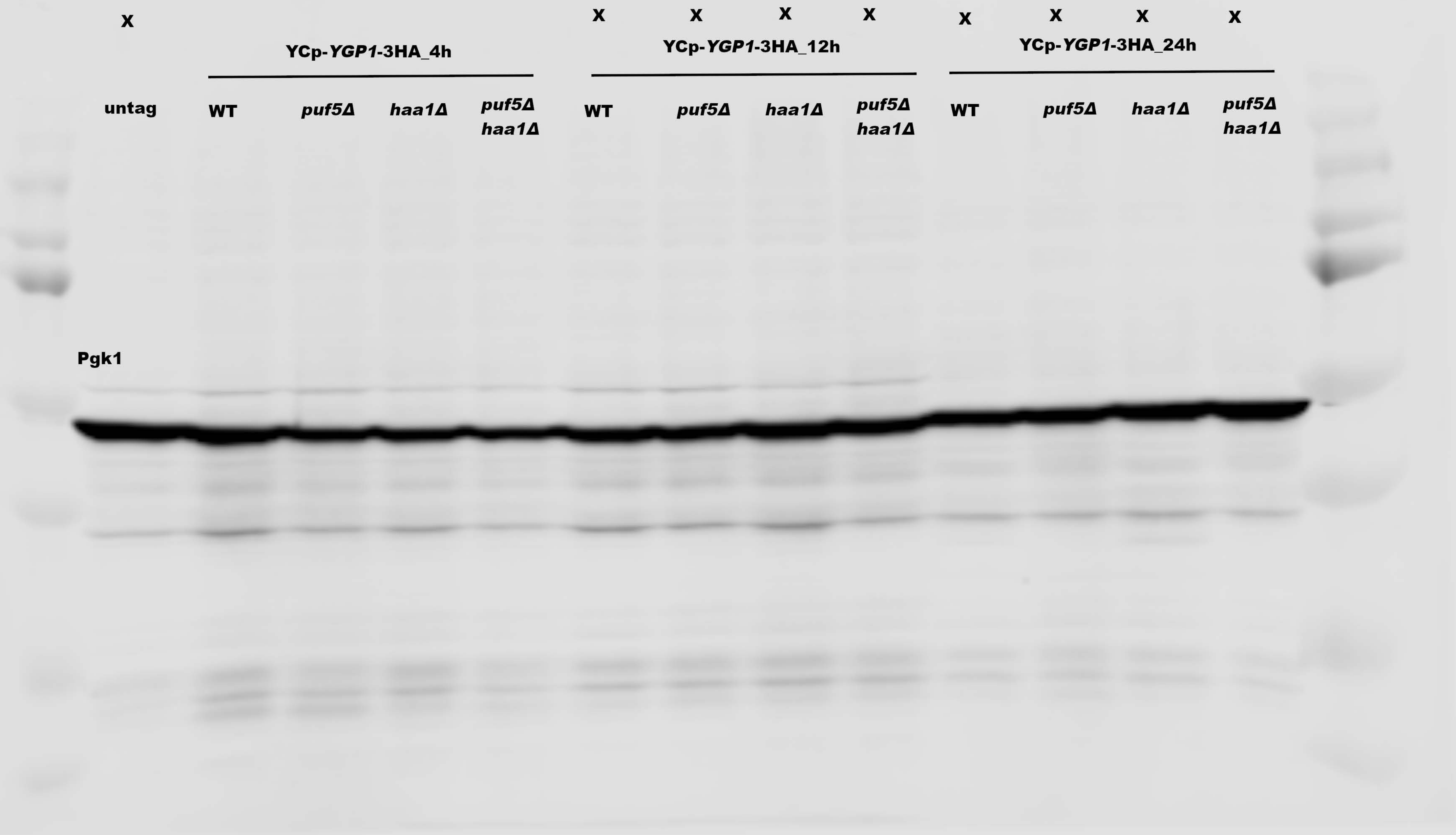

Figure 8B_Ygp1-3HA

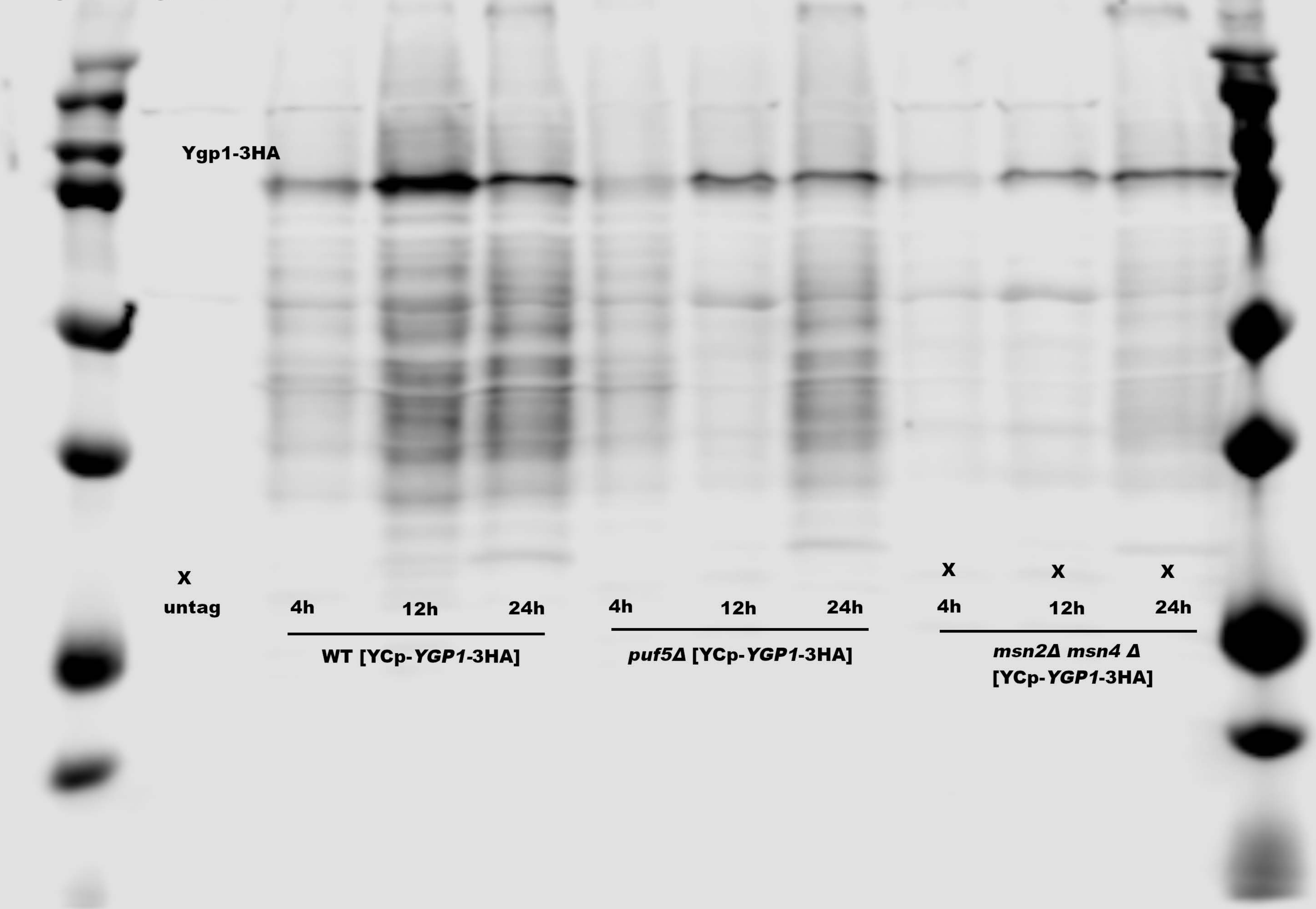

Figure 8B Pgk1

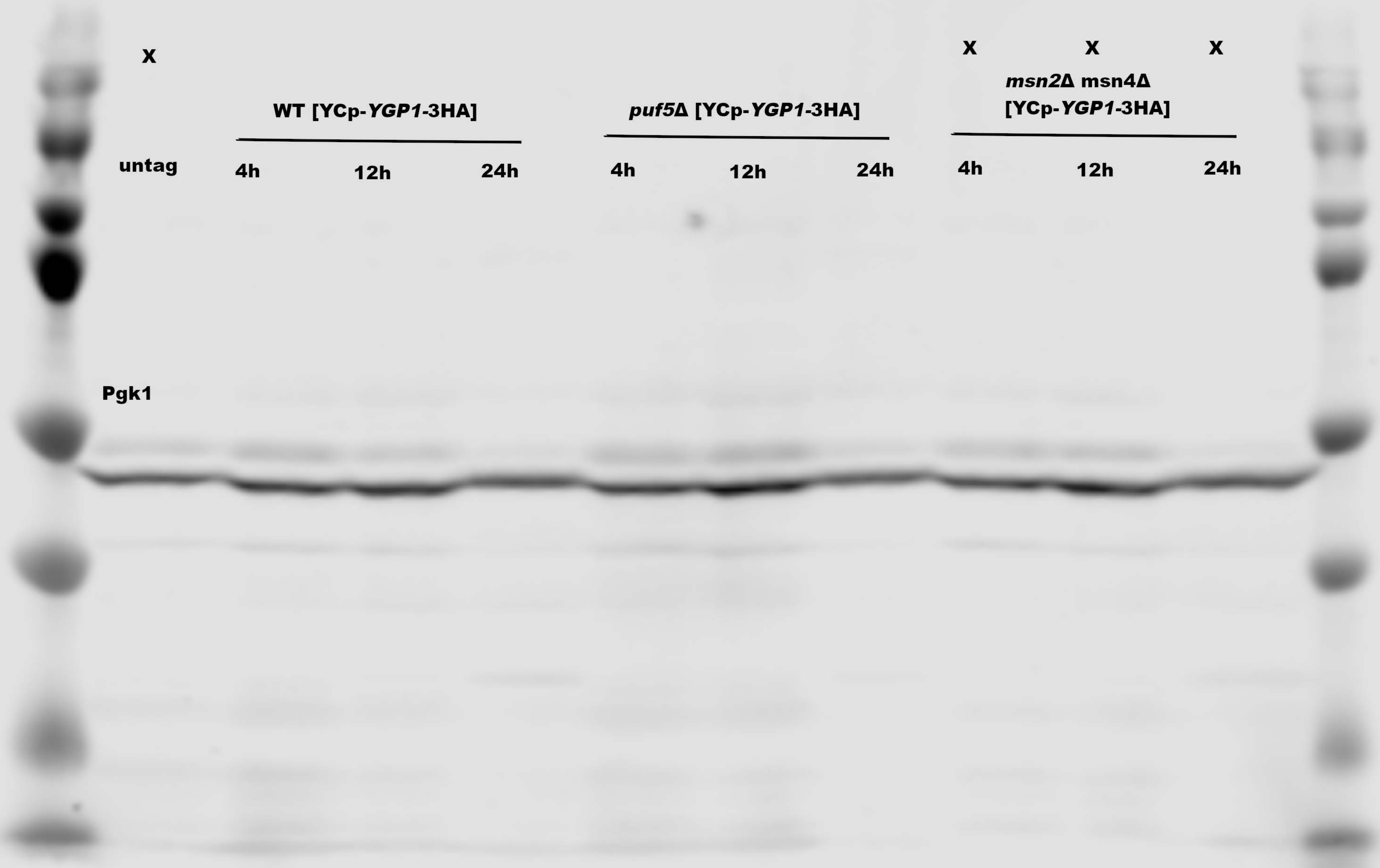

Figure 9D Ygp1-HA

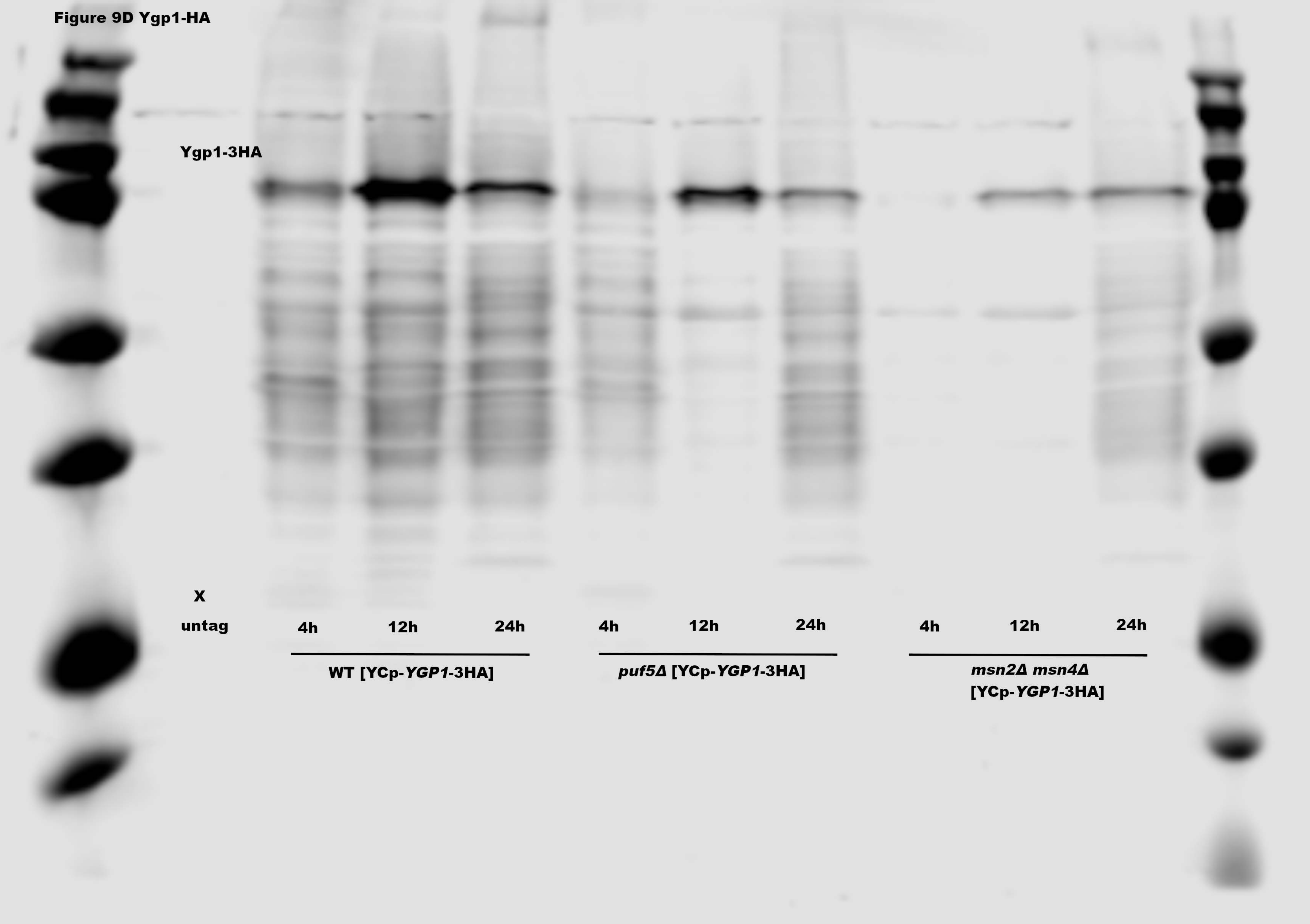

Figure 9D Pgk1

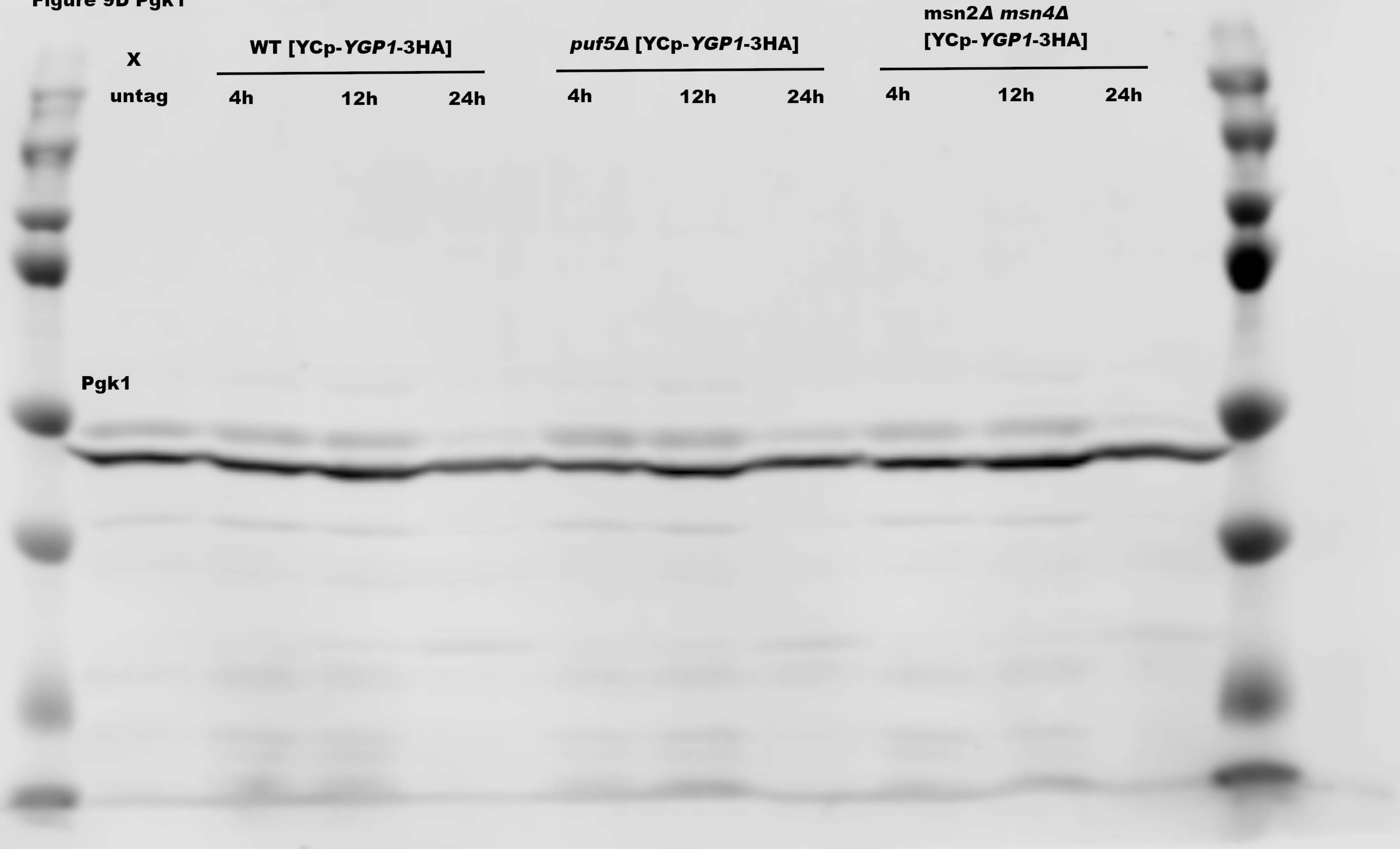

Figure S4C Ygp1-3HA

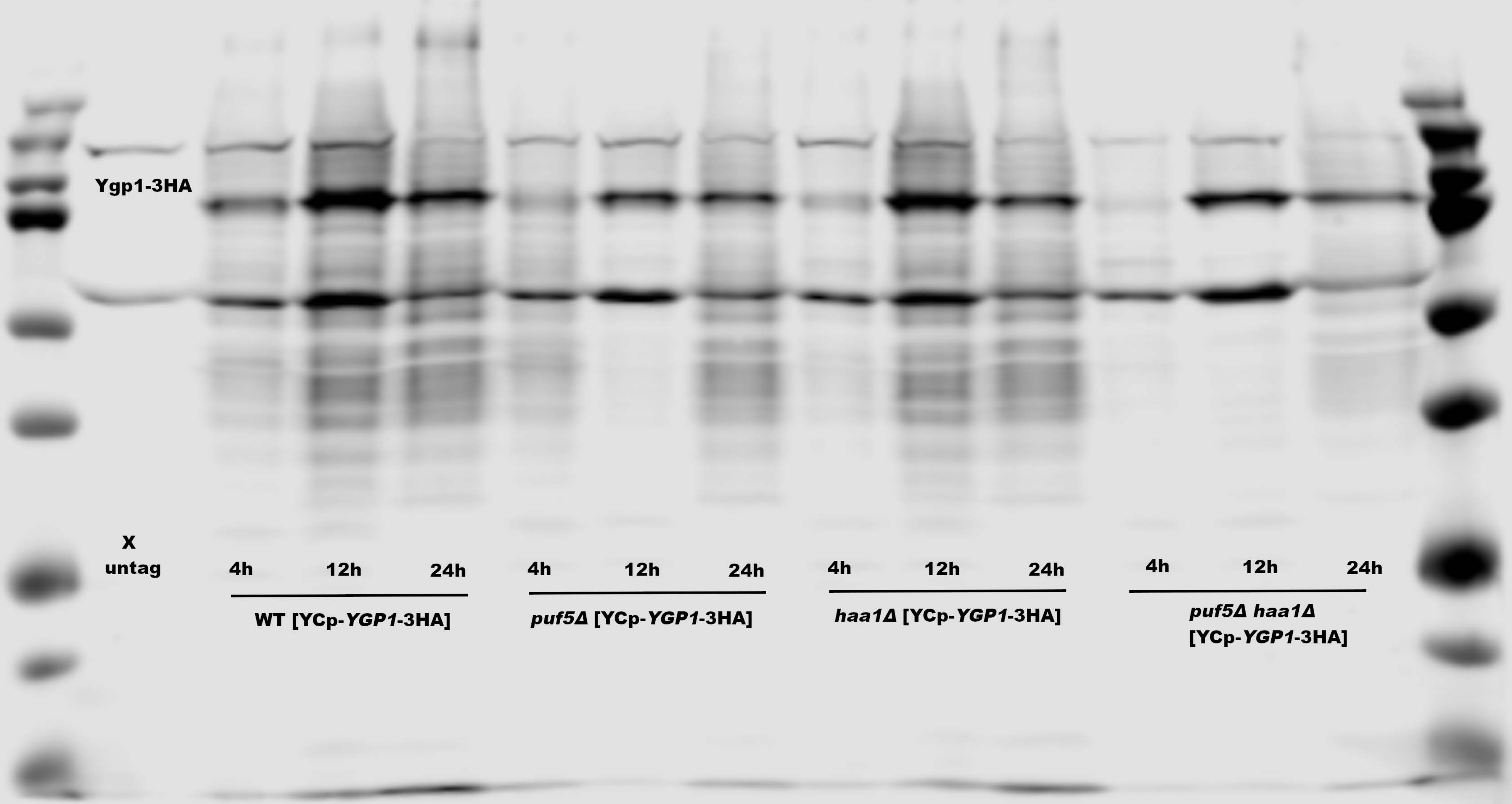

Figure S4C Pgk1

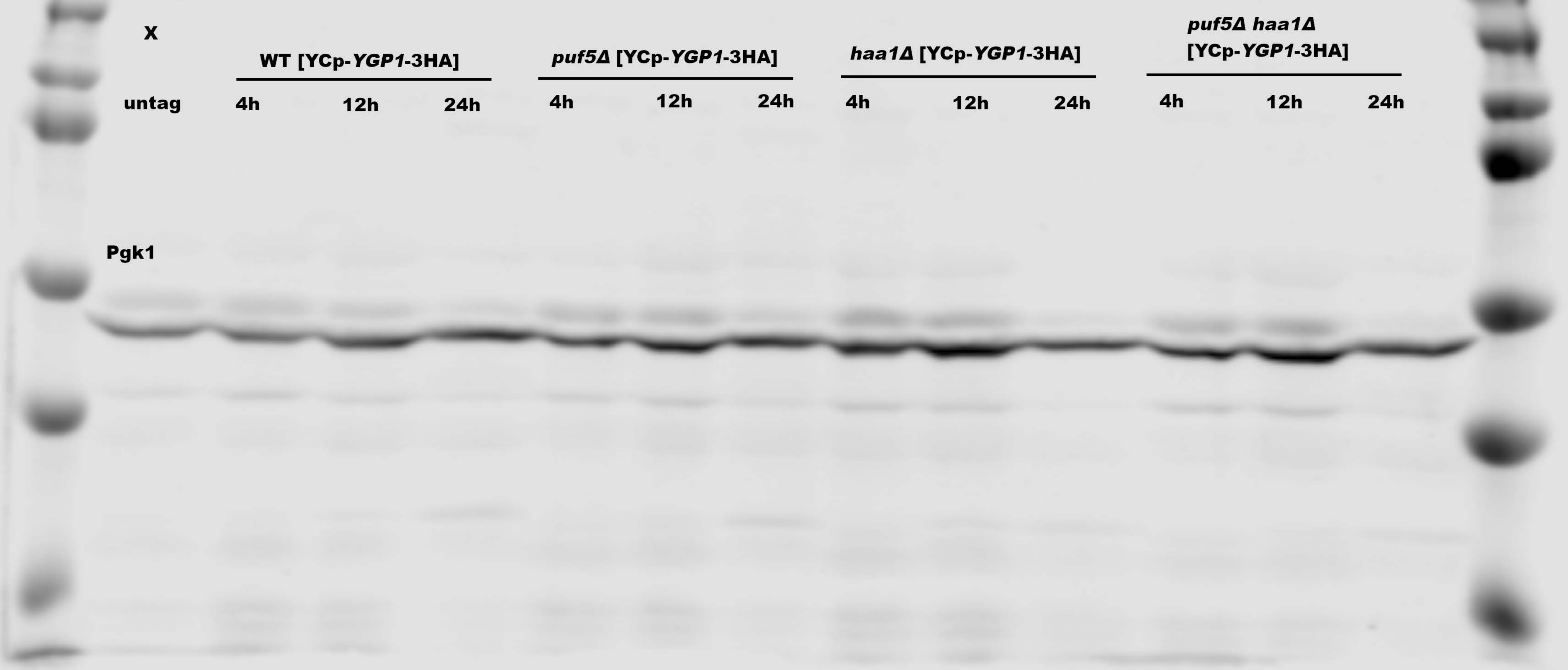

Supplement: Supplementary file 1 [file pone.0355681.s011.pdf]
