# Supplementary material for: Dual regulatory mechanisms of YGP1 expression in response to glucose availability in Saccharomyces cerevisiae
Source: PLoS One. 2026 Aug 7;21(8):e0355681. doi: 10.1371/journal.pone.0355681 (PMC13450799; doi:10.1371/journal.pone.0355681)
Supplement: Supplementary file 2 [file pone.0355681.s006.docx]

**S1 Table. Yeast strains used in this study.**

| Strain | Genotype | Source |
| --- | --- | --- |
| 10BD | *MATa/MATα ade2/ade2 trp1/trp1 can1/can1 leu2/leu2 his3/his3 ura3/ura3* | [1] |
| 1BD-*p5y* | *MATa/MATα ade2/ade2 trp1/trp1 can1/can1 leu2/leu2 his3/his3 ura3/ura3 PUF5/puf5Δ::CgTRP1 YGP1/ ygp1Δ::CgHIS3* | This study |
| 10BD-*p5h* | *MATa/MATα ade2/ade2 trp1/trp1 can1/can1 leu2/leu2 his3/his3 ura3/ura3 PUF5/puf5Δ::CgTRP1 HAA1/ haa1Δ::CgLEU2* | This study |
| 10BD-*bh* | *MATa/MATα ade2/ade2 trp1/trp1 can1/can1 leu2/leu2 his3/his3 ura3/ura3 BAR1/ bar1Δ::CgHIS3 HAA1/ haa1Δ::CgLEU2* | This study |
| WT | *MATa ade2 trp1 can1 leu2 his3 ura3* | This study |
| WT | *MATα ade2 trp1 can1 leu2 his3 ura3* | [2] |
| *puf5∆* | *MATa ade2 trp1 can1 leu2 his3 ura3 puf5Δ::CgTRP1* | This study |
| *puf5∆* | *MATa ade2 trp1 can1 leu2 his3 ura3 puf5Δ::CgHIS3* | [2] |
| *puf5∆* | *MATα ade2 trp1 can1 leu2 his3 ura3 puf5Δ::CgTRP1* | This study |
| *ixr1∆* | *MATa ade2 trp1 can1 leu2 his3 ura3 ixr1Δ::CgLEU2* | [2] |
| *puf5∆ ixr1∆* | *MATa ade2 trp1 can1 leu2 his3 ura3 puf5Δ::CgTRP1 ixr1Δ::CgLEU2* | [2] |
| *ygp1∆* | *MATa ade2 trp1 can1 leu2 his3 ura3 ygp1Δ::CgHIS3* | This study |
| *haa1∆* | *MATα ade2 trp1 can1 leu2 his3 ura3 haa1Δ::CgLEU2* | This study |
| *bar1∆* | *MATa ade2 trp1 can1 leu2 his3 ura3 bar1Δ::CgHIS3* | [2] |
| *msn2∆* | *MATa ade2 trp1 can1 leu2 his3 ura3 msn2Δ::kan* | [3] |
| *msn4∆* | *MATa ade2 trp1 can1 leu2 his3 ura3 msn4Δ::CgTRP1* | [3] |
| *puf5∆ ygp1∆* | *MATa ade2 trp1 can1 leu2 his3 ura3 puf5Δ::CgTRP1 ygp1Δ::CgHIS3* | This study |
| *puf5∆ haa1∆* | *MATα ade2 trp1 can1 leu2 his3 ura3 puf5Δ::CgTRP1 haa1Δ::CgLEU2* | This study |
| *bar1∆ puf5∆* | *MATa ade2 trp1 can1 leu2 his3 ura3 bar1Δ::CgHIS3 puf5Δ::CgTRP1* | [2] |
| *bar1∆ haa1∆* | *MATa ade2 trp1 can1 leu2 his3 ura3 bar1Δ::CgHIS3 haa1Δ::CgLEU2* | This study |
| *msn2∆ msn4∆* | *MATα ade2 trp1 can1 leu2 his3 ura3 msn2Δ::kan msn4Δ::CgTRP1* | [3] |

**References**

[1] Tadauchi T, Matsumoto K, Herskowitz I, Irie K. Post-transcriptional regulation through the HO 3'-UTR by Mpt5, a yeast homolog of Pumilio and FBF. *EMBO J*. 2001;20(3):552-561. doi:10.1093/emboj/20.3.552

[2] Sato M, Irie K, Suda Y, Mizuno T, Irie K. The RNA-binding protein Puf5 and the HMGB protein Ixr1 contribute to cell cycle progression through the regulation of cell cycle-specific expression of CLB1 in Saccharomyces cerevisiae. *PLoS Genet*. 2022;18(7):e1010340. Published 2022 Jul 29. doi:10.1371/journal.pgen.1010340

[3] Higuchi Y, Fujii S, Valderrama AL, et al. The eIF4E-binding protein Eap1 has similar but independent roles in cell growth and gene expression with the cytoplasmic deadenylase Ccr4. *Biosci Biotechnol Biochem*. 2021;85(6):1452-1459. doi:10.1093/bbb/zbab056
