# Supplementary material for: Dual regulatory mechanisms of YGP1 expression in response to glucose availability in Saccharomyces cerevisiae
Source: PLoS One. 2026 Aug 7;21(8):e0355681. doi: 10.1371/journal.pone.0355681 (PMC13450799; doi:10.1371/journal.pone.0355681)
Supplement: Supplementary file 3 [file pone.0355681.s007.docx]

**S2 Table. The primers used for gene deletions.**

| Gene | Forward | Reverse |
| --- | --- | --- |
| *YGP1* | AACATCTACAGGATTAATCGTCAGTTAAGTAATACAGTAATAGAAACACAGGAAACAGCTATGACC | GTAAAAAAAGAATCTCTATGCTTCGCTAGATTTAATATCTATCAGTGTTGTAAAACGACGGCCAGT |
| *HAA1* | CAAGAAAAAGGAAACAAAAGTATAGAAAAAAAAAACCTAAAAAATACACAGGAAACAGCTATGACC | AGGAAAACTACAGTTACAGAGAAGCAAGAGACGAAAAGCAAATTTAGTTGTAAAACGACGGCCAGT |
