# Supplementary material for: Dual regulatory mechanisms of YGP1 expression in response to glucose availability in Saccharomyces cerevisiae
Source: PLoS One. 2026 Aug 7;21(8):e0355681. doi: 10.1371/journal.pone.0355681 (PMC13450799; doi:10.1371/journal.pone.0355681)
Supplement: Supplementary file 4 [file pone.0355681.s008.docx]

**S3 Table. The plasmids used in this study.**

| Plasmid | Relevant markers | Source |
| --- | --- | --- |
| YEplac195 | *URA3* | [1] |
| YEplac195-*PUF5* | *URA3, PUF5* | [2] |
| YEp13 | *LEU2* | [2] |
| YEp13-*PUF5* | *LEU2, PUF5* | [2] |
| YCplac33-*YGP1*promoter-*GFP*-*ADH1* 3’UTR | *URA3, CEN-ARS, GFP* | This study |
| YCplac33-*MCM2*promoter-*GFP*-*ADH1* 3’UTR | *URA3, CEN-ARS, GFP* | This study |
| YCplac33-*MCM2*promoter-*GFP*-*YGP1* 3’UTR | *URA3, CEN-ARS, GFP* | This study |
| YCplac33*-YGP1* | *URA3, CEN-ARS, YGP1* | This study |
| YCplac33-*YGP1*-3HA | *URA3, CEN-ARS, YGP1-*3HA | This study |
| YCplac33*-YGP1*-del1 | *URA3, CEN-ARS, YGP1* | This study |
| YCplac33*-YGP1*-del2 | *URA3, CEN-ARS, YGP1* | This study |
| YCplac33*-YGP1*-del3 | *URA3, CEN-ARS, YGP1* | This study |
| YCplac33*-YGP1*-del4 | *URA3, CEN-ARS, YGP1* | This study |
| YCplac33*-YGP1*-del5 | *URA3, CEN-ARS, YGP1* | This study |
| YCplac33*-YGP1*-del6 | *URA3, CEN-ARS, YGP1* | This study |
| YCplac33*-YGP1*-del7 | *URA3, CEN-ARS, YGP1* | This study |
| YCplac33*-YGP1*-del8 | *URA3, CEN-ARS, YGP1* | This study |
| YCplac33*-YGP1*-del9 | *URA3, CEN-ARS, YGP1* | This study |
| YCplac33*-YGP1*-del10 | *URA3, CEN-ARS, YGP1* | This study |
| YCplac33*-YGP1*-del11 | *URA3, CEN-ARS, YGP1* | This study |
| YCplac33*-YGP1*-del12 | *URA3, CEN-ARS, YGP1* | This study |
| YCplac33*-YGP1*-del13 | *URA3, CEN-ARS, YGP1* | This study |
| YCplac33*-YGP1*-del14 | *URA3, CEN-ARS, YGP1* | This study |
| YCplac33*-YGP1*-STRE1del | *URA3, CEN-ARS, YGP1* | This study |
| YCplac33*-YGP1*-STRE2del | *URA3, CEN-ARS, YGP1* | This study |
| pCgLEU2 | *C. glabrata LEU2* in pUC19 | [3] |
| pCgHIS3 | *C. glabrata HIS3* in pUC19 | [3] |
| pFA6a-3HA-kanMX6 | *3HA-ADH1* terminator, kanamycin resistance cassette | [4] |

**References**

[1] Gietz RD, Sugino A. New yeast-Escherichia coli shuttle vectors constructed with in vitro mutagenized yeast genes lacking six-base pair restriction sites. *Gene*. 1988;74(2):527-534. doi:10.1016/0378-1119(88)90185-0

[2] Sato M, Irie K, Suda Y, Mizuno T, Irie K. The RNA-binding protein Puf5 and the HMGB protein Ixr1 contribute to cell cycle progression through the regulation of cell cycle-specific expression of CLB1 in Saccharomyces cerevisiae. *PLoS Genet*. 2022;18(7):e1010340. Published 2022 Jul 29. doi:10.1371/journal.pgen.1010340

[3] Sakumoto N, Mukai Y, Uchida K, et al. A series of protein phosphatase gene disruptants in Saccharomyces cerevisiae. *Yeast*. 1999;15(15):1669-1679. doi:10.1002/(SICI)1097-0061(199911)15:15<1669::AID-YEA480>3.0.CO;2-6

[4] Longtine MS, McKenzie A 3rd, Demarini DJ, et al. Additional modules for versatile and economical PCR-based gene deletion and modification in Saccharomyces cerevisiae. *Yeast*. 1998;14(10):953-961. doi:10.1002/(SICI)1097-0061(199807)14:10<953::AID-YEA293>3.0.CO;2-U
