# Supplementary material for: Dual regulatory mechanisms of YGP1 expression in response to glucose availability in Saccharomyces cerevisiae
Source: PLoS One. 2026 Aug 7;21(8):e0355681. doi: 10.1371/journal.pone.0355681 (PMC13450799; doi:10.1371/journal.pone.0355681)
Supplement: Supplementary file 5 [file pone.0355681.s009.docx]

**S4 Table. The primers used for plasmid construction.**

| Fragments | Forward primer | Reverse primer |
| --- | --- | --- |
| *MCM2*promoter-GFP | caagcttgcatgcctgcaggtcgacAAAGGCGTCTATCCACTTGC | GCTAGATTTAATATCTATCAGTGGCGCGCCCTATTTGTATAGTTC |
| *YGP1* 3’UTR for reporter construction | GAACTATACAAATAGGGCGCGCCACTGATAGATATTAAATCTAGC | ttgtaaaacgacggccagtgaattcGGGTGAGATCCCTTATCTGTAG |
| *YGP1*promoter-ORF-3’UTR | caagcttgcatgcctgcaggtcgacGACGGAATCCCTAATCATCTTTGG | ttgtaaaacgacggccagtgaattcGGGTGAGATCCCTTATCTGTAG |
| YGP1promoter-ORF | caagcttgcatgcctgcaggtcgacGACGGAATCCCTAATCATCTTTGG | gttaattaacccggggatccgTGGGAAAATGCTTTCCAGAGCGGA |
| 3HA | TCCGCTCTGGAAAGCATTTTCCCAcggatccccgggttaattaac | GCTAGATTTAATATCTATCAGTTTATTTAGAAGTGGCGCGCCTCAGCA |
| *YGP1* 3’UTR for 3HA tagging | TGCTGAGGCGCGCCACTTCTAAATAAACTGATAGATATTAAATCTAGC | ttgtaaaacgacggccagtgaattcGGGTGAGATCCCTTATCTGTAG |
| *YGP1*del1 | caagcttgcatgcctgcaggtcgacTTGAAGGAAAGAGAAAAATGAGCA | ttgtaaaacgacggccagtgaattcGGGTGAGATCCCTTATCTGTAG |
| *YGP1*del2-up | caagcttgcatgcctgcaggtcgacGACGGAATCCCTAATCATCTTTGG | CTCGCCGCCACATTACCCAATAGGACCATCGTCGGTAAAAGAAGGGAG |
| *YGP1*del2-down | CTCCCTTCTTTTACCGACGATGGTCCTATTGGGTAATGTGGCGGCGAG | ttgtaaaacgacggccagtgaattcGGGTGAGATCCCTTATCTGTAG |
| *YGP1*del3-up | caagcttgcatgcctgcaggtcgacGACGGAATCCCTAATCATCTTTGG | CCCGATCCGAGAATTTGAACATTGGAATTGAAATGGAAATCGACTACT |
| *YGP1*del3-down | AGTAGTCGATTTCCATTTCAATTCCAATGTTCAAATTCTCGGATCGGG | ttgtaaaacgacggccagtgaattcGGGTGAGATCCCTTATCTGTAG |
| *YGP1*del4-up | caagcttgcatgcctgcaggtcgacGACGGAATCCCTAATCATCTTTGG | ATGTGTCTTCAATCCCACATCAAGTGAGAACATTGAATCAATGATACC |
| *YGP1*del4-down | GGTATCATTGATTCAATGTTCTCACTTGATGTGGGATTGAAGACACAT | ttgtaaaacgacggccagtgaattcGGGTGAGATCCCTTATCTGTAG |
| *YGP1*del5-up | caagcttgcatgcctgcaggtcgacGACGGAATCCCTAATCATCTTTGG | ACCAACGAATGGCCGATGTTTTACGCCCCTTCCTCTGGAAAGAGATCC |
| *YGP1*del5-down | GGATCTCTTTCCAGAGGAAGGGGCGTAAAACATCGGCCATTCGTTGGT | ttgtaaaacgacggccagtgaattcGGGTGAGATCCCTTATCTGTAG |
| *YGP1*del6-up | caagcttgcatgcctgcaggtcgacGACGGAATCCCTAATCATCTTTGG | CTTCCGCGGATGACGAGAAAACTTGGGTAAGCAAAAGTTTCGACGGTG |
| *YGP1*del6-down | CACCGTCGAAACTTTTGCTTACCCAAGTTTTCTCGTCATCCGCGGAAG | ttgtaaaacgacggccagtgaattcGGGTGAGATCCCTTATCTGTAG |
| *YGP1*del7-up | caagcttgcatgcctgcaggtcgacGACGGAATCCCTAATCATCTTTGG | ACATGTCCACATAACATGCCCCGACGCAAAAAAAAAAGTCACAAAAAA |
| *YGP1*del7-down | TTTTTTGTGACTTTTTTTTTTGCGTCGGGGCATGTTATGTGGACATGT | ttgtaaaacgacggccagtgaattcGGGTGAGATCCCTTATCTGTAG |
| *YGP1*del8-up | caagcttgcatgcctgcaggtcgacGACGGAATCCCTAATCATCTTTGG | AAAGGGTAAACTATTTAGACAGTGTTGGGTAAAAGTGAGAGACTTTTT |
| *YGP1*del8-down | AAAAAGTCTCTCACTTTTACCCAACACTGTCTAAATAGTTTACCCTTT | ttgtaaaacgacggccagtgaattcGGGTGAGATCCCTTATCTGTAG |
| *YGP1*del9-up | caagcttgcatgcctgcaggtcgacGACGGAATCCCTAATCATCTTTGG | TAAACTTTCGTCAACTTCTGCGCTTACCCTTTTTGGGCTCATGTGTCT |
| *YGP1*del9-down | AGACACATGAGCCCAAAAAGGGTAAGCGCAGAAGTTGACGAAAGTTTA | ttgtaaaacgacggccagtgaattcGGGTGAGATCCCTTATCTGTAG |
| *YGP1*del10-up | caagcttgcatgcctgcaggtcgacGACGGAATCCCTAATCATCTTTGG | ATGCAATAGAGTACGAATATATCCAATTTTCGGCCCCTTCTTCGAGAA |
| *YGP1*del10-down | TTCTCGAAGAAGGGGCCGAAAATTGGATATATTCGTACTCTATTGCAT | ttgtaaaacgacggccagtgaattcGGGTGAGATCCCTTATCTGTAG |
| *YGP1*del11-up | caagcttgcatgcctgcaggtcgacGACGGAATCCCTAATCATCTTTGG | TTGAACTGTAGCATCGAGAAGCCGCTTTTCAGCTCCTGAGCCTCTCCA |
| *YGP1*del11-down | TGGAGAGGCTCAGGAGCTGAAAAGCGGCTTCTCGATGCTACAGTTCAA | ttgtaaaacgacggccagtgaattcGGGTGAGATCCCTTATCTGTAG |
| *YGP1*del12-up | caagcttgcatgcctgcaggtcgacGACGGAATCCCTAATCATCTTTGG | ATTATTACTAATAGGGTAAAAGAGGCTTCACTCCCTACTGAGATTCTT |
| *YGP1*del12-down | AAGAATCTCAGTAGGGAGTGAAGCCTCTTTTACCCTATTAGTAATAAT | ttgtaaaacgacggccagtgaattcGGGTGAGATCCCTTATCTGTAG |
| *YGP1*del13-up | caagcttgcatgcctgcaggtcgacGACGGAATCCCTAATCATCTTTGG | TTTTTATATTTCAGAGATGATGGTATATAATAGGCGCTTTCTTTCTTC |
| *YGP1*del13-down | GAAGAAAGAAAGCGCCTATTATATACCATCATCTCTGAAATATAAAAA | ttgtaaaacgacggccagtgaattcGGGTGAGATCCCTTATCTGTAG |
| *YGP1*del14-up | caagcttgcatgcctgcaggtcgacGACGGAATCCCTAATCATCTTTGG | TGTAGATGTTTTTTGAGCTGATTTAGCAGAAGTTCTAGCAAATGTCAC |
| *YGP1*del14-down | GTGACATTTGCTAGAACTTCTGCTAAATCAGCTCAAAAAACATCTACA | ttgtaaaacgacggccagtgaattcGGGTGAGATCCCTTATCTGTAG |
| *YGP1*STRE1-del-up | caagcttgcatgcctgcaggtcgacGACGGAATCCCTAATCATCTTTGG | TGTGTCTTCAATCCCACATCAAGGTCCTCTGGAAAGAGATCCCAATTA |
| *YGP1*STRE1-del-down | TAATTGGGATCTCTTTCCAGAGGACCTTGATGTGGGATTGAAGACACA | ttgtaaaacgacggccagtgaattcGGGTGAGATCCCTTATCTGTAG |
| *YGP1*STRE2-del-up | caagcttgcatgcctgcaggtcgacGACGGAATCCCTAATCATCTTTGG | GTCAACTTCTGCGCTAATTTTCGGTCTTCGAGAAGTTTCGATTCGTAA |
| *YGP1*STRE2-del-down | TTACGAATCGAAACTTCTCGAAGACCGAAAATTAGCGCAGAAGTTGAC | ttgtaaaacgacggccagtgaattcGGGTGAGATCCCTTATCTGTAG |
