# Supplementary material for: Dual regulatory mechanisms of YGP1 expression in response to glucose availability in Saccharomyces cerevisiae
Source: PLoS One. 2026 Aug 7;21(8):e0355681. doi: 10.1371/journal.pone.0355681 (PMC13450799; doi:10.1371/journal.pone.0355681)
Supplement: Supplementary file 6 [file pone.0355681.s010.docx]

**S5 Table. The primers used for RT-qPCR.**

| Primer | Sequence |
| --- | --- |
| *SCR1*-forward | AACCGTCTTTCCTCCGTCGTAA |
| *SCR1*-reverse | CTACCTTGCCGCACCAGACA |
| *YGP1*-forward | ACGCTACCAAACGTGGTGTC |
| *YGP1*-reverse | CAGGCAAACCAGCACCGTAA |
| *GFP*-forward | CACTGGAGTTGTCCCAATTCTTG |
| *GFP*-reverse | TCCGTATGTTGCATCACCTTCA |
| *RNR1*-forward | CCATGGCACCAATGCCTACT |
| *RNR1-*reverse | CACCGGATAAGACACGACGG |
| *SIC1*-forward | GGCTTACGTCTCCTCAACGC |
| *SIC1*-reverse | CGACCCAATGGTTCCTGCTC |
| *HAA1*-forward | ACCGCTTATGATGATCAAACCCA |
| *HAA1*-reverse | CCACACGTGCAAACACCTTC |
| *HSP26*-forward | TGATGCCTTTAACAGATTGCTG |
| *HSP26*-reverse | CCTTTGCGGGTGTGTTTG |
| *YGP1*-3HA-forward | CTCCAAGTCCGCTCTGGAAAG |
| *YGP1*-3HA-reverse | CCCGCATAGTCAGGAACATCG |
