# Supplementary material for: Dual regulatory mechanisms of YGP1 expression in response to glucose availability in Saccharomyces cerevisiae
Source: PLoS One. 2026 Aug 7;21(8):e0355681. doi: 10.1371/journal.pone.0355681 (PMC13450799; doi:10.1371/journal.pone.0355681)
Supplement: Supplementary file 7 [file pone.0355681.s014.docx]

**Our responses to the reviewers’ comments and changes in the revised manuscript**

Title: Dual regulatory mechanisms of *YGP1* expression in response to glucose availability in *Saccharomyces cerevisiae*.

Authors: Megumi Sato, Kaoru Irie, Yasuyuki Suda, Tomoaki Mizuno, Kenji Irie

Regarding the editorial summary points about the Statistical Analysis:

We have clarified the nature of the data shown in each figure in the corresponding figure legends.

Regarding the use of fold change values throughout the manuscript, mRNA expression levels were quantified by RT-qPCR using the ΔΔCt method, and Rq values relative to a defined reference sample were therefore presented as fold changes. The underlying Ct values for all experiments are provided in a numerical data file (S1 Data set). For protein quantification by Western blotting, absolute signal intensities varied substantially between experiments due to unavoidable technical variability, such as repeated use of primary and secondary antibodies. Because these raw signal intensities are not directly comparable across experiments, protein levels were normalized to the endogenous control Pgk1, and fold changes relative to a defined reference sample were used for presentation.

Taken together, fold change representation was intentionally employed to enable meaningful comparison across experiments, rather than reflecting a lack of reproducibility. Where applicable, biological replicates are shown and described, and the nature of the analysis (quantitative vs. qualitative) is explicitly stated in the manuscript.

Further details are provided in the point-by-point responses below.

Reviewer #1

Major:
1) The relationship between glucose availability and PUF5, MSN2, and MSN4 needs to be clearly defined. The authors should summarize the known roles of these proteins in S. cerevisiae, particularly under glucose-rich versus glucose-depleted conditions. Without this context, the proposed regulatory model lacks mechanistic support, it will be better having those genes expression level along with the glucose availability in S. cerevisiae.

The authors should directly present the YGP1 expression data together with the glucose concentration over time, rather than relying on cited literature and the culture time.

(response)

Based on the reviewer’s comments, we added descriptions of previously reported functions of Puf5 to the Results section (page 10, lines 203–209) and incorporated additional information regarding Msn2/Msn4 into the relevant sections of the Introduction (page3, lines 47–56) and Results (pages 21–22, lines 503–508).

In addition, we measured glucose concentrations in the culture medium during time-course cultivation, and the data are presented in Figure 1C. The corresponding results are described in the Results section (page9, lines 193–195 and page10, lines 215–216).

2) The conclusion that PUF5 regulates YGP1 via its promoter is not fully supported. The current reporter construct (YGP1 promoter-GFP-ADH1 3’UTR) does not test 3’ UTR-dependent regulation. A construct containing the YGP1 3’UTR should be included. Direct evidence of PUR5 binding with YGP1 promoter (e.g. EMSA) is also needed.

(response)

In response to the reviewer’s comments, we investigated the contribution of the *YGP1* 3’UTR by constructing *MCM2*promoter–*GFP–ADH1* 3’UTR and *MCM2*promoter–*GFP–YGP1* 3’UTR reporter constructs. *GFP* reporter expression was analyzed in wild-type and *puf5*Δ mutant cells. We found that neither reporter exhibited a significant difference in expression in the *puf5*Δ mutant compared with wild-type cells. These results indicate that the regulatory target of Puf5 is likely the *YGP1* promoter, rather than its 3’UTR. These results are shown in Figure 2G and are described in the Results section (page 11, lines 245–251).

Furthermore, to examine whether Puf5 directly binds to the *YGP1* promoter, we performed a ChIP assay to assess Puf5 association with a 60-nucleotide region of the *YGP1* promoter (−600 to −540). However, we were unable to detect enrichment of this chromatin region in our ChIP analysis. These results suggest that regulation of *YGP1* by Puf5 is likely indirect, rather than mediated by direct binding of Puf5 to the *YGP1* promoter.

3) The functional relevance of YGP1 should be clarified first. Since the manuscript focus on YGP1 regulation, its physiological role under the tested conditions should be clearly demonstrated before dissecting its regulatory mechanisms.

(response)

In accordance with the reviewer’s suggestions, we revised the structure of the Results section. We now begin the Results by describing previously reported and proposed functions of Ygp1 (page9, lines 182–188), followed by presentation of our analysis of the regulatory mechanisms governing *YGP1* expression.

4) The transfer of plasmid-bearing strains from SC-ura to YPD may result in plasmid loss due to lack of selection (as this method is used to loop out the pCUT plasmid during yeast strains construction). This raises concerns about data reliability and must be clarified.

(response)

Following the reviewer’s suggestion, we assessed plasmid retention in plasmid-bearing strains. Plasmid carriage reates after 24 h of cultivation in YPD medium, following pre-culture in SC-Ura, are shown in Tables 4, 5, and 7. All strains exhibited plasmid retention rates above 95%, with no significant differences among strains.

5) Overall, the manuscript would benefit from a clearer structure: 1) demonstrate the YGP1 expression dynamics with glucose levels; 2) systematically analyze the role of PUF5, HAA1, MSN2, and MSN4 in glucose-dependent regulation; 3) Compare YGP1 expression in different genetic backgrounds, including deletion and overexpression of the regulatory factors; 4) identify and characterization the promoter elements of YGP1; 5) evaluate growth phenotypes of YGP1 mutant strains in different genetic background under different culture conditions. As currently presented, the manuscript appears more focused on PUF5 than on YGP1, which makes the central theme unclear

(response)

Based on the reviewer’s comments, we revised the overall structure of the manuscript. To more clearly distinguish the regulatory mechanisms operating during the logarithmic phase and at the diauxic shift, we first present the time-course expression profile of *YGP1* in the revised manuscript and figures. The subsequent structure largely follows that of the originally submitted version; however, we revised the text where appropriate to improve clarity and ensure a coherent logical flow.

Minors:
1) Figure 2 lacks error bars, although the authors state that n = 3 biological replicates were performed.

(response)

In the originally submitted version, Figure 2 (revised as Figure 3) showed representative values from three technical replicates. Reproducibility was confirmed using an additional independent biological replicate, and these data are now presented in Figure S1.

2) Lines 274–287, The results and discussion in this section may be affected by the problematic culture conditions described above.

(response)

As described above, plasmid retention rates did not differ significantly among strains under the culture conditions used. Therefore, we regard that the influence of culture conditions has been appropriately considered, and the interpretation presented in the manuscript remains valid.

3) Line 319 / Figure 8B, What explains the increase in YGP1 expression at 9 h and 24 h? This requires clarification.

As shown in Figure 1C, glucose concentrations in the culture medium during cultivation of wild-type and *puf5*Δ cells were already close to depletion at the 9h time point. We therefore consider that slight differences in the initial glucose concentrations during medium preparation may have resulted in minor variations in the timing of glucose depletion. This explanation has been added to the Results section (page22, lines 514–520).

4) Line 329, correct “ling-term” to “long-term”.

(response)

We carefully checked the manuscript and corrected this typographical error on page 25 line 572.

5) Figure 9F, did the authors test a PUF5 deletion strain combined with YGP1 overexpression?

(response)

In response to the reviewer’s comments, we examined the effect of a YEp‑*YGP1* on cell viability during long-term cultivation of the *puf5*Δ mutant. However, as shown in the graph below, we did not observe an obvious recovery of viability by the YEp‑*YGP1*. However, we could not confirm whether the plasmid overexpressed a fully functional form of *YGP1*. In addition, the reduced viability of the *puf5*Δ *ygp1*Δ double mutant was consistently reproducible (n=2). Therefore, we speculate that Ygp1 plays a supportive role in long-term survival under *puf5*Δ conditions.

Cell Viability (%)

Viability (%)

|  | log | 7d |
| --- | --- | --- |
| WT YEp195 | 92.2667 | 88.6333 |
| WT YEp195-YGP1 | 88.5667 | 83.6 |
| puf5Δ YEp195 | 82.8667 | 58.6333 |
| puf5Δ YEp195-YGP1 | 77.2333 | 56.9667 |

**# Reviewer 2**

Major comments:

1. Time course experiments Figure 2,3,6:

The experiments are described as representative data of three experiments, without showing data on replication. In reference to these figures, the authors discuss temporal change. While I understand that the effect of the mutations discussed on YPG1 mRNA levels is clear and seem to support the overall claims, the authors show a trajectory based on one representative dataset. It would strengthen the manuscript to present the mean +\- SD or SEM across several replicates and clarify if statistical analysis was performed. If these data are intended as qualitative support, the phrasing within the manuscript, for example in the abstract, should be adapted accordingly, especially when referring to amplitude changes, pulsing or peaks.

(response)

For the figures originally presented as Figures 2, 3, and 6 (corresponding to Figures 3, 4B, and 7 in the revised manuscript), substantial variability arises due to technical factors inherent to the experiments. For this reason, we did not present these data as mean ± SEM in the main figures. Instead, results from independent biological replicates are provided as Supplementary Figures S1, S2, and S3, respectively. Because no statistical analysis was performed for these time-course data, we have revised the text in the Results section to clearly describe these findings as qualitative observations rather than quantitative comparisons (pages 12–13 lines 277–293, page14 lines 314–317, and page19 lines 422–433).

2. Fold change labeling/ log transformation Figures 4B and S2:

As I understand, the table in Figure 4B is labeled as log2(puf5∆/PUF5). However, the numerical values displayed in Figure 4B and S2 seem to correspond to the linear fold change when compared with supplementary numerical data. It would be helpful to clarify whether the values shown are linear or log-transformed fold changes.

(response)

In the originally submitted manuscript, the tables shown in Figures 4B and S2B (corresponding to Figures 5B and S5A in the revised version) were incorrectly labeled as log₂-transformed values. The numerical values shown were in fact linear fold changes, not log-transformed values, and this was a labelling error. We apologize for this confusion. In the revised manuscript, Figures 5B–C and S5A–B are consistently shown as linear fold changes, and the labeling has been corrected accordingly.

Minor comments:

1.The authors provide cropped example images of western blots in Figures 5, 7 and 8 which appear to be consistent with the quantification and with the claims in the text. It would be helpful to add raw images of western blots in supplementary to improve reproducibility and for readers to assess the quality of the data themselves.

(response)

In accordance with the reviewer’s suggestion, we have provided images of the full gels as supplementary material, following the PLOS ONE gel submission guidelines. These data are included as an attached PDF file to improve transparency and reproducibility.

2.Figures 2,3,6: While not misleading, keeping the scales over similar experiments/assessed samples consistent would be more intuitive for the reader.

(response)

As recommended, we have standardized the scales across comparable experiments and assessed samples to improve clarity and intuitive interpretation for the reader.

3.Figure 7: The R software and relevant packages used for visualization should be cited, this does not influence the quality of the graphs displayed.

(response)

The R software and relevant packages used for data visualization have now been cited as References 45 and 46.

4.The labelling “relative fold change” as labeling of Y-axes throughout the manuscript seems a little redundant. Indicating eventual transformations or the kind of fold change (to reference timepoint, or to control condition) could improve direct interpretation for the reader.

(response)

As suggested, we have revised the Y-axis labels in all figures to provide clearer descriptions, including the reference condition or time point, rather than using the generic term “relative fold change.”

5.Minor grammar or spelling mistakes, including in one subheading, as well as small inconsistencies in tense, or conventional labeling in figures should be addressed during revision to improve the clarity of the manuscript. These do not influence the interpretation of results or the conclusion.

(response)

We carefully reviewed the manuscript and corrected minor grammar, spelling, tense inconsistencies, and conventional figure labeling, including an error in a subheading, to improve overall clarity. These revisions do not affect the interpretation of the results or the conclusions.
