# Supplementary material for: Dual regulatory mechanisms of YGP1 expression in response to glucose availability in Saccharomyces cerevisiae
Source: PLoS One. 2026 Aug 7;21(8):e0355681. doi: 10.1371/journal.pone.0355681 (PMC13450799; doi:10.1371/journal.pone.0355681)
Supplement: Supplementary file 8 [file pone.0355681.s015.docx]

**Our responses to the reviewers’ comments and changes in the revised manuscript**

Title: Dual regulatory mechanisms of *YGP1* expression in response to glucose availability in *Saccharomyces cerevisiae*.

Authors: Megumi Sato, Kaoru Irie, Yasuyuki Suda, Tomoaki Mizuno, Kenji Irie

Regarding the editorial summary points about the data transparency:

There is one remaining issue that needs to be addressed: the clarity of which experiments had technical vs biological replicates as pointed out by Reviewer 1. I would suggest that you add a paragraph in the Methods to clearly say which experiments had biological replicates.

(Our response)

To ensure maximum data transparency as requested, we have now specified the numbers of biological and technical replicates for each experiment in the corresponding figure legends. Briefly, analyses were generally performed using 2–3 independent biological replicates, each evaluated in more than 3 technical replicates, and similar reproducible trends were confirmed across both independent experiments. For these figures, graphs display the mean ± SEM of the tested technical replicates from a single representative biological replicate. Exceptions to this design are as follows:

Figs 1C and 2G: Data represent the mean ± SEM of three independent biological replicates to provide rigorous statistical validation.

Figs 5B–C and 9B, S5 Figs: A single biological replicate was analyzed, as this specific experiment was conducted strictly as a preliminary screening to identify initial candidates.

Further details are provided in the point-by-point responses below.

Reviewer #1

1. Time-course experiments (Figures 3,4,7 and Supplementary Figures 1–3).

While the rephrasing throughout the text and the inclusion of replicates in the supplementary data helped address my previous comments, it is still not entirely clear to me how the authors identify technical or biological replicates, as well as what is meant by “one complete biological replicate” in the revised figure legends. A clearer distinction between technical and biological replicates and their relationship to the presented data would further increase transparency.

(Our response)

We thank the reviewer for this helpful comment. To improve clarity and transparency, we have added a detailed description of the definitions of technical and biological replicates in the Materials and Methods section. We now explicitly indicate in figure legends which type of replicate was used for each experiment and how the presented data were generated.

In addition, the phrase “one complete biological replicate” referred to an independent repetition of the entire time-course experiment, in which all time-point samples were collected and analyzed as a separate experimental set. To avoid ambiguity and improve readability, we have revised the corresponding figure legend text and replaced this expression with a clearer description of the experimental design.

These changes have been incorporated throughout the manuscript to ensure that the relationship between biological replicates, technical replicates, and the presented data is clearly explained.

2. Grammar, spelling and phrasing.

While many language-related concerns were addressed, some minor issues in grammar, spelling, and phrasing appear to remain, including within the newly introduced text after revision. In addition, some repetitive or overly general phrases remain, particularly in the Results and discussion sections. These issues do not influence the general understanding of the main conclusions but addressing them would further improve clarity.

(Our response)

In response to the reviewer’s comments, we carefully reviewed the manuscript and corrected minor grammar, spelling, and redundancy to improve overall clarity. These revisions do not affect the interpretation of the results or the conclusions.
