# Supplementary material for: Dual regulatory mechanisms of YGP1 expression in response to glucose availability in Saccharomyces cerevisiae
Source: PLoS One. 2026 Aug 7;21(8):e0355681. doi: 10.1371/journal.pone.0355681 (PMC13450799; doi:10.1371/journal.pone.0355681)
Supplement: Peer review history

# Peer review: Decision Letter 0

**DOI:** 10.1371/journal.pone.0355681.r001

**Authors:** Arthur J. Lustig

12 Mar 2026

PONE-D-26-01892

Dual regulatory mechanisms of YGP1 expression in response to glucose availability in Saccharomyces cerevisiae

PLOS One

Dear Dr. Irie,

Thank you for submitting your manuscript to PLOS ONE. After careful consideration, we feel that it has merit but does not fully meet PLOS ONE’s publication criteria as it currently stands. Therefore, we invite you to submit a revised version of the manuscript that addresses the points raised during the review process.

**Several issues were raised by the reviewers that must be addressed. The issues of greatest concern are delineated below;**

**1. Experimental Rigor:**

**Major**

a There is no data to rule out a role of the 3' UTR of YGP1. This must to be tested.

b. Test the ability of PUR5 to binding with the YGP1 promoter.

c. The transfer from selective to nonselective media may result in loss of the plasmid. Please test for the plasmid under these conditions.

d. There is a lack of statistical repetition as described below.

**Secondary**

a. Are Figures 4B and S2 plotting linear or log-transformed data? These is not clear.

b. Explain the increase in YGP1 in Figure 8B between 9 and 24 hours.

**Statistical Analysis**

1. While there are three trials for each experiment, what is actually presented in the Figures and why is it represented as fold change rather than primary data?  Please repot using the average values of the replications with the standard deviation. Or are fold different shown because the experimental values are not reproducible?  In this latter case, explain the technical difficulties underlying the lack of reproducibility and present the primary data in context of a Table.. Statistical analysis is a  requirement for publication of quantitative data

**Primary Data**

It is the policy of PLOS One that the primary data for blots must be presented as Supplemental Data. Please provide this data.

**Textual**

1. The manuscript was difficult for the Reviewers to follow due to a lack of background and organization. Please provide a context in which to understand the data as described in the critiques.

Please submit your revised manuscript by Apr 26 2026 11:59PM. If you will need more time than this to complete your revisions, please reply to this message or contact the journal office at plosone@plos.org. When you're ready to submit your revision, log on to https://www.editorialmanager.com/pone/ and select the 'Submissions Needing Revision' folder to locate your manuscript file.

Please include the following items when submitting your revised manuscript:

- A letter that responds to each point raised by the academic editor and reviewer(s). You should upload this letter as a separate file labeled 'Response to Reviewers'.
- A marked-up copy of your manuscript that highlights changes made to the original version. You should upload this as a separate file labeled 'Revised Manuscript with Track Changes'.
- An unmarked version of your revised paper without tracked changes. You should upload this as a separate file labeled 'Manuscript'.

If you would like to make changes to your financial disclosure, please include your updated statement in your cover letter. Guidelines for resubmitting your figure files are available below the reviewer comments at the end of this letter.

If applicable, we recommend that you deposit your laboratory protocols in protocols.io to enhance the reproducibility of your results. Protocols.io assigns your protocol its own identifier (DOI) so that it can be cited independently in the future. For instructions see: https://journals.plos.org/plosone/s/submission-guidelines#loc-laboratory-protocols. Additionally, PLOS ONE offers an option for publishing peer-reviewed Lab Protocol articles, which describe protocols hosted on protocols.io. Read more information on sharing protocols at https://plos.org/protocols?utm_medium=editorial-email&utm_source=authorletters&utm_campaign=protocols.

We look forward to receiving your revised manuscript.

Kind regards,

Arthur J. Lustig, PhD

Academic Editor

PLOS One

Journal Requirements:

When submitting your revision, we need you to address these additional requirements.

1. Please ensure that your manuscript meets PLOS ONE's style requirements, including those for file naming. The PLOS ONE style templates can be found at

https://journals.plos.org/plosone/s/file?id=wjVg/PLOSOne_formatting_sample_main_body.pdf and https://journals.plos.org/plosone/s/file?id=ba62/PLOSOne_formatting_sample_title_authors_affiliations.pdf

2. Thank you for stating in your Funding Statement:

[This research was supported by JSPS KAKENHI Grant Number 22K06074, and 25K09504 (to KI).].

Please provide an amended statement that declares *all* the funding or sources of support (whether external or internal to your organization) received during this study, as detailed online in our guide for authors at http://journals.plos.org/plosone/s/submit-now.  Please also include the statement “There was no additional external funding received for this study.” in your updated Funding Statement.

Please include your amended Funding Statement within your cover letter. We will change the online submission form on your behalf.

3. Please expand the acronym “JSPS KAKENHI” (as indicated in your financial disclosure) so that it states the name of your funders in full.

This information should be included in your cover letter; we will change the online submission form on your behalf.

4. Thank you for stating the following in the Acknowledgments Section of your manuscript:

[We thank all the members of the Molecular Cell Biology Laboratory for valuable discussions. This research was supported by JSPS KAKENHI Grant Number 22K06074, and 25K09504 (to KI).]

We note that you have provided funding information that is currently declared in your Funding Statement. However, funding information should not appear in the Acknowledgments section or other areas of your manuscript. We will only publish funding information present in the Funding Statement section of the online submission form.

Please remove any funding-related text from the manuscript and let us know how you would like to update your Funding Statement. Currently, your Funding Statement reads as follows:

[This research was supported by JSPS KAKENHI Grant Number 22K06074, and 25K09504 (to KI).]

Please include your amended statements within your cover letter; we will change the online submission form on your behalf.

5. Please include your tables as part of your main manuscript and remove the individual files. Please note that supplementary tables (should remain/ be uploaded) as separate "supporting information" files.

6. PLOS ONE now requires that authors provide the original uncropped and unadjusted images underlying all blot or gel results reported in a submission’s figures or Supporting Information files. This policy and the journal’s other requirements for blot/gel reporting and figure preparation are described in detail at https://journals.plos.org/plosone/s/figures#loc-blot-and-gel-reporting-requirements and https://journals.plos.org/plosone/s/figures#loc-preparing-figures-from-image-files. When you submit your revised manuscript, please ensure that your figures adhere fully to these guidelines and provide the original underlying images for all blot or gel data reported in your submission. See the following link for instructions on providing the original image data: https://journals.plos.org/plosone/s/figures#loc-original-images-for-blots-and-gels.

In your cover letter, please note whether your blot/gel image data are in Supporting Information or posted at a public data repository, provide the repository URL if relevant, and provide specific details as to which raw blot/gel images, if any, are not available. Email us at plosone@plos.org if you have any questions.

If the reviewer comments include a recommendation to cite specific previously published works, please review and evaluate these publications to determine whether they are relevant and should be cited. There is no requirement to cite these works unless the editor has indicated otherwise.

[Note: HTML markup is below. Please do not edit.]

Reviewers' comments:

Reviewer's Responses to Questions

**Comments to the Author**

1. Is the manuscript technically sound, and do the data support the conclusions?

The manuscript must describe a technically sound piece of scientific research with data that supports the conclusions. Experiments must have been conducted rigorously, with appropriate controls, replication, and sample sizes. The conclusions must be drawn appropriately based on the data presented.

Reviewer #1: Partly

Reviewer #2: Yes

**********

2. Has the statistical analysis been performed appropriately and rigorously?

Reviewer #1: N/A

Reviewer #2: Yes

**********

3. Have the authors made all data underlying the findings in their manuscript fully available?

The PLOS Data policy requires authors to make all data underlying the findings described in their manuscript fully available without restriction, with rare exception (please refer to the Data Availability Statement in the manuscript PDF file). The data should be provided as part of the manuscript or its supporting information, or deposited to a public repository. For example, in addition to summary statistics, the data points behind means, medians and variance measures should be available. If there are restrictions on publicly sharing data—e.g. participant privacy or use of data from a third party—those must be specified.

Reviewer #1: Yes

Reviewer #2: Yes

**********

4. Is the manuscript presented in an intelligible fashion and written in standard English?

PLOS ONE does not copyedit accepted manuscripts, so the language in submitted articles must be clear, correct, and unambiguous. Any typographical or grammatical errors should be corrected at revision, so please note any specific errors here.

Reviewer #1: Yes

Reviewer #2: Yes

**********

5. Review Comments to the Author

Please use the space provided to explain your answers to the questions above. You may also include additional comments for the author, including concerns about dual publication, research ethics, or publication ethics. (Please upload your review as an attachment if it exceeds 20,000 characters)

Reviewer #1: In this manuscript, Sato et al., investigate the regulatory mechanisms of YGP1 expression in response to glucose availability in Saccharomyces cerevisiae. While the topic is interesting, the data presented do not sufficiently support the author’s conclusions. In particular, the manuscript attempts to link YGP1 expression to glucose availability, while simultaneously introducing multiple regulatory factors (PUF5, MSN2, MSN4, HAA1), cell-cycle phase (G1 and M phase), and acid stress. However, the relationships among these parameters are not clearly defined, resulting in a confusing overall narrative.

The logical flow of the manuscript is unclear, and it is difficult for readers to identify the central question being addressed. In addition, the writing requires substantial professional revision for clarity and coherence. Overall, I recommend major revision.

Major:

1) The relationship between glucose availability and PUF5, MSN2, and MSN4 needs to be clearly defined. The authors should summarize the known roles of these proteins in S. cerevisiae, particularly under glucose-rich versus glucose-depleted conditions. Without this context, the proposed regulatory model lacks mechanistic support, it will be better having those genes expression level along with the glucose availability in S. cerevisiae. The authors should directly present the YGP1 expression data together with the glucose concentration over time, rather than relying on cited literature and the culture time.

2) The conclusion that PUF5 regulates YGP1 via its promoter is not fully supported. The current reporter construct (YGP1 promoter-GFP-ADH1 3’UTR) does not test 3’ UTR-dependent regulation. A construct containing the YGP1 3’UTR should be included. Direct evidence of PUR5 binding with YGP1 promoter (e.g. EMSA) is also needed.

3) The functional relevance of YGP1 should be clarified first. Since the manuscript focus on YGP1 regulation, its physiological role under the tested conditions should be clearly demonstrated before dissecting its regulatory mechanisms.

4) The transfer of plasmid-bearing strains from SC-ura to YPD may result in plasmid loss due to lack of selection (as this method is used to loop out the pCUT plasmid during yeast strains construction). This raises concerns about data reliability and must be clarified.

5) Overall, the manuscript would benefit from a clearer structure: 1) demonstrate the YGP1 expression dynamics with glucose levels; 2) systematically analyze the role of PUF5, HAA1, MSN2, and MSN4 in glucose-dependent regulation; 3) Compare YGP1 expression in different genetic backgrounds, including deletion and overexpression of the regulatory factors; 4) identify and characterization the promoter elements of YGP1; 5) evaluate growth phenotypes of YGP1 mutant strains in different genetic background under different culture conditions. As currently presented, the manuscript appears more focused on PUF5 than on YGP1, which makes the central theme unclear.

Minors:

1) Figure 2 lacks error bars, although the authors state that n = 3 biological replicates were performed.

2) Lines 274–287, The results and discussion in this section may be affected by the problematic culture conditions described above.

3) Line 319 / Figure 8B, What explains the increase in YGP1 expression at 9 h and 24 h? This requires clarification.

4) Line 329, correct “ling-term” to “long-term”.

5) Figure 9F, did the authors test a PUF5 deletion strain combined with YGP1 overexpression?

Reviewer #2: The manuscript provided by Sato et al. investigates the regulation of YGP1 expression under various growth conditions via comparative mutant analysis, promoter deletion mapping, time course expression studies, and stress response assays.

The authors propose a regulatory mechanism in which Puf5 influences YGP1 expression directly and partially via Haa1 during glucose-rich conditions. The stress responsive transcription factors Msn2/4 contribute to YGP1 induction through STRE elements within the YPG1 promoter during growth phases in which glucose availability is limited.

The manuscript follows a clear and logical trajectory, presenting a biologically plausible model of the proposed regulation of YGP1.

Overall, the experiments seem technically sound and the data support the claims, although the manuscript could benefit from clarification in data representation and statistical reporting.

The schematic illustrations of the experimental design (e.g., promoter deletions) and the proposed biological model are clear and helpful for understanding the study and its conclusions.

Major comments:

1. Time course experiments Figure 2,3,6:

The experiments are described as representative data of three experiments, without showing data on replication. In reference to these figures, the authors discuss temporal change.

While I understand that the effect of the mutations discussed on YPG1 mRNA levels is clear and seem to support the overall claims, the authors show a trajectory based on one representative dataset. It would strengthen the manuscript to present the mean +\- SD or SEM across several replicates and clarify if statistical analysis was performed.

If these data are intended as qualitative support, the phrasing within the manuscript, for example in the abstract, should be adapted accordingly, especially when referring to amplitude changes, pulsing or peaks.

2. Fold change labeling/ log transformation Figures 4B and S2:

As I understand, the table in Figure 4B is labeled as log2(puf5∆/PUF5). However, the numerical values displayed in Figure 4B and S2 seem to correspond to the linear fold change when compared with supplementary numerical data. It would be helpful to clarify whether the values shown are linear or log-transformed fold changes.

Minor comments:

1.The authors provide cropped example images of western blots in Figures 5, 7 and 8 which appear to be consistent with the quantification and with the claims in the text. It would be helpful to add raw images of western blots in supplementary to improve reproducibility and for readers to assess the quality of the data themselves.

2.Figures 2,3,6: While not misleading, keeping the scales over similar experiments/assessed samples consistent would be more intuitive for the reader.

3.Figure 7: The R software and relevant packages used for visualization should be cited, this does not influence the quality of the graphs displayed.

4.The labelling “relative fold change” as labeling of Y-axes throughout the manuscript seems a little redundant. Indicating eventual transformations or the kind of fold change (to reference timepoint, or to control condition) could improve direct interpretation for the reader.

5.Minor grammar or spelling mistakes, including in one subheading, as well as small inconsistencies in tense, or conventional labeling in figures should be addressed during revision to improve the clarity of the manuscript. These do not influence the interpretation of results or the conclusion.

**********

6. PLOS authors have the option to publish the peer review history of their article (what does this mean?). If published, this will include your full peer review and any attached files.

If you choose “no”, your identity will remain anonymous but your review may still be made public.

**Do you want your identity to be public for this peer review?**  For information about this choice, including consent withdrawal, please see our Privacy Policy.

Reviewer #1: No

Reviewer #2: No

**********

[NOTE: If reviewer comments were submitted as an attachment file, they will be attached to this email and accessible via the submission site. Please log into your account, locate the manuscript record, and check for the action link "View Attachments". If this link does not appear, there are no attachment files.]

To ensure your figures meet our technical requirements, please review our figure guidelines: https://journals.plos.org/plosone/s/figures

You may also use PLOS’s free figure tool, NAAS, to help you prepare publication quality figures: https://journals.plos.org/plosone/s/figures#loc-tools-for-figure-preparation.

NAAS will assess whether your figures meet our technical requirements by comparing each figure against our figure specifications.

# Author comment: Author response to Decision Letter 1

**DOI:** 10.1371/journal.pone.0355681.r002

27 Apr 2026

Our responses to the reviewers’ comments and changes in the revised manuscript

Title: Dual regulatory mechanisms of YGP1 expression in response to glucose availability in Saccharomyces cerevisiae.

Authors: Megumi Sato, Kaoru Irie, Yasuyuki Suda, Tomoaki Mizuno, Kenji Irie

Regarding the editorial summary points about the Statistical Analysis:

We have clarified the nature of the data shown in each figure in the corresponding figure legends.

Regarding the use of fold change values throughout the manuscript, mRNA expression levels were quantified by RT-qPCR using the ΔΔCt method, and Rq values relative to a defined reference sample were therefore presented as fold changes. The underlying Ct values for all experiments are provided in a numerical data file (S1 Data set). For protein quantification by Western blotting, absolute signal intensities varied substantially between experiments due to unavoidable technical variability, such as repeated use of primary and secondary antibodies. Because these raw signal intensities are not directly comparable across experiments, protein levels were normalized to the endogenous control Pgk1, and fold changes relative to a defined reference sample were used for presentation.

Taken together, fold change representation was intentionally employed to enable meaningful comparison across experiments, rather than reflecting a lack of reproducibility. Where applicable, biological replicates are shown and described, and the nature of the analysis (quantitative vs. qualitative) is explicitly stated in the manuscript.

Further details are provided in the point-by-point responses below.

Reviewer #1

Major:

1) The relationship between glucose availability and PUF5, MSN2, and MSN4 needs to be clearly defined. The authors should summarize the known roles of these proteins in S. cerevisiae, particularly under glucose-rich versus glucose-depleted conditions. Without this context, the proposed regulatory model lacks mechanistic support, it will be better having those genes expression level along with the glucose availability in S. cerevisiae.

The authors should directly present the YGP1 expression data together with the glucose concentration over time, rather than relying on cited literature and the culture time.

(response)

Based on the reviewer’s comments, we added descriptions of previously reported functions of Puf5 to the Results section (page 10, lines 203–209) and incorporated additional information regarding Msn2/Msn4 into the relevant sections of the Introduction (page3, lines 47–56) and Results (pages 21–22, lines 503–508).

In addition, we measured glucose concentrations in the culture medium during time-course cultivation, and the data are presented in Figure 1C. The corresponding results are described in the Results section (page9, lines 193–195 and page10, lines 215–216).

2) The conclusion that PUF5 regulates YGP1 via its promoter is not fully supported. The current reporter construct (YGP1 promoter-GFP-ADH1 3’UTR) does not test 3’ UTR-dependent regulation. A construct containing the YGP1 3’UTR should be included. Direct evidence of PUR5 binding with YGP1 promoter (e.g. EMSA) is also needed.

(response)

In response to the reviewer’s comments, we investigated the contribution of the YGP1 3’UTR by constructing MCM2promoter–GFP–ADH1 3’UTR and MCM2promoter–GFP–YGP1 3’UTR reporter constructs. GFP reporter expression was analyzed in wild-type and puf5Δ mutant cells. We found that neither reporter exhibited a significant difference in expression in the puf5Δ mutant compared with wild-type cells. These results indicate that the regulatory target of Puf5 is likely the YGP1 promoter, rather than its 3’UTR. These results are shown in Figure 2G and are described in the Results section (page 11, lines 245–251).

Furthermore, to examine whether Puf5 directly binds to the YGP1 promoter, we performed a ChIP assay to assess Puf5 association with a 60-nucleotide region of the YGP1 promoter (−600 to −540). However, we were unable to detect enrichment of this chromatin region in our ChIP analysis. These results suggest that regulation of YGP1 by Puf5 is likely indirect, rather than mediated by direct binding of Puf5 to the YGP1 promoter.

3) The functional relevance of YGP1 should be clarified first. Since the manuscript focus on YGP1 regulation, its physiological role under the tested conditions should be clearly demonstrated before dissecting its regulatory mechanisms.

(response)

In accordance with the reviewer’s suggestions, we revised the structure of the Results section. We now begin the Results by describing previously reported and proposed functions of Ygp1 (page9, lines 182–188), followed by presentation of our analysis of the regulatory mechanisms governing YGP1 expression.

4) The transfer of plasmid-bearing strains from SC-ura to YPD may result in plasmid loss due to lack of selection (as this method is used to loop out the pCUT plasmid during yeast strains construction). This raises concerns about data reliability and must be clarified.

(response)

Following the reviewer’s suggestion, we assessed plasmid retention in plasmid-bearing strains. Plasmid carriage reates after 24 h of cultivation in YPD medium, following pre-culture in SC-Ura, are shown in Tables 4, 5, and 7. All strains exhibited plasmid retention rates above 95%, with no significant differences among strains.

5) Overall, the manuscript would benefit from a clearer structure: 1) demonstrate the YGP1 expression dynamics with glucose levels; 2) systematically analyze the role of PUF5, HAA1, MSN2, and MSN4 in glucose-dependent regulation; 3) Compare YGP1 expression in different genetic backgrounds, including deletion and overexpression of the regulatory factors; 4) identify and characterization the promoter elements of YGP1; 5) evaluate growth phenotypes of YGP1 mutant strains in different genetic background under different culture conditions. As currently presented, the manuscript appears more focused on PUF5 than on YGP1, which makes the central theme unclear

(response)

Based on the reviewer’s comments, we revised the overall structure of the manuscript. To more clearly distinguish the regulatory mechanisms operating during the logarithmic phase and at the diauxic shift, we first present the time-course expression profile of YGP1 in the revised manuscript and figures. The subsequent structure largely follows that of the originally submitted version; however, we revised the text where appropriate to improve clarity and ensure a coherent logical flow.

Minors:

1) Figure 2 lacks error bars, although the authors state that n = 3 biological replicates were performed.

(response)

In the originally submitted version, Figure 2 (revised as Figure 3) showed representative values from three technical replicates. Reproducibility was confirmed using an additional independent biological replicate, and these data are now presented in Figure S1.

2) Lines 274–287, The results and discussion in this section may be affected by the problematic culture conditions described above.

(response)

As described above, plasmid retention rates did not differ significantly among strains under the culture conditions used. Therefore, we regard that the influence of culture conditions has been appropriately considered, and the interpretation presented in the manuscript remains valid.

3) Line 319 / Figure 8B, What explains the increase in YGP1 expression at 9 h and 24 h? This requires clarification.

As shown in Figure 1C, glucose concentrations in the culture medium during cultivation of wild-type and puf5Δ cells were already close to depletion at the 9h time point. We therefore consider that slight differences in the initial glucose concentrations during medium preparation may have resulted in minor variations in the timing of glucose depletion. This explanation has been added to the Results section (page22, lines 514–520).

4) Line 329, correct “ling-term” to “long-term”.

(response)

We carefully checked the manuscript and corrected this typographical error on page 25 line 572.

5) Figure 9F, did the authors test a PUF5 deletion strain combined with YGP1 overexpression?

(response)

In response to the reviewer’s comments, we examined the effect of a YEp‑YGP1 on cell viability during long-term cultivation of the puf5Δ mutant. However, as shown in the graph below, we did not observe an obvious recovery of viability by the YEp YGP1. However, we could not confirm whether the plasmid overexpressed a fully functional form of YGP1. In addition, the reduced viability of the puf5Δ ygp1Δ double mutant was consistently reproducible (n=2). Therefore, we speculate that Ygp1 plays a supportive role in long-term survival under puf5Δ conditions.

# Reviewer 2

Major comments:

1. Time course experiments Figure 2,3,6:

The experiments are described as representative data of three experiments, without showing data on replication. In reference to these figures, the authors discuss temporal change. While I understand that the effect of the mutations discussed on YPG1 mRNA levels is clear and seem to support the overall claims, the authors show a trajectory based on one representative dataset. It would strengthen the manuscript to present the mean +\- SD or SEM across several replicates and clarify if statistical analysis was performed. If these data are intended as qualitative support, the phrasing within the manuscript, for example in the abstract, should be adapted accordingly, especially when referring to amplitude changes, pulsing or peaks.

(response)

For the figures originally presented as Figures 2, 3, and 6 (corresponding to Figures 3, 4B, and 7 in the revised manuscript), substantial variability arises due to technical factors inherent to the experiments. For this reason, we did not present these data as mean ± SEM in the main figures. Instead, results from independent biological replicates are provided as Supplementary Figures S1, S2, and S3, respectively. Because no statistical analysis was performed for these time-course data, we have revised the text in the Results section to clearly describe these findings as qualitative observations rather than quantitative comparisons (pages 12–13 lines 277–293, page14 lines 314–317, and page19 lines 422–433).

2. Fold change labeling/ log transformation Figures 4B and S2:

As I understand, the table in Figure 4B is labeled as log2(puf5∆/PUF5). However, the numerical values displayed in Figure 4B and S2 seem to correspond to the linear fold change when compared with supplementary numerical data. It would be helpful to clarify whether the values shown are linear or log-transformed fold changes.

(response)

In the originally submitted manuscript, the tables shown in Figures 4B and S2B (corresponding to Figures 5B and S5A in the revised version) were incorrectly labeled as log₂-transformed values. The numerical values shown were in fact linear fold changes, not log-transformed values, and this was a labelling error. We apologize for this confusion. In the revised manuscript, Figures 5B–C and S5A–B are consistently shown as linear fold changes, and the labeling has been corrected accordingly.

Minor comments:

1.The authors provide cropped example images of western blots in Figures 5, 7 and 8 which appear to be consistent with the quantification and with the claims in the text. It would be helpful to add raw images of western blots in supplementary to improve reproducibility and for readers to assess the quality of the data themselves.

(response)

In accordance with the reviewer’s suggestion, we have provided images of the full gels as supplementary material, following the PLOS ONE gel submission guidelines. These data are included as an attached PDF file to improve transparency and reproducibility.

2.Figures 2,3,6: While not misleading, keeping the scales over similar experiments/assessed samples consistent would be more intuitive for the reader.

(response)

As recommended, we have standardized the scales across comparable experiments and assessed samples to improve clarity and intuitive interpretation for the reader.

3.Figure 7: The R software and relevant packages used for visualization should be cited, this does not influence the quality of the graphs displayed.

(response)

The R software and relevant packages used for data visualization have now been cited as References 45 and 46.

4.The labelling “relative fold change” as labeling of Y-axes throughout the manuscript seems a little redundant. Indicating eventual transformations or the kind of fold change (to reference timepoint, or to control condition) could improve direct interpretation for the reader.

(response)

As suggested, we have revised the Y-axis labels in all figures to provide clearer descriptions, including the reference condition or time point, rather than using the generic term “relative fold change.”

5.Minor grammar or spelling mistakes, including in one subheading, as well as small inconsistencies in tense, or conventional labeling in figures should be addressed during revision to improve the clarity of the manuscript. These do not influence the interpretation of results or the conclusion.

(response)

We carefully reviewed the manuscript and corrected minor grammar, spelling, tense inconsistencies, and conventional figure labeling, including an error in a subheading, to improve overall clarity. These revisions do not affect the interpretation of the results or the conclusions.

Attachment Submitted filename: Sato_response-to-reviewers-IR20260424MS.docx

# Peer review: Decision Letter 1

**DOI:** 10.1371/journal.pone.0355681.r003

**Authors:** Arthur J. Lustig

8 Jun 2026

PONE-D-26-01892R1

Dual regulatory mechanisms of YGP1 expression in response to glucose availability in Saccharomyces cerevisiae

PLOS One

Dear Dr. Irie,

Thank you for submitting your manuscript to PLOS ONE. After careful consideration, we feel that it has merit but does not fully meet PLOS ONE’s publication criteria as it currently stands. Therefore, we invite you to submit a revised version of the manuscript that addresses the points raised during the review process.

There is one remaining issue that needs to be addressed: the clarity of which experiments had technical vs biological replicates as pointed out by Reviewer 1. I would suggest that you add a paragraph in the Methods to clearly say which experiments had biological replicates.

Please submit your revised manuscript by Jul 23 2026 11:59PM. If you will need more time than this to complete your revisions, please reply to this message or contact the journal office at plosone@plos.org. When you're ready to submit your revision, log on to https://www.editorialmanager.com/pone/ and select the 'Submissions Needing Revision' folder to locate your manuscript file.

Please include the following items when submitting your revised manuscript:

- A letter that responds to each point raised by the academic editor and reviewer(s). You should upload this letter as a separate file labeled 'Response to Reviewers'.
- A marked-up copy of your manuscript that highlights changes made to the original version. You should upload this as a separate file labeled 'Revised Manuscript with Track Changes'.
- An unmarked version of your revised paper without tracked changes. You should upload this as a separate file labeled 'Manuscript'.

If you would like to make changes to your financial disclosure, please include your updated statement in your cover letter. Guidelines for resubmitting your figure files are available below the reviewer comments at the end of this letter.

If applicable, we recommend that you deposit your laboratory protocols in protocols.io to enhance the reproducibility of your results. Protocols.io assigns your protocol its own identifier (DOI) so that it can be cited independently in the future. For instructions see: https://journals.plos.org/plosone/s/submission-guidelines#loc-laboratory-protocols. Additionally, PLOS ONE offers an option for publishing peer-reviewed Lab Protocol articles, which describe protocols hosted on protocols.io. Read more information on sharing protocols at https://plos.org/protocols?utm_medium=editorial-email&utm_source=authorletters&utm_campaign=protocols.

As the corresponding author, your ORCID iD is verified in the submission system and will appear in the published article. PLOS supports the use of ORCID, and we encourage all coauthors to register for an ORCID iD and use it as well. Please encourage your coauthors to verify their ORCID iD within the submission system before final acceptance, as unverified ORCID iDs will not appear in the published article. *Only*  the individual author can complete the verification step; PLOS staff *cannot*  verify ORCID iDs on behalf of authors.

We look forward to receiving your revised manuscript.

Kind regards,

Arthur J. Lustig, PhD

Academic Editor

PLOS One

Journal Requirements:

1. If the reviewer comments include a recommendation to cite specific previously published works, please review and evaluate these publications to determine whether they are relevant and should be cited. There is no requirement to cite these works unless the editor has indicated otherwise.

2. Please review your reference list to ensure that it is complete and correct. If you have cited papers that have been retracted, please include the rationale for doing so in the manuscript text, or remove these references and replace them with relevant current references. Any changes to the reference list should be mentioned in the rebuttal letter that accompanies your revised manuscript. If you need to cite a retracted article, indicate the article’s retracted status in the References list and also include a citation and full reference for the retraction notice.

[Note: HTML markup is below. Please do not edit.]

Reviewers' comments:

Reviewer's Responses to Questions

**Comments to the Author**

1. If the authors have adequately addressed your comments raised in a previous round of review and you feel that this manuscript is now acceptable for publication, you may indicate that here to bypass the “Comments to the Author” section, enter your conflict of interest statement in the “Confidential to Editor” section, and submit your "Accept" recommendation.

Reviewer #1: All comments have been addressed

Reviewer #2: (No Response)

**********

2. Is the manuscript technically sound, and do the data support the conclusions?

The manuscript must describe a technically sound piece of scientific research with data that supports the conclusions. Experiments must have been conducted rigorously, with appropriate controls, replication, and sample sizes. The conclusions must be drawn appropriately based on the data presented.

Reviewer #1: Yes

Reviewer #2: Yes

**********

3. Has the statistical analysis been performed appropriately and rigorously?

Reviewer #1: Yes

Reviewer #2: Yes

**********

4. Have the authors made all data underlying the findings in their manuscript fully available?

The PLOS Data policy requires authors to make all data underlying the findings described in their manuscript fully available without restriction, with rare exception (please refer to the Data Availability Statement in the manuscript PDF file). The data should be provided as part of the manuscript or its supporting information, or deposited to a public repository. For example, in addition to summary statistics, the data points behind means, medians and variance measures should be available. If there are restrictions on publicly sharing data—e.g. participant privacy or use of data from a third party—those must be specified.

Reviewer #1: Yes

Reviewer #2: Yes

**********

5. Is the manuscript presented in an intelligible fashion and written in standard English?

PLOS ONE does not copyedit accepted manuscripts, so the language in submitted articles must be clear, correct, and unambiguous. Any typographical or grammatical errors should be corrected at revision, so please note any specific errors here.

Reviewer #1: Yes

Reviewer #2: Yes

**********

6. Review Comments to the Author

Please use the space provided to explain your answers to the questions above. You may also include additional comments for the author, including concerns about dual publication, research ethics, or publication ethics. (Please upload your review as an attachment if it exceeds 20,000 characters)

Reviewer #1: (No Response)

Reviewer #2: I thank the authors for the responses and the revision of the manuscript. Most of my comments have been addressed. However, there are two remaining concerns that I would like to point out:

1. Time-course experiments (Figures 3,4,7 and Supplementary Figures 1-3).

While the rephrasing throughout the text and the inclusion of replicates in the supplementary data helped address my previous comments, it is still not entirely clear to me how the authors identify technical or biological replicates, as well as what is meant by “one complete biological replicate” in the revised figure legends. A clearer distinction between technical and biological replicates and their relationship to the presented data would further increase transparency.

2. Grammar, spelling and phrasing.

While many language-related concerns were addressed, some minor issues in grammar, spelling, and phrasing appear to remain, including within the newly introduced text after revision. In addition, some repetitive or overly general phrases remain, particularly in the Results and discussion sections. These issues do not influence the general understanding of the main conclusions but addressing them would further improve clarity.

**********

7. PLOS authors have the option to publish the peer review history of their article (what does this mean?). If published, this will include your full peer review and any attached files.

If you choose “no”, your identity will remain anonymous but your review may still be made public.

**Do you want your identity to be public for this peer review?**  For information about this choice, including consent withdrawal, please see our Privacy Policy.

Reviewer #1: No

Reviewer #2: No

**********

[NOTE: If reviewer comments were submitted as an attachment file, they will be attached to this email and accessible via the submission site. Please log into your account, locate the manuscript record, and check for the action link "View Attachments". If this link does not appear, there are no attachment files.]

To ensure your figures meet our technical requirements, please review our figure guidelines: https://journals.plos.org/plosone/s/figures

You may also use PLOS’s free figure tool, NAAS, to help you prepare publication quality figures: https://journals.plos.org/plosone/s/figures#loc-tools-for-figure-preparation.

NAAS will assess whether your figures meet our technical requirements by comparing each figure against our figure specifications.

# Author comment: Author response to Decision Letter 2

**DOI:** 10.1371/journal.pone.0355681.r004

3 Jul 2026

Our responses to the reviewers’ comments and changes in the revised manuscript

Title: Dual regulatory mechanisms of YGP1 expression in response to glucose availability in Saccharomyces cerevisiae.

Authors: Megumi Sato, Kaoru Irie, Yasuyuki Suda, Tomoaki Mizuno, Kenji Irie

Regarding the editorial summary points about the data transparency:

There is one remaining issue that needs to be addressed: the clarity of which experiments had technical vs biological replicates as pointed out by Reviewer 1. I would suggest that you add a paragraph in the Methods to clearly say which experiments had biological replicates.

(Our response)

To ensure maximum data transparency as requested, we have now specified the numbers of biological and technical replicates for each experiment in the corresponding figure legends. Briefly, analyses were generally performed using 2–3 independent biological replicates, each evaluated in more than 3 technical replicates, and similar reproducible trends were confirmed across both independent experiments. For these figures, graphs display the mean ± SEM of the tested technical replicates from a single representative biological replicate. Exceptions to this design are as follows:

Figs 1C and 2G: Data represent the mean ± SEM of three independent biological replicates to provide rigorous statistical validation.

Figs 5B–C and 9B, S5 Figs: A single biological replicate was analyzed, as this specific experiment was conducted strictly as a preliminary screening to identify initial candidates.

Further details are provided in the point-by-point responses below.

Reviewer #1

1. Time-course experiments (Figures 3,4,7 and Supplementary Figures 1–3).

While the rephrasing throughout the text and the inclusion of replicates in the supplementary data helped address my previous comments, it is still not entirely clear to me how the authors identify technical or biological replicates, as well as what is meant by “one complete biological replicate” in the revised figure legends. A clearer distinction between technical and biological replicates and their relationship to the presented data would further increase transparency.

(Our response)

We thank the reviewer for this helpful comment. To improve clarity and transparency, we have added a detailed description of the definitions of technical and biological replicates in the Materials and Methods section. We now explicitly indicate in figure legends which type of replicate was used for each experiment and how the presented data were generated.

In addition, the phrase “one complete biological replicate” referred to an independent repetition of the entire time-course experiment, in which all time-point samples were collected and analyzed as a separate experimental set. To avoid ambiguity and improve readability, we have revised the corresponding figure legend text and replaced this expression with a clearer description of the experimental design.

These changes have been incorporated throughout the manuscript to ensure that the relationship between biological replicates, technical replicates, and the presented data is clearly explained.

2. Grammar, spelling and phrasing.

While many language-related concerns were addressed, some minor issues in grammar, spelling, and phrasing appear to remain, including within the newly introduced text after revision. In addition, some repetitive or overly general phrases remain, particularly in the Results and discussion sections. These issues do not influence the general understanding of the main conclusions but addressing them would further improve clarity.

(Our response)

In response to the reviewer’s comments, we carefully reviewed the manuscript and corrected minor grammar, spelling, and redundancy to improve overall clarity. These revisions do not affect the interpretation of the results or the conclusions.

Attachment Submitted filename: Sato_response-to-reviewers_20260702.docx

# Peer review: Decision Letter 2

**DOI:** 10.1371/journal.pone.0355681.r005

**Authors:** Arthur J. Lustig

26 Jul 2026

Dual regulatory mechanisms of YGP1 expression in response to glucose availability in Saccharomyces cerevisiae

PONE-D-26-01892R2

Dear Dr. Irie,

We’re pleased to inform you that your manuscript has been judged scientifically suitable for publication and will be formally accepted for publication once it meets all outstanding technical requirements.

Within one week, you’ll receive an e-mail detailing the required amendments. When these have been addressed, you’ll receive a formal acceptance letter and your manuscript will be scheduled for publication.

An invoice will be generated when your article is formally accepted. Please note, if your institution has a publishing partnership with PLOS and your article meets the relevant criteria, all or part of your publication costs will be covered. Please make sure your user information is up-to-date by logging into Editorial Manager at Editorial Manager® and clicking the ‘Update My Information' link at the top of the page. For questions related to billing, please contact billing support.

If your institution or institutions have a press office, please notify them about your upcoming paper to help maximize its impact. If they’ll be preparing press materials, please inform our press team as soon as possible -- no later than 48 hours after receiving the formal acceptance. Your manuscript will remain under strict press embargo until 2 pm Eastern Time on the date of publication. For more information, please contact onepress@plos.org.

Kind regards,

Arthur J. Lustig, PhD

Academic Editor

PLOS One

Additional Editor Comments (optional):

Reviewers' comments:

Reviewer's Responses to Questions

**Comments to the Author**

1. If the authors have adequately addressed your comments raised in a previous round of review and you feel that this manuscript is now acceptable for publication, you may indicate that here to bypass the “Comments to the Author” section, enter your conflict of interest statement in the “Confidential to Editor” section, and submit your "Accept" recommendation.

Reviewer #2: All comments have been addressed

**********

2. Is the manuscript technically sound, and do the data support the conclusions?

The manuscript must describe a technically sound piece of scientific research with data that supports the conclusions. Experiments must have been conducted rigorously, with appropriate controls, replication, and sample sizes. The conclusions must be drawn appropriately based on the data presented.

Reviewer #2: Yes

**********

3. Has the statistical analysis been performed appropriately and rigorously?

Reviewer #2: Yes

**********

4. Have the authors made all data underlying the findings in their manuscript fully available?

The PLOS Data policy requires authors to make all data underlying the findings described in their manuscript fully available without restriction, with rare exception (please refer to the Data Availability Statement in the manuscript PDF file). The data should be provided as part of the manuscript or its supporting information, or deposited to a public repository. For example, in addition to summary statistics, the data points behind means, medians and variance measures should be available. If there are restrictions on publicly sharing data—e.g. participant privacy or use of data from a third party—those must be specified.

Reviewer #2: Yes

**********

5. Is the manuscript presented in an intelligible fashion and written in standard English?

PLOS ONE does not copyedit accepted manuscripts, so the language in submitted articles must be clear, correct, and unambiguous. Any typographical or grammatical errors should be corrected at revision, so please note any specific errors here.

Reviewer #2: Yes

**********

6. Review Comments to the Author

Please use the space provided to explain your answers to the questions above. You may also include additional comments for the author, including concerns about dual publication, research ethics, or publication ethics. (Please upload your review as an attachment if it exceeds 20,000 characters)

Reviewer #2: (No Response)

**********

7. PLOS authors have the option to publish the peer review history of their article (what does this mean?). If published, this will include your full peer review and any attached files.

If you choose “no”, your identity will remain anonymous but your review may still be made public.

**Do you want your identity to be public for this peer review?**  For information about this choice, including consent withdrawal, please see our Privacy Policy.

Reviewer #2: No

**********

# Editor report: Acceptance letter

**DOI:** 10.1371/journal.pone.0355681.r006

**Authors:** Arthur J. Lustig

PONE-D-26-01892R2

PLOS One

Dear Dr. Irie,

I'm pleased to inform you that your manuscript has been deemed suitable for publication in PLOS One. Congratulations! Your manuscript is now being handed over to our production team.

At this stage, our production department will prepare your paper for publication. This includes ensuring the following:

* All references, tables, and figures are properly cited

* All relevant supporting information is included in the manuscript submission,

* There are no issues that prevent the paper from being properly typeset

You will receive further instructions from the production team, including instructions on how to review your proof when it is ready. Please keep in mind that we are working through a large volume of accepted articles, so please give us a few days to review your paper and let you know the next and final steps.

Lastly, if your institution or institutions have a press office, please let them know about your upcoming paper now to help maximize its impact. If they'll be preparing press materials, please inform our press team within the next 48 hours. Your manuscript will remain under strict press embargo until 2 pm Eastern Time on the date of publication. For more information, please contact onepress@plos.org.

You will receive an invoice from PLOS for your publication fee after your manuscript has reached the completed accept phase. If you receive an email requesting payment before acceptance or for any other service, this may be a phishing scheme. Learn how to identify phishing emails and protect your accounts at https://explore.plos.org/phishing.

If we can help with anything else, please email us at customercare@plos.org.

Thank you for submitting your work to PLOS ONE and supporting open access.

Kind regards,

PLOS ONE Editorial Office Staff

on behalf of

Dr. Arthur J. Lustig

Academic Editor

PLOS One
